# Mitochondrial genomes revisited: why do different lineages retain different genes?

**DOI:** 10.1186/s12915-024-01824-1

**Published:** 2024-01-25

**Authors:** Anzhelika Butenko, Julius Lukeš, Dave Speijer, Jeremy G. Wideman

**Affiliations:** 1 Institute of Parasitology, Biology Centre, Czech Academy of Sciences, České Budějovice (Budweis), Czech Republic; 2 Faculty of Science, University of Ostrava, Ostrava, Czech Republic; 3 Faculty of Sciences, University of South Bohemia, České Budějovice (Budweis), Czech Republic; 4 Medical Biochemistry, Amsterdam UMC, University of Amsterdam, Amsterdam, The Netherlands; 5 Center for Mechanisms of Evolution, Biodesign Institute, School of Life Sciences, Arizona State University, Tempe, USA

**Keywords:** CoRR hypothesis, Evolutionary cell biology, Endosymbiont gene transfer, Mitochondrial DNA, Mitochondrial evolution, Mitochondrial mutation rates

## Abstract

The mitochondria contain their own genome derived from an alphaproteobacterial endosymbiont. From thousands of protein-coding genes originally encoded by their ancestor, only between 1 and about 70 are encoded on extant mitochondrial genomes (mitogenomes). Thanks to a dramatically increasing number of sequenced and annotated mitogenomes a coherent picture of why some genes were lost, or relocated to the nucleus, is emerging. In this review, we describe the characteristics of mitochondria-to-nucleus gene transfer and the resulting varied content of mitogenomes across eukaryotes. We introduce a ‘burst-upon-drift’ model to best explain nuclear-mitochondrial population genetics with flares of transfer due to genetic drift.

**Supplementary Information:**

The online version contains supplementary material available at 10.1186/s12915-024-01824-1.

## Introduction: diversity of mitochondrial coding capacities

**Fig. 1:**
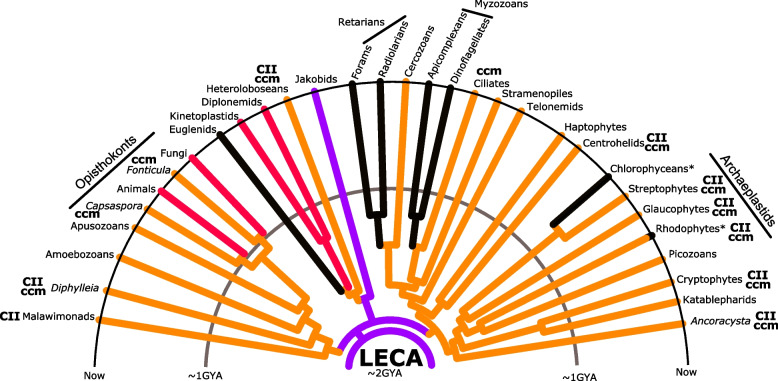
Most lineages retain relatively stable mitochondrial genomic coding capacities. A cladogram starting with LECA depicts the differential evolution of mitochondrial genomic coding capacities in widely divergent eukaryotic lineages. Though exceptions to these trends are present in various groups, several lineages retain mitochondrial genome coding capacities typical for their clade. CII indicates retention of (some) complex II subunits; ccm indicates retention of subunits of the multicomponent bacteria-derived c-type cytochrome biogenesis system. Purple lineage: largest set of mitochondrial genes; orange lineages: retention of an intermediate number of mitochondrial genes; red lineages: retention of the ‘core set’ of mitochondrial genes only; black lineages: more extensive mitochondrial gene transfer and loss including transfer or loss of all ribosomal genes—usually contains fragmented rRNAs. Asterisks indicate lineages displaying large variations in mitochondrial gene content. For further information, see the main text

**Fig. 2:**
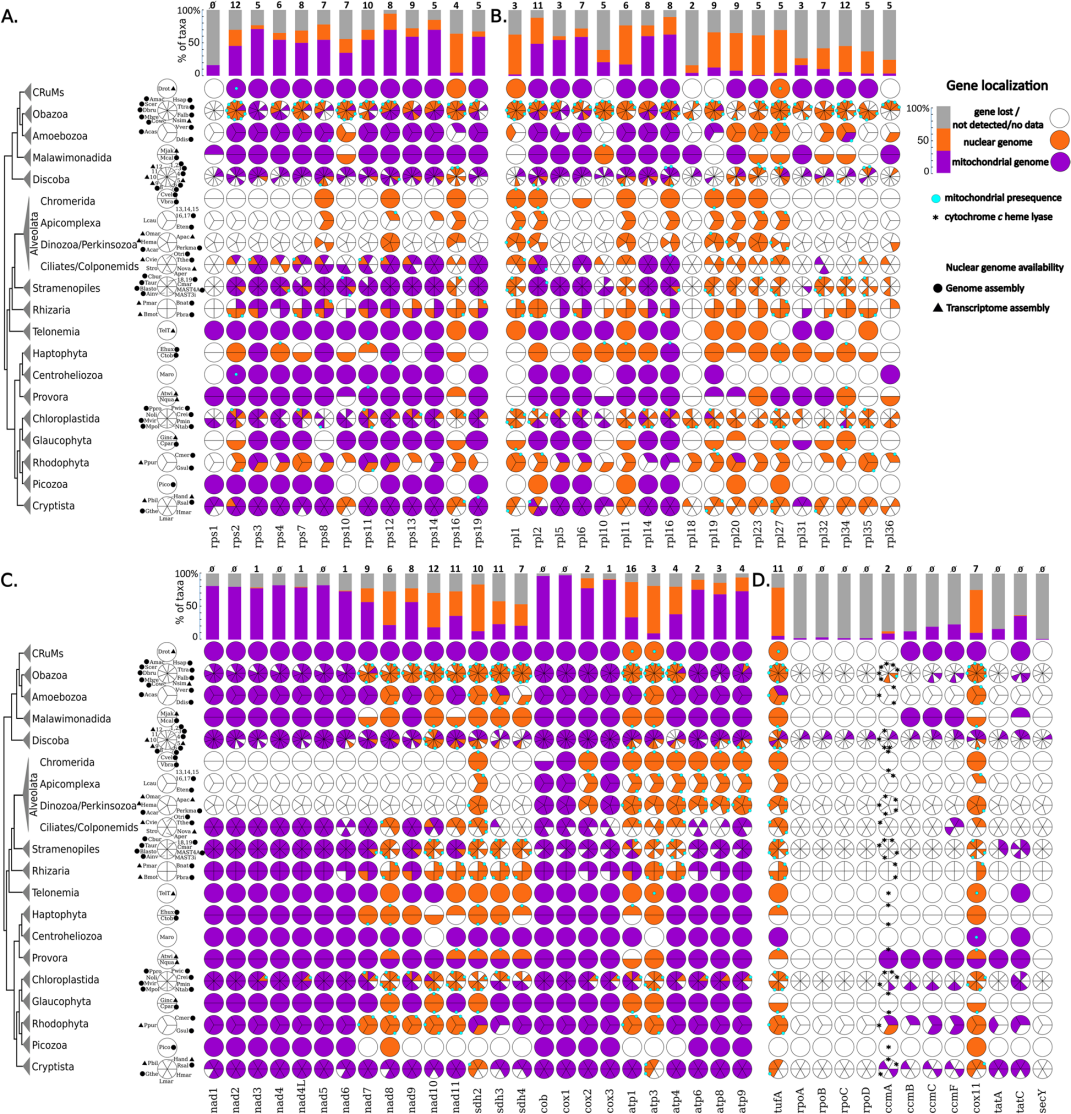
Mitochondria-to-nucleus gene transfer is relatively rare. Coulson plots showing the distribution of genes encoding components of small (**A**) and large (**B**) subunits of mitoribosomes, as well as electron transport chain (**C**) and other (**D**) proteins across mitochondrial and nuclear genomes of the representatives of major eukaryotic supergroups. Genes retained in the mitogenomes are depicted in purple, nucleus-encoded genes are in orange, and those lost or not detected are in white. Within each eukaryotic group, the species with identical gene distribution patterns were unified into one sector of a circle. Species with available genome and transcriptome assemblies are marked with back circles and triangles, respectively. The cladogram reflecting the phylogenetic relationships among major eukaryotic lineages is based on [9] and [10]. The bar charts at the top of the Coulson plots indicate the percentage of investigated taxa where the respective gene is encoded in mitochondrial (purple) and nuclear genome (orange) or lost/not detected. The numerical values above the bar charts correspond to the number of presumably independent mitochondrion-to-nucleus gene transfer events in the evolution of eukaryotes. Proteins predicted to possess a mitochondrial presequence by at least two out of three bioinformatic tools (MitoFates, TargetP, and TPpred3) are marked with cyan circles. The presence of a gene encoding cytochrome *c* heme-lyase in the nuclear genome is indicated with an asterisk over the ccmA gene charts. Coulson plots were produced with a Coulson plot generator [11]. For compact representation, some species were assigned numbers as follows: 1, *Naegleria gruberi*; 2, *Naegleria fowleri*; 3, *Andalucia godoyi*; 4, *Reclinomonas americana*; 5, *Euglena gracilis*; 6, Euglenozoa ‘SAG EU17/18’; 7, *Diplonema papillatum*; 8, *Trypanosoma brucei*; 9, *Tsukubamonas globosa*; 10, *Heterolobosea* sp. ‘BB2’; 11, *Acrasis kona*; 12, *Pharyngomonas kirbyi*; 13, *Plasmodium falciparum*; 14, *Babesia microti*; 15, *Cyclospora cayetanensis*; 16, *Theileria annulata*; 17, *Toxoplasma gondii*; 18, *Phaeodactylum tricornutum*; and 19, *Thalassiosira pseudonana*. The species abbreviations: Acar, *Amphidinium carterae*; Acas, *Acanthamoeba castellanii*; Ainv, *Aphanomyces invadans*; Amac, *Allomyces macrogynus*; Apac, *Alexandrium pacificum*; Aper, *Acavomonas peruviana*; Atwi, *Ancoracysta twista*; Blasto, *Blastocystis* sp.; Bmot, *Brevimastigomonas motovehiculus*; Bnat, *Bigelowiella natans*; Cbur, *Cafeteria burkhardae*; Cmar, *Chattonella marina*; Cmer, *Cyanidioschyzon merolae*; Cowc, *Capsaspora owczarzaki*; Cpar, *Cyanophora paradoxa*; Crei, *Chlamydomonas reinhardtii*; Ctob, *Chrysochromulina tobinii*; Cvel, *Chromera velia*; Cvie, *Colponema vietnamica*; Dbru, *Dekkera bruxellensis*; Ddis, *Dictyostelium discoideum*; Drot, *Diphylleia rotans*; Ehux, *Emiliania huxleyi*; Eten, *Eimeria tenella*; Falb, *Fonticula alba*; Ginc, *Glaucocystis incrassata*; Gsul, *Galdieria sulphuraria*; Gthe, *Guillardia theta*; Hand, *Hemiselmis andersenii*; Hema, *Hematodinium* sp.; Hmar, *Hemiarma marina*; Hsap, *Homo sapiens*; Lcau, *Leucocytozoon caulleryi*; Lmar, *Leucocryptos marina*; Maro, *Marophrys* sp.; MAST, marine stramenopile; Mbre, *Monosiga brevicollis*; Mcal, *Malawimonas californiana*; Mjak, *Malawimonas jakobiformis*; Mpol, *Marchantia polymorpha*; Mvir, *Mesostigma viride*; Noli, *Nephroselmis olivacea*; Nova, *Nyctotherus ovalis*; Nqua, *Nibbleromonas quarantinus*; Nsim, *Nuclearia simplex*; Ntab, *Nicotiana tabacum*; Omar, *Oxyrrhis marina*; Otri, *Oxytricha trifallax*; Pbil, *Palpitomonas bilix*; Pbra, *Plasmodiophora brassicae*; Perkma, *Perkinsus marinus*; Pico, *Picozoa* sp.; Pmar, *Paracercomonas marina*; Pmin, *Pedinomonas minor*; Ppro, *Pycnococcus provasolii*; Ppur, *Porphyra purpurea*; Pwic, *Prototheca wickerhamii*; Rsal, *Rhodomonas salina*; Scer, *Saccharomyces cerevisiae*; Stro, *Strombidium* sp.; Taur, *Thraustochytrium aureum*; TelT, *Telonemid* sp.; The, *Tetrahymena thermophila*; Ttra, *Thecamonas trahens*; Vbra, *Vitrella brassicaformis*; and Vver, *Vermamoeba vermiformis*

The mitochondria arose from an endosymbiotic (alphaproteo-like) bacterium during early eukaryotic evolution [1, 2]. Excluding iterative instances of primary endosymbioses (as proposed in [3]), we can conclude that the last eukaryotic common ancestor (LECA), living about 1.8 billion years ago, contained an aerobic organelle. The mitochondrial organelle of LECA would probably have looked much like extant mitochondria in certain protists [4]. Just as in modern eukaryotes, the main function of mitochondria in LECA would have been oxidative phosphorylation via the electron transport chain (ETC) and ATP synthase. Based on a comparative analysis of eukaryotic diversity, we can infer that the mitochondrial genome (mitogenome) of LECA would have encoded at least 69 proteins including components of the ETC and ATP synthase, ribosome components, and a few proteins involved in protein translocation, and heme maturation [1]. While one or two protist lineages retain this complexity nearly in full, most eukaryotes exhibit a further reduced complement of ancestral mitochondria-encoded proteins, indicating many genes have either been lost outright or transferred to the nucleus via endosymbiont gene transfer (EGT). Several parallel losses or EGTs of mitochondria-encoded genes (mitogenes) have occurred leading to some lineages retaining very similar sets. For example, although animals and fungi contain nearly the same set of mitogenes, their reduction occurred via parallel losses and parallel transfers to the nucleus (Fig. 1). The apparent abundance of parallel mitochondrial EGTs makes it tempting to speculate that they occur relatively often and are likely selectively beneficial. However, this speculation is unfounded as mitogene content is largely stable, even for billions of years, in most major lineages (Figs. 1 and 2) [5]. Thus, mitogene transfers do not happen with regularity and need not always be beneficial (with certain genes being retained or relocated in a haphazard fashion). So, if mitochondria-to-nucleus gene transfer is not always beneficial, how do we explain the diversity of mitochondrial coding capacities across the tree of eukaryotes? Though we focus on the mitochondria, we believe most of our arguments can also be applied to chloroplasts and other, more recent endosymbioses in which EGT has occurred; see also [6–8].

## The evolution of mitogenomes: questions answered and questions outstanding

Before we address the question of how to explain mitochondrial coding diversity, we must get a few others asked, answered, and out of the way. The simplest question about mitogenomes is: Why do mitogenomes exist at all? The simplest answer is that mitogenomes exist because the alphaproteobacterial-like ancestor of the organelle itself possessed a genome [12, 13]. The next questions are: Why did over 99% of ancestral mitogenes move to the nucleus or disappear, and why did the final 1% remain? An answer to why mitogenes have disappeared comes from models demonstrating that loss or nuclear transfer of mitogenes is bioenergetically beneficial [6], and transfer might be evolutionarily inevitable [14]. Provided the assumptions are correct in these models, given enough time, evolution will proceed to its logical endpoint—the complete absence of mitogenomes. But no lineage of aerobic eukaryotes exists that lacks this genome [15]—and the only report suggesting the existence of a mitochondrion lacking a mitogenome [16] is likely an artefact [17, 18]. Answers to why so few mitogenes have been retained simply try to explain the exceptions that fail to follow the trends predicted by bioenergetics and population genetics. Thus, explanations for mitogene retention are simply exceptional cases of constraint—that is, EGTs fail when nuclear expression and subsequent transport of its product into mitochondria is biophysically challenging [19, 20], or when (lineage-specific) adaptations require mitogene retention for specialized transcriptional or translational regulatory mechanisms [21, 22]. Problematically, these broad-scale answers fail to provide complete explanations for the rich diversity of mitogenomes.

Here, we review the various explanations and shed light on the possible reasons for the diverse evolutionary trajectories of mitogenomes. We will focus on explaining major patterns of retention and nuclear migration of mitogenes that are not easily accommodated by conventional explanations. After outlining patterns of gene retention and loss in mitogenomes, we will proceed to further discuss the merits, as well as the limits in explanatory power, of the hydrophobicity and co-location for redox regulation (CoRR) hypotheses in the light of the latest data. In line with data presented in [19, 23], we conclude that both theories partially explain why certain genes are retained in some mitogenomes but, on their own, cannot adequately account for the diversity of coding content that persists across eukaryotic lineages.

We contend that retention of mitogenes is almost always selectively beneficial and therefore successful transfer of mitogenes can only occur when mitochondrial mutation rates are high in small populations. As suggested previously [24], initial mitochondria-to-nucleus transfers are likely not immediately beneficial. Therefore, positive selection cannot be directly implicated in the transfer event as a given mitochondria-to-nucleus EGT is usually deleterious. Thus, mitogene transfers likely occur in a nearly neutral situation where the fixation of slightly deleterious EGTs becomes possible. In such cases, the likelihood of transfer depends almost entirely on population genetic parameters: nuclear and mitochondrial mutation rates and their respective population sizes.

## Mitochondrial gene content variation: from 100 to 1

As the proverbial ‘powerhouse of the cell’, all aerobic and even some anaerobic mitochondria retain genes encoding proteins required to produce a functioning ETC. However, the coding capacity of mitogenomes varies from as few as 1 or 2 protein-coding genes and fragmented rRNAs [25] to as many as 67 protein-coding genes with 3 bacteria-like (SSU, LSU, and 5S) rRNAs and dozens of tRNAs [4]. A vast diversity of mitogenomes with intermediate coding capacities also persists (Figs. 1 and 2). However, rather than a steady and predictable transfer of genes to the nucleus, it seems that most lineages have lost genes in punctuated bursts (Fig. 1). Alternatively, this could be indicative of undiscovered intermediary lineages that have possibly gone extinct. In animals, fungi, and some protists, a so-called core set of these proteins is retained in mitogenomes (red lineages in Fig. 1; detailed depictions in Fig. 2). These core proteins constitute central, often highly hydrophobic, components of the ETC complexes I, III, and IV; and subunits of ATP synthase [19, 20]. Such is the case in our own mitochondria, typical of animal mitogenomes [26]. Some components of the translational machinery are retained universally across eukaryotes (SSU and LSU rRNA), whereas other components can be lost (5S rRNA and tRNAs), while possible transfer (tRNA) is being debated [27–29].

The mitogene content of most major eukaryotic lineages (orange lineages in Fig. 1) is larger than that of animals or fungi and includes several genes encoding ribosomal proteins, additional ETC subunits (such as components of succinate dehydrogenase—complex II), bacterial-derived cytochrome *c* maturation machinery components (so-called ccm systems), and a translocase of unknown function. Jakobids—an order of free-living, heterotrophic, bi-flagellar protists—retain the largest mitochondrial coding repertoire of almost 70 protein-coding genes, including a (most likely) ancestral bacterial RNA polymerase [30], which has been replaced by a ‘viral-type’ RNA polymerase encoded in the nucleus of all other eukaryotes (purple lineage in Fig. 1). By linking our knowledge of mitogenomes to recent advances [2] in dating eukaryotic divergences, we can see that major mitogene content evolution occurs rarely, often marking the origin of major eukaryotic lineages, with relatively few changes in the last billion years.

Protists constitute the vast majority of major eukaryote lineages [31]. Until recently, the evolutionary relationships within eukaryotic diversity might have seemed rather messy. However, major parts of the eukaryotic tree have recently stabilized [32], even though new kingdom-level lineages are continuing to be discovered [5, 33–36]. Essentially, animals, fungi, and plants make up branches in the Opisthokonts and Archaeplastids, and the rest of the tree is occupied by diverse ‘kingdoms’ of protists. The general trend is that many lineages retain a similar set of genes in their mitogenomes (purple pies in Fig. 2), and many lineages have similar sets that are encoded in their nuclear genomes (orange pies in Fig. 2). However, when you look closely at these data (represented as a dendrogram in Additional File 1), it becomes clear that, in some cases, to arrive at this pattern, several EGTs needed to occur. For example, within ATP synthase components, at least 16 EGTs can be identified for *atp1*, compared to only 3 or 4 for *atp3* and *atp4*—though *atp3* was transferred very early, whereas *atp4* was transferred later. It is unclear what explains the varied frequency and timing of these transfers, though we attempt an explanation below.

Perhaps some mitochondrial proteins (mitoproteins) are more amenable to being retargeted to mitochondria after nuclear transfer. To determine how many nucleus-encoded mitoproteins have detectable N-terminal mitochondrial targeting signals (MTSs), we used a number of programs to predict MTSs in genes encoded in both nuclear and mitochondrial genomes. Unsurprisingly, many nucleus-encoded ETC and ATP synthase components have clear MTSs, but none is present on mitochondria-encoded versions (small cyan circles in Fig. 2). However, in the case of mitochondrial ribosomal (mitoribosomal) proteins, though most animal and fungal representatives had detectable MTSs, a considerable number of mitoribosomal proteins of protists do not. Such retargeting of particular mitoproteins to the mitochondria in the absence of a canonical MTS could be a widespread phenomenon. In a few cases (e.g. *Dictyostelium* Rps8 and Rpl34, heterolobosean Rpl16, ciliate Rpl2 and Rpl16, haptophyte Rps12 and Rpl16, and a handful of others), even mitochondria-encoded proteins contain (internal) MTSs, corroborating the idea that some mitoribosomal proteins are primed for mitochondrial retargeting. This idea has recently been experimentally explored in budding yeast [37].

Conspicuous are the large, though somewhat fragmented, proportions of lineages that retain mitochondria-encoded mitoribosomal proteins (Fig. 2A, B, purple pies). As long as the mitochondria require components of the ETC and retain their own genomes, mitoribosomes must be constructed in the organelle to produce mitochondria-encoded proteins. Although all extant lineages retain SSU and LSU rRNAs encoded in their mitogenomes, several lineages have transferred all mitoribosomal proteins to the nucleus, including myzozoans (apicomplexans + dinoflagellates), retarians (radiolarians and foraminifera) [38], chlamydomonadean algae, and euglenids (black lineages in Fig. 1; lineages with mostly white/orange pie segments in Fig. 2A, B). Some of the lineages that have lost or transferred all or most mitoribosomal proteins contain highly fragmented rRNAs [39–42]. We attempt to provide an explanation for how mitoribosomal proteins might be lost or transferred en masse below, which could also explain the conserved rRNA fragmentation in various lineages.

## Global benefits to transfer: bioenergetic efficiency supplies a fitness benefit to mitochondria-to-nucleus EGT

The alphaproteobacterial genome from which mitogenomes evolved likely contained thousands of protein-coding genes. Disuse is one reason for genomes to become streamlined, but are there others? It turns out that maintaining unfavourable per-cell gene copy numbers (e.g. resulting from a disproportionally large number of mitogenomes per cell) can be energetically burdensome to a host-endosymbiont consortium. There can be substantial cost savings associated with gene loss, even if the gene is still marginally useful, and hence, if a protein is no longer needed in an endosymbiont genome, there is a bioenergetic benefit in its loss [6]. Similarly, because there are many copies of mitogenomes in a given cell and usually only one nuclear genome, there are cost savings associated with mitogene transfer to the nucleus. On average, genes that have been lost entirely from eukaryotes have lower expression levels in a model alphaproteobacterium, whereas those that were transferred to the nucleus had medium expression levels, and retained genes had the highest expression levels [6]. Thus, for most genes, the energy saved by transfer exceeds the costs of protein targeting, import, and assembly. Because many genes are still encoded in mitogenomes, the benefits to transfer are not enough to outweigh the constraints on transfer in these cases.

Our comparative analysis of mitogenomes revealed that certain mitogenes are more amenable to transfer than others, for instance, 16 transfers of *atp1* and at least 17 replacements of the bacteria-derived cytochrome *c* maturation machinery. The loss of 4 bacteria-derived ccm mitogenes in exchange for the gain of a single mitochondria-targeted nucleus-encoded cytochrome *c* heme lyase (CCHL) probably provides a generous bioenergetic benefit. Perhaps there is a similar benefit to transferring *atp1*, and some components of mitoribosomes or complex I that are regularly transferred. However, the fact that some genes that are present in several taxa are rarely (e.g. < 6 transfers: *rps3*, *rps4*, *rpl5*, *rpl6*, *atp4*, *atp8*) or even never (*rps1*) transferred to the nucleus suggests that major constraints to their transfer exist. Interestingly, when one of these proteins goes to the nucleus, the others are not far behind (Fig. 2). How are structural and functional constraints suddenly broken allowing the mass transfer of so many mitoproteins?

Beyond the economy of bioenergetics, we know of no other measurable benefits to transfer. It is possible that nuclear regulation could be beneficial, but it is unclear how this would provide immediate selective benefit [43]. An oft-cited reason for transferring to the nucleus is protection from the harmful mutational effects of the reactive oxygen species (ROS) produced by the mitochondrial ETC. This, coupled with less efficient DNA repair, might drive mutation pressure at the population level, possibly partially explaining large-scale migration and loss, pre-LECA [44–46]. We believe that many biologists, us included until recently, intuitively equate nuclear protection from mutation as the primary selective force behind EGT. However, nuclear protection is a metaphor for the nuclear mutation rate being lower than the mitochondrial mutation rate in a given species, leading to the assumption that protection equals a fitness benefit. It is true that differential mutation vulnerability can act like selection [47, 48]. But mutation rates are population-level variables that must be considered independently of individual allele-specific fitness coefficients.

To clarify, the intuitive argument of nuclear protection treats protection from mutational decay as an individual trait. However, it is a population-level trait that cannot impact individual-level fitness. Thus, there is no *direct* selective benefit for a gene to escape organellar mutation pressure. While we further explain this notion in the penultimate section, we will first expand on cell biological constraints to transfer.

## Cell biological constraints: nuclear expression of mitochondria-encoded genes results in fitness defects

**Fig. 3:**
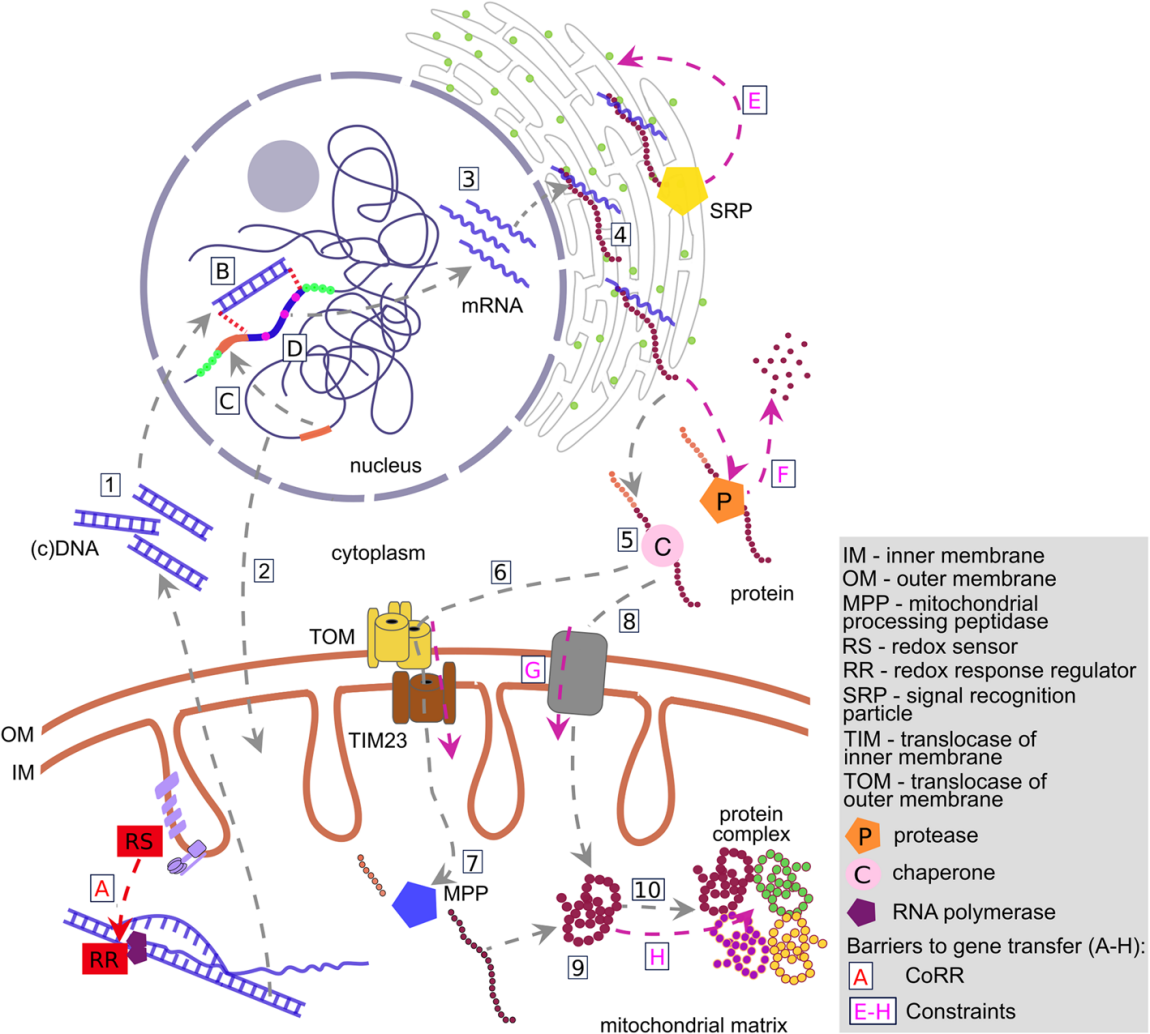
Obstacles to functional mitochondria-to-nucleus gene transfer. Subsequent steps in the transfer of mitochondrial (mt) genes to the nuclear genomes are indicated with (numbered) grey arrows. Obstacles to transfer are marked by letters (A–H) and arrows (CoRR hypothesis: red; constraints hypothesis: magenta). Genetic material can be transferred from the mitochondria to the nuclei as DNA or cDNA (1) during fission/fusion events, mitochondrial lysis or mitophagy, the transfer process being facilitated by organelle proximity and vacuole formation, protecting DNA fragments from cytoplasmic nucleases. Entrenched mitochondrial gene regulation can be a barrier to transfer. A specific case of regulation of expression by redox sensors and redox response regulators forms the crux of the CoRR hypothesis. Gene transfer in the opposite direction (nucleus-to-mitochondrial genome (2)) is extremely rare (so far, only demonstrated in corals and plants). Upon (c)DNA transfer, integration into a suitable genome locus (B) without disrupting essential genes or causing genome instability has to occur. Some genes will gain mitochondrial targeting signals (orange segments) from other nuclear genes (C) or formed de novo. The newly transferred gene should gain regulatory elements (green dots) enabling efficient expression (D) or be transcribed polycistronically with a nuclear gene. The process of codon optimization might contribute to establishing optimal expression levels of the now nucleus-encoded gene (D). For some organisms, mitochondrial RNA editing/deviations of the genetic code might represent extra obstacles to effective gene transfer (D). Upon successful completion of the steps mentioned, mRNA is synthesized and exported to the cytoplasm (3), where proteins are synthesized (4) on cytosolic ribosomes (olive green circles). Proteins with highly hydrophobic transmembrane domains, >  ~ 120 amino acids (length threshold for proteins to be recognized by the signal recognition particle), would thus be co-translationally miss-targeted to the ER (E). Newly synthesized proteins might be degraded by cytoplasmic peptidases (F) or bind chaperones (5) and be directed to mitochondria. Proteins enter mitochondria using a pre-sequence mediated pathway involving TOM and TIM23 complexes (6), with subsequent cleavage of pre-sequences by mitochondrial processing peptidase (7), or via other mechanisms (8). High protein hydrophobicity might represent a significant barrier to traversing the mitochondrial membranes (G). Following a successful transport into the mitochondria, proteins assume native conformations (9) and in some cases are incorporated into their respective protein complexes (10). Protein complex assembly processes normally involve highly ordered sets of steps, often requiring co-translational incorporation of subunits, potentially representing an additional barrier (H) for functional gene transfer to the nucleus

Mitogenes cannot be easily transferred to the nucleus because of several cell biological constraints (Fig. 3). The main explanation for mitogene retention is therefore not because it is the best place for them to reside, but because evolution has followed a path from which there is no turning back. Most constraints can be overcome relatively easily, given enough time and luck. While physical transfer of mitochondrial DNA to the nuclear genome occurs relatively easily [49] and mechanisms for gene activation and protein targeting are well-understood [50, 51], functional mitochondria-to-nucleus transfer followed by nuclear retention remains exceedingly rare. The converse, functional DNA transfer into the mitochondria is even more rare [52–54]. The unidirectionality of EGT provides an infinite number of future opportunities for transfers to occur.

It is important to note that all constraints to transfer can probably be broken, but some constraints are much tighter than others. For example, the genetic code disparity can prevent mitogene transfer [55]; however, many organelles, including deeply branching lineages of mitochondria, use the canonical genetic code and retain many genes. Therefore, code disparity can only be lineage-specific and not a universal reason for mitogene retention [19]. Another discarded explanation speculated about the relatively infrequent physical transfer of DNA from organelles to the nucleus. But we now know physical transfers can occur regularly, reshape chromosomes [56], and even become rapidly fixed in some lineages (e.g. *Homo sapiens* and *Arabidopsis thaliana*) [57, 58]. It has also been suggested that gene activation and gene product targeting may represent major barriers [44]. However, transcription can occur spontaneously, and de novo evolution of a promoter or the insertion of a mitogene downstream of an appropriate promoter is easy to imagine, while intricate examples of protein retargeting have been identified in plants [59]. We will not comment further on these seemingly surmountable constraints.

Experimental transfers in humans and yeast suggest that some constraints are almost insurmountable, however. When the human mitoproteins encoded by *atp6* and *cox3* are re-designed for nuclear expression (by emulating the structural properties of nucleus-encoded homologues from the alga *Chlamydomonas reinhardtii* [60, 61]), the proteins seem to be targeted to the mitochondria. Yet, these redesigned proteins fail to functionally integrate into their cognate ETC [62] or perhaps get stuck in their passage across the two mitochondrial membranes. In human cells, *atp6* and *atp8* can sometimes successfully rescue mitochondrial mutants [63], whereas *nad1*, *nad2*, *nad4*, *cox1*, *cob*, and *cox3* have all failed [62]. In yeast, successful experimental transfers have been reported for *atp8* and *rps3* and with hydrophobicity-reducing modifications for *cox2* and *atp9* [64–66], though it is important to note that rescued strains all exhibit some defects. Experimental transfer of *cob* has failed [67], while transfers of *cox1*, *cox3*, or *atp6* have not been published. All successful experimental transfers have analogous natural transfers (e.g. *rps3*, *cox2*, *atp8*, and *atp9*) (Fig. 2). There are extreme cases in parasites, in which even genes encoding very hydrophobic proteins such as *cox1* and *cob* have been transferred to the nucleus, but these organisms likely contain mitochondria with very low ETC activity [68].

In the next section, we focus on formalizing two major explanations for why genes are retained in organellar genomes: the hydrophobicity and the CoRR hypotheses. We present the key tenets and predictions of each hypothesis followed by experimental observations and conclude with general statements about the extent to which the hypotheses explain the full gamut of observations. We note that both hypotheses explain why nuclear relocalization results in fitness defects. In other words, both hypotheses describe barriers to relocalization, not necessarily the benefits of mitochondrial localization. After outlining the biological constraints, we elaborate on how these constraints might be broken.

## Gene-specific constraints on effective organellar localization: the hydrophobicity hypothesis

Recent insight has shown that the best predictor of protein-coding gene retention in the mitochondrion is hydrophobicity and/or how central a protein is to a given multiprotein complex [19, 20, 23, 69]. These empirical predictors help us shed light on the explanations for retention. However, do current observations uphold or refute the hydrophobicity hypothesis? In this section, we attempt to formalize this hypothesis to determine how well available observations conform to predictions.

**The hydrophobicity hypothesis:** Selective constraints on targeting and transport of highly hydrophobic proteins have played a major role in modulating the evolution of mitogenomes, which have been maintained to ensure the correct localization of these highly hydrophobic membrane proteins [51, 70–73].

**Prediction 1:** Genes encoding highly hydrophobic proteins are subjected to the strongest selective constraints and are rarely transferred to the nuclear genomes [72].

**Observation 1:** Cob and Cox1 are among the most hydrophobic mitoproteins [67] and represent a minimum set of mitochondria-encoded proteins currently known [74], except in some highly reduced, parasitic apicomplexans [68]. It is worth noting that not all organellar genes encode only hydrophobic proteins [75]. Thus, this explanation for the mitogenome retention of genes is at least incomplete. In the rare cases in which genes encoding extremely hydrophobic proteins are successfully transferred, major modifications are often observed, such as gene splitting or mutations that reduce hydrophobicity. However, such splitting can also occur with mitogenes that are not that hydrophobic [76].

**Prediction 2:** Hydrophobic membrane proteins encoded by mitogenomes would be recognized by the signal recognition particle (or by the components of unconventional pathways) and mis-targeted to the endoplasmic reticulum if they were nucleus-encoded [71, 72, 77].

**Observation 2:** Allotopic gene-expression experiments show that hydrophobic proteins encoded in the human mitogenome are directed to the endoplasmic reticulum when expressed in the nucleus, except for the hydrophilic Atp8 [72, 73]. Protein mis-targeting is associated with changes in cell morphology [73], while artificial reduction of a protein’s hydrophobicity allows its import into mitochondria [78].

**Conclusion:** The predictions from the hydrophobicity hypothesis largely hold true. Genes encoding hydrophobic proteins are constrained to be expressed in mitochondria unless hydrophobicity is naturally or artificially reduced. The hydrophobicity hypothesis fails to explain why relatively hydrophilic proteins are retained in mitogenomes.

## Gene-specific constraints on effective regulation: the CoRR hypothesis

A popular explanation for mitogene retention is the co-location for redox regulation (CoRR) hypothesis [21, 79]. In essence, it maintains that the retention of a mitogenome is required because genes which encode central parts of the ETC must have a certain ‘response readiness’ to adjust to changes in the local redox state (e.g. signalled by ROS). The implications are that, for such a response readiness, local genomes are absolutely necessary. The model posits the existence of a complete redox regulatory system functioning within the original membrane-bound compartment [21]. However, the lack of a broadly conserved redox regulatory pathway influencing mitochondrial expression in line with the CoRR hypothesis adds to the paucity of causal links supporting it. Of note, small RNAs and peptides (encoded by either the nucleus or mitochondrion) have so far been mostly overlooked [80–82]. Although mitochondrial transcriptional activation seems to be a plausible mechanism for the CoRR, little to no comparative investigations have been performed [83], and the extent of conservation of these processes across eukaryotes is not known. Again, do current observations uphold or refute the CoRR hypothesis? In this section, we attempt to formalize the hypothesis to determine how well its predictions conform to accumulated observations.

**The CoRR hypothesis:** The reason for the persistence of chloroplast and mitochondrial genomes lies in the selective advantage of subcellular co-localization of specific genes with their products, enabling direct and rapid redox control of gene expression (e.g. to minimize dangerous ROS formation) [21, 22, 79, 84]. The original version of this hypothesis suggested that an ancestral control mechanism existed and has proliferated in extant eukaryotes. A modified version of the CoRR hypothesis would accept that an ancestral version may not have existed, but different mechanisms may have evolved in various eukaryotic lineages thereby individually constraining transfer in a lineage-specific manner.

**Prediction 1:** Subsets of proteins encoded in the mitogenomes should be relatively small and relatively constant [79], as long as they are involved in redox reactions.

**Observation 1: **Generally speaking, genes playing a more central role in bioenergetic supply are retained more often [7]. Gene content in mitogenomes varies rather widely (~ 30-fold differences): from 1 or 2 protein-coding genes in certain alveolates [68, 74] to 67 in the jakobid *Reclinomonas americana* [30]. Some mitogenomes (e.g. those of diplonemids, kinetoplastids, lycophytes, retarians, and apicomplexans) totally lack tRNA genes [38, 85–87], while their full set is still encoded in the mitogenomes of some jakobids, plants, fungi, algae, and mammals [4, 88]. Ribosomal RNA genes (mt-SSU and mt-LSU) are always retained, although they demonstrate remarkable size differences and occasionally undergo fragmentation, while the distribution of mt-5S rRNA is patchy [1, 51, 89]. However, the overall reduction in the ETC protein-coding (but not mitoribosomal) gene content shows some correlation with the loss of classic respiration capacity [15, 68]. If this is the case, then a slight modification of the CoRR hypothesis remains consistent with the existing data. The mitochondria that require high expression of certain genes (e.g. in organisms needing highly efficient ATP generation) will always contain a genome and be even less likely to transfer the remaining genes.

**Prediction 2:** An irreducible core set of ETC components which are subject to redox control must be encoded in the mitogenome. This requirement may disappear when the organelle ceases to perform its bioenergetic role. Thus, the loss of mitogenomes can occur in anaerobic organisms [21, 79]; however, vectorial electron transport without a mitogenome is not possible [21].

**Observation 2:** All aerobic mitochondria retain mitogenomes, while some anaerobes indeed lose them. Still, some anaerobic and hydrogen-producing mitochondria retain them [1]. For example, the ciliate *Nyctotherus ovalis* has a hydrogen-producing, anaerobic mitochondrion which possesses a genome with a gene content similar to that of aerobic ciliates, including some ETC components [90, 91]. In this regard, thus far, all findings are still compatible with the CoRR hypothesis.

**Prediction 3:** Symbiotic ancestors of the mitochondria carried into the host cell a set of ETC components and the regulatory systems that place the synthesis of key components under the regulatory control of redox potential. The expression of genes retained in the mitochondria must be influenced by oxidants, such as ROS and reductants [79], but the precise mechanisms are allowed to diverge. This would be disallowed by the unmodified CoRR hypothesis.

**Observation 3:** Redox regulatory mechanisms of gene expression are relatively well-studied in chloroplasts (e.g. transcription regulation via chloroplast sensor kinase [92]), while for the mitochondria, it is known that redox reagents have certain effects on protein synthesis, although particular mechanisms and redox sensors are not well defined [22]. There are indications that mechanisms regulating the mitogene expression might differ even between relatively closely related organisms, such as mammals and yeast [93].

**Conclusion:** If we consider the CoRR hypothesis as a model in which local redox control is especially important when molecular oxygen is the final electron acceptor (aerobic mitochondria) or molecular oxygen is created (chloroplasts), the overall organellar gene content seems compatible with its predictions, but individual cases remain enigmatic. Aside from the complete loss of a mitogenome in a respiring eukaryote, it seems impossible to refute current (modified) versions of the CoRR hypothesis. Like the hydrophobicity hypothesis, the CoRR hypothesis fails to explain why genes that are not under co-locational redox regulation are retained in mitogenomes. We need to move away from gene-level explanations towards species- or lineage-level explanations.

## Species-level considerations: benefits minus constraints

An important recent paper puts together both the ‘pushes and pulls’ of mitochondria-to-nucleus gene transfer in a new mathematical model [7]. The model balances bioenergetics and mutation pressure as phenomena that push genes out of mitogenomes with factors such as hydrophobicity and superior regulation that pull genes, causing them to stay put. The modelling by these authors reveals that organisms that experience highly varied environments should require a more direct and constant control over mitochondrial gene regulation. These data are compelling; however, explaining lineage-level patterns seems beyond the reach of the model. Whereas the calculations can clarify the balance of selective pressures felt by particular eukaryotes during their evolutionary history, they cannot explain the billion-year-old trends that we seek to explain here. To reiterate, the model of [7] can be used to help explain and predict the selective pressures on a given population of eukaryotic organisms (i.e. a species) and thus be used as a predictive tool for the relative ease with which genes might be transferred from the mitochondria to the nuclei. However, taken in isolation, the model cannot explain why certain lineages retain fewer mitogenes than others.

For example, several photosynthetic lineages (e.g. chlamydomonads, dinoflagellates, and to a lesser extent certain rhodophytes) contain extremely reduced mitogenomes. These lineages defy the expectations of the model, which suggests that photosynthetic organisms with diurnal cycles would retain larger organellar gene complements. Furthermore, some parasites including the anaerobic stramenopile *Blastocystis* retain mitogenomes larger than those of animals and fungi [94], see also Fig. 2. Nonetheless, the implementation of this model in a population genetic framework (see directly below) will no doubt be fruitful in future investigations.

## Lineage-level considerations: population genetics and the burst-upon-drift (BUD) model of mitogenome evolution

While the CoRR and hydrophobicity hypotheses offer broad explanations for why a particular core set of genes are retained in mitogenomes, they do not explain (i) why such variable sets of mitogenes persist, (ii) how/why transferred genes become fixed in the nucleus, and (iii) the relative paucity of lineages with intermediate coding content sister to lineages with reduced mitogenomes (i.e. the ‘spurt-like’ evolution of mitogene migration). If these hypotheses and models are insufficient, then what is missing to make sense of the available observations? We suggest that an extension of results obtained with early population genetic models [14, 95] can explain the data more fully, exceptions included.

**Fig. 4:**
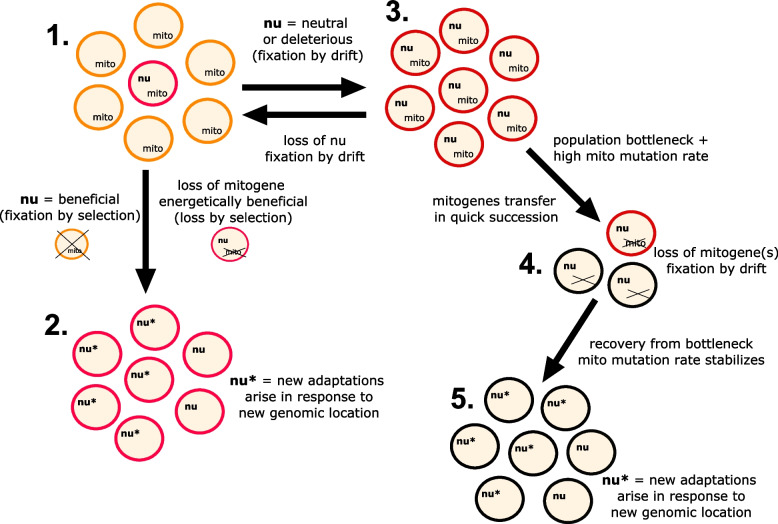
Burst-upon-drift (BUD) model: Small population sizes and high mitochondrial mutation rates can lead to fixation of slightly deleterious mitochondria-to-nucleus gene transfers. Path 1→ 2 represents mitochondria to nucleus transfer by adaptive mechanisms. Path 1 → 3 → 4 → 5 represents neutral transfers via the BUD model. (1) A mitogene is transferred to the nucleus (nu) and is transcribed, translated, and effectively targeted to the mitochondria (red cell in between orange cells). (2) The newly nuclear mitogene (nu) is beneficial and sweeps to fixation in a population due to natural selection, while the mitochondrial mitogene (mito) is lost because of bioenergetic benefit. New adaptations (nu*) will evolve in response to the new genomic location of the previous mitogene. (3) If the newly nuclear mitogene is neutral or mildly detrimental, the transfer can be fixed in the population by drift. In this situation, it is possible that the (mito)gene acquires moderate mutations leading to the sub-functionalization of the gene duplicates and their subsequent retention. (4) Loss of the mitogene may be fixed by drift if the mitochondrial mutation rate is high in a small population. In certain situations, this can occur even though there is a fitness cost caused by retaining only the nuclear mitogene. In these cases, several genes may transfer in quick succession leading to many fewer genes being encoded in the mitochondrial genome (black cells). (5) After the recovery from the population bottleneck, new adaptations (nu*) will evolve in response to the new genomic location of the previous mitogene. Ovals depict individual cells; colour changes of contours reflect changed cells (when compared with cells from a previous step). The colour code is consistent with the lineages in Fig. 1

First, we will consider what happens in the case of a beneficial transfer. Imagine that a mitogene finds its way into the nucleus and is successfully transcribed, translated, and targeted to the mitochondrion (Fig. 4 (1)). It could be that this duplicated intermediate state is deleterious or beneficial. For now, however, we assume that intercompartmental duplications are completely neutral. If this nucleus-encoded mitogene functions better than the mitochondria-encoded one, the nuclear version of the gene will sweep to fixation due to selection (Fig. 4 (2)). New nuclear adaptations will then arise in response to this new location of the mitogene (asterisks in Fig. 4 (2)). Although this adaptive model is possible, we have argued throughout this paper that it is a rare occurrence.

Next, we consider what happens when mitochondria- and nucleus-encoded versions of a protein have equal fitness. Near-neutral models of EGT begin with an intercompartmental gene duplication in a population [24, 50] (Fig. 4 (3)). In such models, both nuclear and mitochondrial copies are fully functional with no fitness costs associated with losing either copy [14, 95]. These studies concluded that if the nuclear copy has an equal or better fitness compared to the mitochondrial copy, mitochondria-to-nucleus transfer is inevitable. However, higher mitochondrial mutation rates were required for mitochondria-to-nucleus EGT to occur in a reasonable timeframe [14]. In these cases, the usual fates of duplications can occur [96], serving as a potential source of evolutionary innovation, via sub- or neo-functionalization [97]. A few examples of putative sub-functionalization where both nuclear and mitochondria-encoded duplicates are retained have been observed or inferred in fungi and plants (e.g. [98–103]), suggesting that these types of duplications can quickly evolve.

Finally, we consider the possibility of deleterious transfers. As discussed above, all experimental transfers come with fitness costs [64–66], and therefore, it has been suggested that all naturally occurring mitochondria-to-nucleus transfers come with initial fitness costs as well [24]. These fitness costs of transfer will vary both in time and between lineages. Thus, the transfer of a particular protein will be easier in certain lineages than in others. Such lineage-variable constraints can help explain the differences in gene migration and lineage-specific mitogenomes. But how are constraints broken in the first place, and when broken, why do constraints seem to be broken ‘all at once’?

We believe that a ‘burst-upon-drift’ (BUD) model can explain these observations. In this model, much like the neutral model described above, the starting point is an intercompartmental gene duplication in a population (Fig. 4 (3)). But in this case, the nuclear duplicate is slightly deleterious in comparison with the mitochondrial duplicate. In a large population, in which selection is strong, the mitochondrial copy would always persist. The cellular barriers will not be broken, and the nuclear copy would eventually be lost due to drift returning the system to the original state (Fig. 4 (1)). However, in a small population with high mitochondrial mutation rates, the efficiency of selection is much lower, enabling the cellular barriers to be broken by mutation pressure and drift. Thus, the mitochondrial-encoded gene will be lost more frequently due to the high mutation pressure, and the low population size will increase the chance that individuals containing only the less-fit nuclear copy will drift to fixation (Fig. 4 (4)). Once the nuclear copy is fixed upon complete transfer and the bottleneck ended, an adaptive path would resume leading to new nuclear adaptations (asterisks) and lower mitochondrial mutation rates (perhaps also arising adaptively) (Fig. 4 (5)).

Given that mitochondria-to-nucleus EGT appears to occur in spurts, current mitochondrial mutation rates may not reflect those of past events. Instead, the spurt-like nature of these EGTs may be indicative of past lineages that experienced population bottlenecks and possible concomitant increases in mitochondrial mutation rates (Fig. 4 (4)). Although mitogenomes are thought of as having much higher mutation rates than nuclear genomes [104], this is not usually the case outside of animals. For example, many plants have very low mitochondrial mutation rates, yeast mitochondrial mutation rates were grossly overestimated, and even the malaria parasite has a low mitochondrial mutation rate [105–108]. So, the spurt-like transfers and losses of mitogenes seen across the tree of life (Figs. 1 and 2) may reflect temporarily arising ‘challenging times’ for mitogenomes. For example, the ancestral population of myzozoans (which includes the malaria parasite) may have reached a bottleneck rate in which the mitochondrial mutation rate skyrocketed, which facilitated both transfer of many mitochondrial genes and severe fragmentation of its mitochondrial rRNAs. After release from the bottleneck, the population would have adapted to the new cellular realities of the nucleus-encoded mitogenes and returned to a low mitochondrial mutation rate (Fig. 4 (5)). During the bottleneck, the three requirements for transfer could be reached for many genes at the same time: (i) low selective costs, (ii) small effective population sizes, and (iii) high mitochondrial mutation rates.

## Conclusions

We set out to explain the diversity of mitogenomes across the tree of eukaryotes. By providing a schematic of mitochondrial coding capacities, we demonstrated that many lineages have encoded a nearly unchanging set of mitogenes for billions of years (Fig. 1). By consolidating mitogene contents and identifying transfer events in sequenced taxa, we showed that transfer is relatively rare, with between 1 and 16 transfers occurring per transferable mitogene (Fig. 2). With a knowledge of the diversity of mitogenomes in hand, we sought to evaluate two popular explanations for mitogene retention, the hydrophobicity and CoRR hypotheses. We concluded that while each hypothesis is consistent with available data and can explain why a subset of genes are retained in most mitogenomes, both hypotheses fail to explain why, for example, mitoribosomal proteins remain encoded in so many mitogenomes. The CoRR hypothesis and other constraints such as changes in mitogenome genetic code are good explanations for why mitogenes do not transfer in some lineages but fall short as general explanations. Thus, we turn to population genetics to explain the diversity of mitogenome coding contents.

We suggest that the apparent spurt-like evolution of mitogenome content is an indicator of ancient bottlenecks that occurred across the tree of eukaryotes. Such contractions in population sizes were most likely accompanied by drastic changes in mitochondrial mutation rates which led to the relatively rapid wholesale nuclear migration of many mitogenes, especially whenever the mitochondria were temporarily released from demanding energetic requirements [7]. We therefore contend that it is possible that the mitogenomes that we see today have largely been shaped by what we referred to here as ‘burst-upon-drift’ events and are thus the result of the contingent nature of evolution instead of being precisely honed by the slow hand of natural selection.

## Accession numbers

All sequences used in this study are publicly available from the sources specified in the Suppl. Tables N and M.

## Materials and methods

### Gene identification

We collected mitochondrial and nuclear genome and transcriptome sequences for 86 eukaryotic species from the sources specified in Additional file 2. For the identification of genes encoded in the mitogenomes, a locally installed version of MFannot software was used (https://github.com/BFL-lab/Mfannot) with the BLAST *e*-value threshold set to 1.

In another approach, we produced a set of hidden Markov models (HMMs), which subsequently served as queries in searches with HMMER v.3.3.2 (http://hmmer.org/) and predicted mitoproteins as a database. For the initial query HMM generation, protein sequences encoded in the mitogenomes of *Reclinomonas americana* and *Andalucia godoyi* were used as queries in homology searches against a database of proteins predicted in our reference dataset of mitogenomes with BLAST v.2.12.0 [109]. The hits with an *e*-value lower than 1*e* − 10 were retrieved and verified using reciprocal BLAST searches. Validated hits were aligned using MAFFT v.7.490 with the ‘linsi’ algorithm [110] and used for the initial HMM generation. Obtained HMMs were searched against predicted mitochondrial proteins, and the annotation of the hits was verified using HH-suite3 v.3.3.0 (with PDB70 and Pfam databases) and/or Swiss-Model web server [111] (https://swissmodel.expasy.org/). The hits were aligned, and a new set of HMMs was created and used for searching the homologues encoded in both mitochondrial and nuclear genomes of the species in the reference dataset. For the retrieved sequences, we obtained the first set of phylogenetic trees as described in the ‘Phylogenetic analysis’ section. Identified proteins were incorporated into the final HMMs, which were then searched against the proteins predicted in the mitochondrial and nuclear genomes of the reference species. Additionally, ORFs longer than 120 and 240 nt were predicted in the mitochondrial and nuclear genomes and transcriptomes, respectively, using the ‘getorf’ script from EMBOSS package v.6.5.7.0 [112] and used as a database for the final round of HMM-based searches. Only proteins identified in the reference dataset using MFannot and three rounds of HMM-based searches were used for constructing the final set of phylogenetic trees as described below.

Ribosomal RNA genes in mitogenomes were predicted using the RNAweasel server (https://megasun.bch.umontreal.ca/apps/rnaweasel/). Identification of tRNAs was carried out with tRNAscan v.2.0.9 [113] and Aragorn v.1.2.41 [114] in default settings.

For the identification of putative cytochrome *c* heme lyase homologs in our dataset, we have performed searches using HMMER v.3.3.2 and Pfam model PF01265 and BLAST with *Trypanosoma brucei* heme lyase (Tb927.3.3890) as queries, respectively.

### Mitochondrial pre-sequence prediction

Mitochondrial pre-sequences were predicted using MitoFates v.1.1 with ‘fungi’, ‘metazoa’, and ‘plant’ options [115]; TargetP v.2.0 with the ‘-org’ option set to ‘non-pl’ and ‘pl’ [116]; and TPpred3 with the ‘-k N’ option [117]. A mitochondrial pre-sequence was considered valid if it was inferred by at least two out of three tools.

### Phylogenetic analysis

The set of HMMs described above was additionally used for searches in the following datasets: (a) the reference set of 102 bacterial genomes (Additional File 3), (b) a set of proteins encoded in the protist mitogenomes from NCBI RefSeq organelle genome database (download date: 26.10.2022), and (c) ‘The Comparative Set’ from EukProt v.3 (for HMMs representing the electron transport chain components) [118]. Retrieved homologues were unified with the hits obtained as described in the ‘Gene identification’ section, and the identical sequences were filtered out from the final dataset using CD-HIT v. 4.8.1 with the ‘-c 1’ option [119]. Protein sequences were aligned using MAFFT v.7.490 with the ‘linsi’ algorithm [110], and the alignments were trimmed using trimAl v.1.4.rev15 with the ‘-gt 0.8’ option [120] and ClipKIT v. with the default settings [121]. Maximum-likelihood phylogenetic trees were inferred using IQ-TREE 2 [122] with automatically selected models specified for each protein in Additional file 4 and 1000 ultrafast bootstrap replicates. We could not produce a reasonably resolved tree for the ribosomal protein 36 due to its inadequate sequence length (~ 50 amino acids on average). Of note, we also tried to use the GHOST model for several proteins within our dataset, but it did not result in a significant increase in branch support values. Very short (partial) sequences (containing more than 60% of gaps in the original alignment) and very divergent sequences (forming very long branches in the initial phylogenetic trees) were excluded from the final phylogenetic analysis. Annotations of the proteins in the final dataset were additionally confirmed using HH-suite3 v.3.3.0 (with PDB70 and Pfam databases) and/or the Swiss-Model web server.

### Supplementary Information

**Additional file 1.** Dendrogram obtained using hierarchical cluster analysis based on the patterns of mitochondrial gene retention, loss, and transfer to the nuclear genome. The species with no nuclear genomic/transcriptomic data available were excluded from the analysis. The species names are coloured according to the affiliation to a particular eukaryotic group on the cladogram on the left.

**Additional file 2.** Patterns of gene distribution across mitochondrial and nuclear genomes of the reference species.

**Additional file 3.** Bacterial reference dataset used in this study.

**Additional file 4.** Maximum likelihood phylogenetic trees inferred using IQ-TREE 2. Proteins encoded in mitogenomes are highlighted in violet; putative cases of mitochondria-to-nucleus gene transfers for the species in our dataset are shown in green. Numbers in brackets indicate the number of sequences within the collapsed clades. Amino acid substitution models for each protein were automatically selected in IQ-TREE 2 and are given below each tree. Software used for the alignment trimming is also indicated. Species abbreviations are as in Additional file 2. Numbers at the branches represent ultrafast bootstrap supports; only values above 75 are shown. Scale bar indicates the number of substitutions per site.

## Data Availability

All data obtained are available as additional files.

## Abbreviations

BUD: Burst-upon-drift
CCHL: Cytochrome *c* heme lyase
ccm: Cytochrome *c* maturation
CoRR: Co-location for redox regulation
EGT: Endosymbiont gene transfer
ETC: Electron transport chain
LECA: Last eukaryotic common ancestor
mitogenes: Mitochondria-encoded genes
mitogenomes: Mitochondrial genomes
mitoproteins: Mitochondrial proteins
mitoribosomal: Mitochondrial ribosomal
MTS: Mitochondrial targeting signal
ROS: Reactive oxygen species
