# Supplementary figures and images for: Mitochondrial genomes revisited: why do different lineages retain different genes?
Source: BMC Biol. 2024 Jan 25;22:15. doi: 10.1186/s12915-024-01824-1 (PMC10809612; doi:10.1186/s12915-024-01824-1)

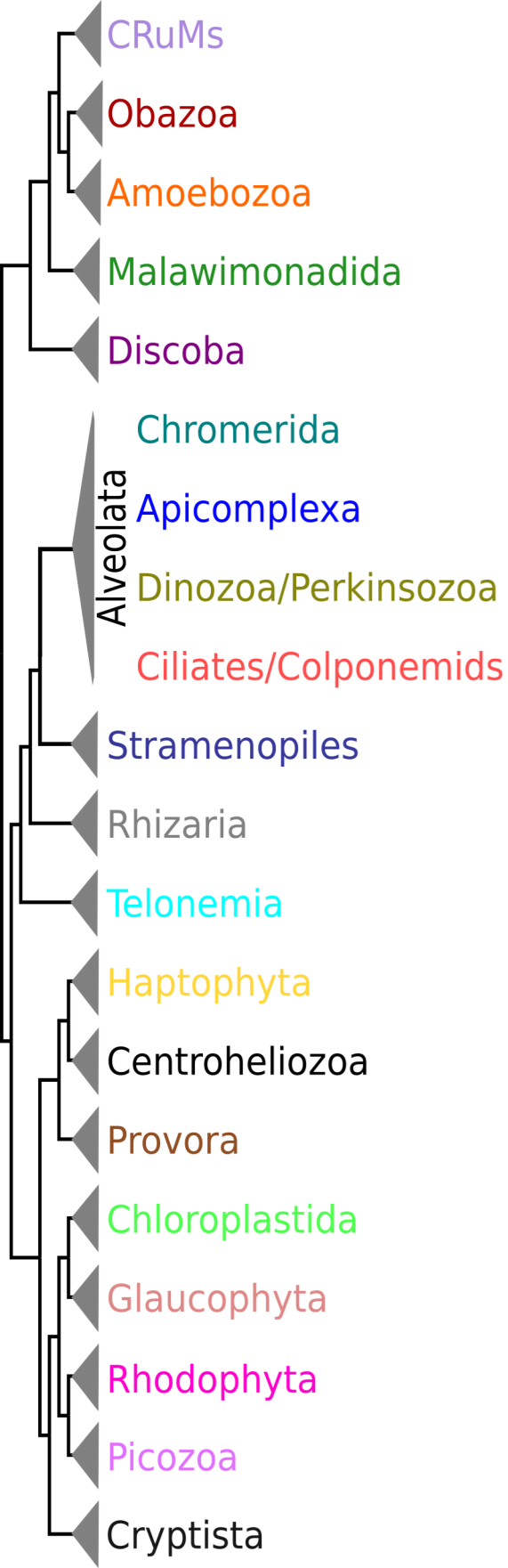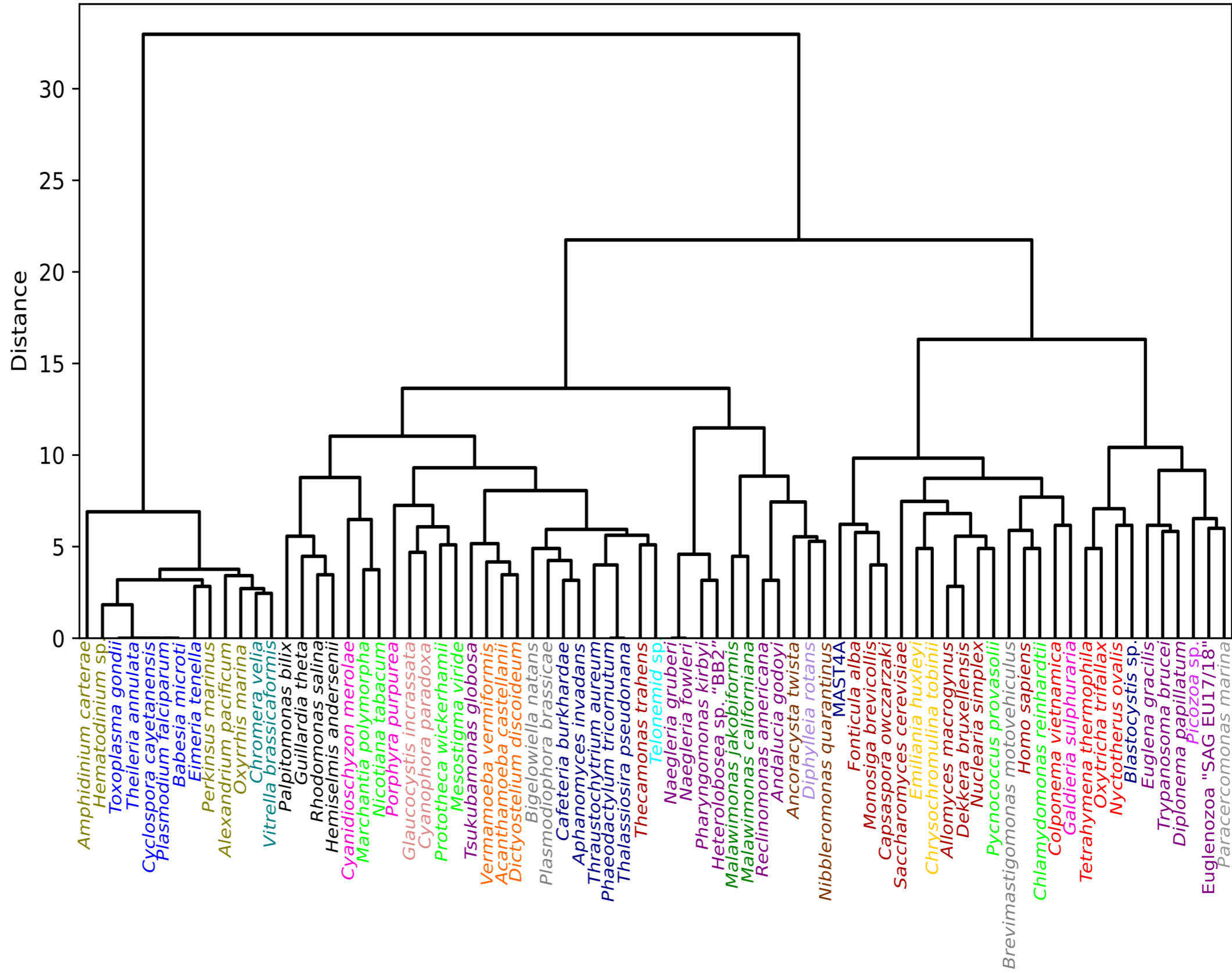

Supplement: Supplementary file 1 — Additional file 1. Dendrogram obtained using hierarchical cluster analysis based on the patterns of mitochondrial gene retention, loss, and transfer to the nuclear genome. The species with no nuclear genomic/transcriptomic data available were excluded from the analysis. The species names are coloured according to the affiliation to a particular eukaryotic group on the cladogram on the left. [file 12915_2024_1824_MOESM1_ESM.pdf]
