# Supplementary material for: Mitochondrial genomes revisited: why do different lineages retain different genes?
Source: BMC Biol. 2024 Jan 25;22:15. doi: 10.1186/s12915-024-01824-1 (PMC10809612; doi:10.1186/s12915-024-01824-1)
Supplement: Supplementary file 4 — Additional file 4. Maximum likelihood phylogenetic trees inferred using IQ-TREE 2. Proteins encoded in mitogenomes are highlighted in violet; putative cases of mitochondria-to-nucleus gene transfers for the species in our dataset are shown in green. Numbers in brackets indicate the number of sequences within the collapsed clades. Amino acid substitution models for each protein were automatically selected in IQ-TREE 2 and are given below each tree. Software used for the alignment trimming is also indicated. Species abbreviations are as in Additional file 2. Numbers at the branches represent ultrafast bootstrap supports; only values above 75 are shown. Scale bar indicates the number of substitutions per site. [file 12915_2024_1824_MOESM4_ESM.pdf]

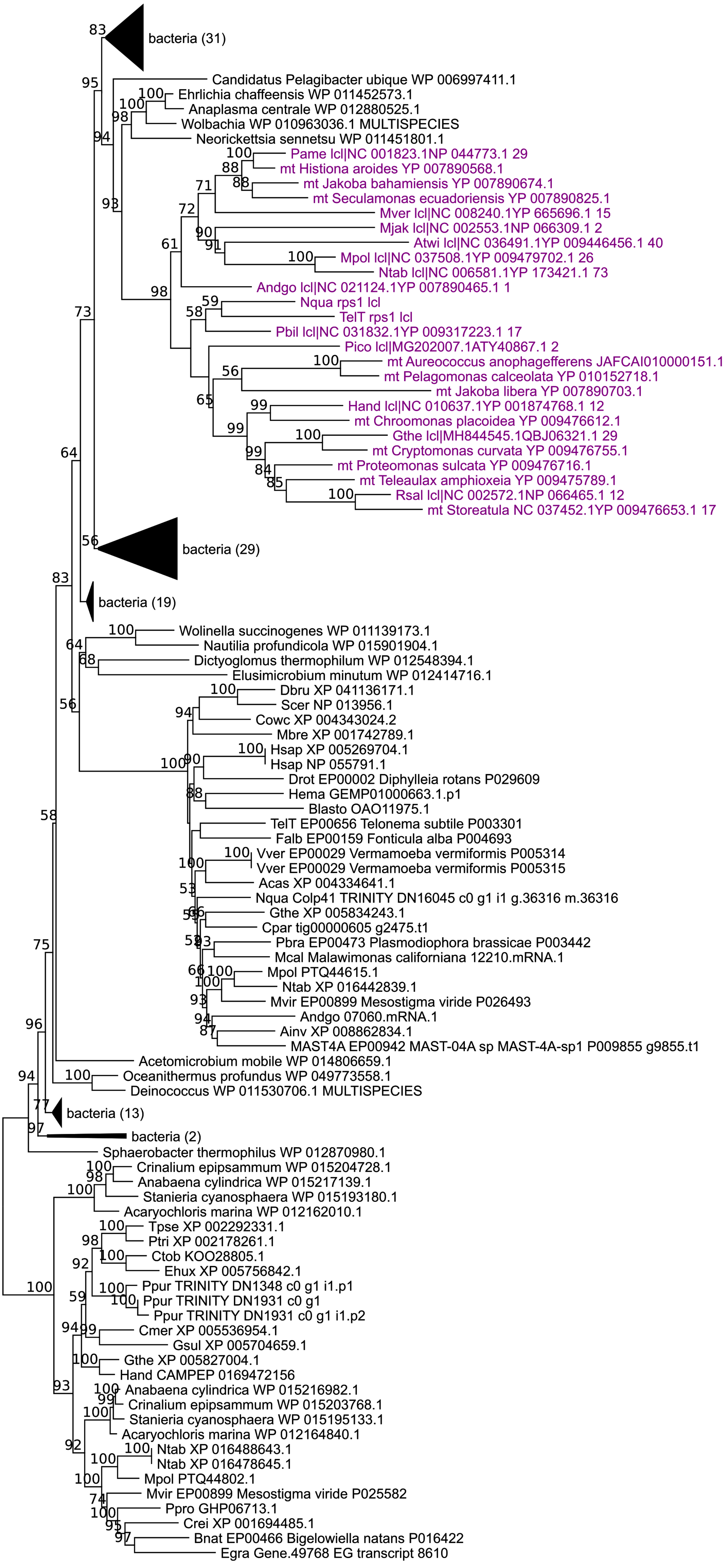

Protein: **rps1**; alignment timming: ClipKIT; IQ-TREE2 best-fit model: LG+R7

0.50

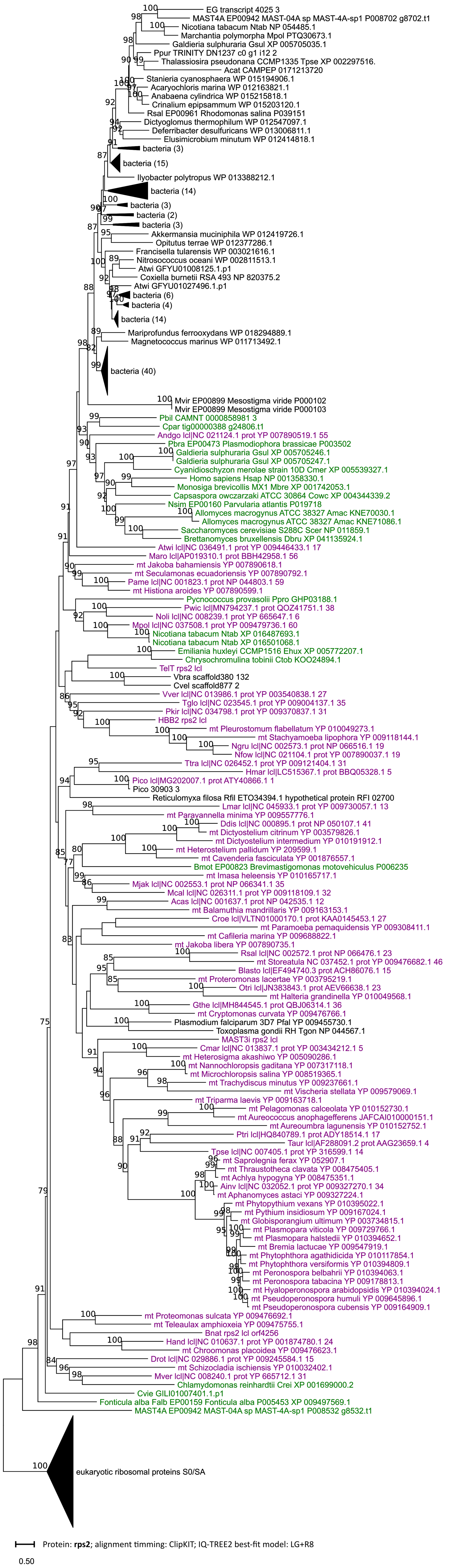

EG transcript 4025 3
MAST4A EP00942 MAST-04A sp MAST-4A-sp1 P008702 g8702.t1
Nicotiana tabacum Ntab NP 054485.1
Marchantia polymorpha Mpol PTQ30673.1
Galdieria sulphuraria Gsul XP 005705035.1
Ppur TRINITY DN1237 c0 g1 i12 2
Thalassiosira pseudonana CCMP1335 Tpse XP 002297516.
Acat CAMPEP 0171213720
Stanieria cyanosphaera WP 015194906.1
Acaryochloris marina WP 012163821.1
Anabaena cylindrica WP 015215818.1
Crinalium epipsammum WP 015203120.1
Rsal EP00961 Rhodomonas salina P039151
Dictyoglomus thermophilum WP 012547097.1
Deferribacter desulfuricans WP 013006811.1
Elusimicrobium minutum WP 012414818.1
bacteria (3)
bacteria (15)
Ilyobacter polytropus WP 013388212.1
bacteria (14)
bacteria (3)
bacteria (2)
bacteria (3)
Akkermansia muciniphila WP 012419726.1
Opitutus terrae WP 012377286.1
Francisella tularensis WP 003021616.1
Nitrosococcus oceani WP 002811513.1
Atwi GFYU01008125.1.p1
Coxiella burnetii RSA 493 NP 820375.2
Atwi GFYU01027496.1.p1
bacteria (6)
bacteria (4)
bacteria (14)
Mariprofundus ferrooxydans WP 018294889.1
Magnetococcus marinus WP 011713492.1
bacteria (40)
Mvir EP00899 Mesostigma viride P000102
Mvir EP00899 Mesostigma viride P000103
Pbil CAMNT 0000858981 3
Cpar tig00000388 g24806.t1
Andgo lcl|NC 021124.1 prot YP 007890519.1 55
Pbra EP00473 Plasmodiophora brassicae P003502
Galdieria sulphuraria Gsul XP 005705246.1
Galdieria sulphuraria Gsul XP 005705247.1
Cyanidioschyzon merolae strain 10D Cmer XP 005539327.1
Homo sapiens Hsap NP 001358330.1
Monosiga brevicollis MX1 Mbre XP 001742053.1
Capsaspora owczarzaki ATCC 30864 Cowc XP 004344339.2
Nsim EP00160 Parvularia atlantis P019718
Allomyces macrogynus ATCC 38327 Amac KNE70030.1
Allomyces macrogynus ATCC 38327 Amac KNE71086.1
Saccharomyces cerevisiae S288C Scer NP 011859.1
Brettanomyces bruxellensis Dbru XP 041135924.1
Atwi lcl|NC 036491.1 prot YP 009446433.1 17
Maro lcl|AP019310.1 prot BBH42958.1 56
mt Jakoba bahamiensis YP 007890618.1
mt Seculamonas ecuadoriensis YP 007890792.1
Pame lcl|NC 001823.1 prot NP 044803.1 59
mt Histiona aroides YP 007890599.1
Pycnococcus provasolii Ppro GHP03188.1
Pwic lcl|MN794237.1 prot QOZ41751.1 38
Noli lcl|NC 008239.1 prot YP 665647.1 6
Mpol lcl|NC 037508.1 prot YP 009479736.1 60
Nicotiana tabacum Ntab XP 016487693.1
Nicotiana tabacum Ntab XP 016501068.1
Emiliania huxleyi CCMP1516 Ehux XP 005772207.1
Chrysochromulina tobinii Ctob KOO24894.1
TelT rps2 lcl
Vbra scaffold380 132
Cvel scaffold877 2
Vver lcl|NC 013986.1 prot YP 003540838.1 27
Tglo lcl|NC 023545.1 prot YP 009004137.1 35
Pkir lcl|NC 034798.1 prot YP 009370837.1 31
HBB2 rps2 lcl
mt Pleurostomum flabellatum YP 010049273.1
mt Stachyamoeba lipophora YP 009118144.1
Ngru lcl|NC 002573.1 prot NP 066516.1 19
Nfow lcl|NC 021104.1 prot YP 007890037.1 19
Ttra lcl|NC 026452.1 prot YP 009121404.1 31
Hmar lcl|LC515367.1 prot BBQ05328.1 5
Pico lcl|MG202007.1 prot ATY40866.1 1
Pico 30903 3
Reticulomyxa filosa Rfil ETO34394.1 hypothetical protein RFI 02700
Lmar lcl|NC 045933.1 prot YP 009730057.1 13
mt Paravannella minima YP 009557776.1
Ddis lcl|NC 000895.1 prot NP 050107.1 41
mt Dictyostelium citrinum YP 003579826.1
mt Dictyostelium intermedium YP 010191912.1
mt Heterostelium pallidum YP 209599.1
mt Cavenderia fasciculata YP 001876557.1
Bmot EP00823 Brevimastigomonas motovehiculus P006235
mt Imasa heleensis YP 010165717.1
Mjak lcl|NC 002553.1 prot NP 066341.1 35
Mcal lcl|NC 026311.1 prot YP 009118109.1 32
Acas lcl|NC 001637.1 prot NP 042535.1 12
mt Balamuthia mandrillaris YP 009163153.1
Croe lcl|VLTN01000170.1 prot KAA0145453.1 27
mt Paramoeba pemaquidensis YP 009308411.1
mt Cafileria marina YP 009688822.1
mt Jakoba libera YP 007890735.1
Rsal lcl|NC 002572.1 prot NP 066476.1 23
mt Storeatula NC 037452.1 prot YP 009476682.1 46
Blasto lcl|EF494740.3 prot ACH86076.1 15
mt Proteromonas lacertae YP 003795219.1
Otri lcl|JN383843.1 prot AEV66638.1 23
mt Halteria grandinella YP 010049568.1
Gthe lcl|MH844545.1 prot QBJ06314.1 36
mt Cryptomonas curvata YP 009476766.1
Plasmodium falciparum 3D7 Pfal YP 009455730.1
Toxoplasma gondii RH Tgon NP 044567.1
MAST3i rps2 lcl
Cmar lcl|NC 013837.1 prot YP 003434212.1 5
mt Heterosigma akashiwo YP 005090286.1
mt Nannochloropsis gaditana YP 007317118.1
mt Microchloropsis salina YP 008519365.1
mt Trachydiscus minutus YP 009237661.1
mt Vischeria stellata YP 009579069.1
mt Triparma laevis YP 009163718.1
mt Pelagomonas calceolata YP 010152730.1
mt Aureococcus anophagefferens JAFCAI010000151.1
mt Aureoumbra lagunensis YP 010152752.1
Ptri lcl|HQ840789.1 prot ADY18514.1 17
Taur lcl|AF288091.2 prot AAG23659.1 4
Tpse lcl|NC 007405.1 prot YP 316599.1 14
mt Saprolegnia ferax YP 052907.1
mt Thraustotheca clavata YP 008475405.1
mt Achlya hypogyna YP 008475351.1
Ainv lcl|NC 032052.1 prot YP 009327270.1 34
mt Aphanomyces astaci YP 009327224.1
mt Phytopythium vexans YP 010395022.1
mt Pythium insidiosum YP 009167024.1
mt Globisporangium ultimum YP 003734815.1
mt Plasmopara viticola YP 009729766.1
mt Plasmopara halstedii YP 010394652.1
mt Bremia lactucae YP 009547919.1
mt Phytophthora agathidicida YP 010117854.1
mt Phytophthora versiformis YP 010394809.1
mt Peronospora belbahrii YP 010394063.1
mt Peronospora tabacina YP 009178813.1
mt Hyaloperonospora arabidopsidis YP 010394024.1
mt Pseudoperonospora humuli YP 009645896.1
mt Pseudoperonospora cubensis YP 009164909.1
mt Proteomonas sulcata YP 009476692.1
mt Teleaulax amphioxeia YP 009475755.1
Bnat rps2 lcl orf4256
Hand lcl|NC 010637.1 prot YP 001874780.1 24
mt Chroomonas placoidea YP 009476623.1
Drot lcl|NC 029886.1 prot YP 009245584.1 15
mt Schizocladia ischiensis YP 010032402.1
Mver lcl|NC 008240.1 prot YP 665712.1 31
Chlamydomonas reinhardtii Crei XP 001699000.2
Cvie GILI01007401.1.p1
Fonticula alba Falb EP00159 Fonticula alba P005453 XP 009497569.1
MAST4A EP00942 MAST-04A sp MAST-4A-sp1 P008532 g8532.t1
eukaryotic ribosomal proteins S0/SA
Protein: rps2; alignment timming: ClipKIT; IQ-TREE2 best-fit model: LG+R8
0.50

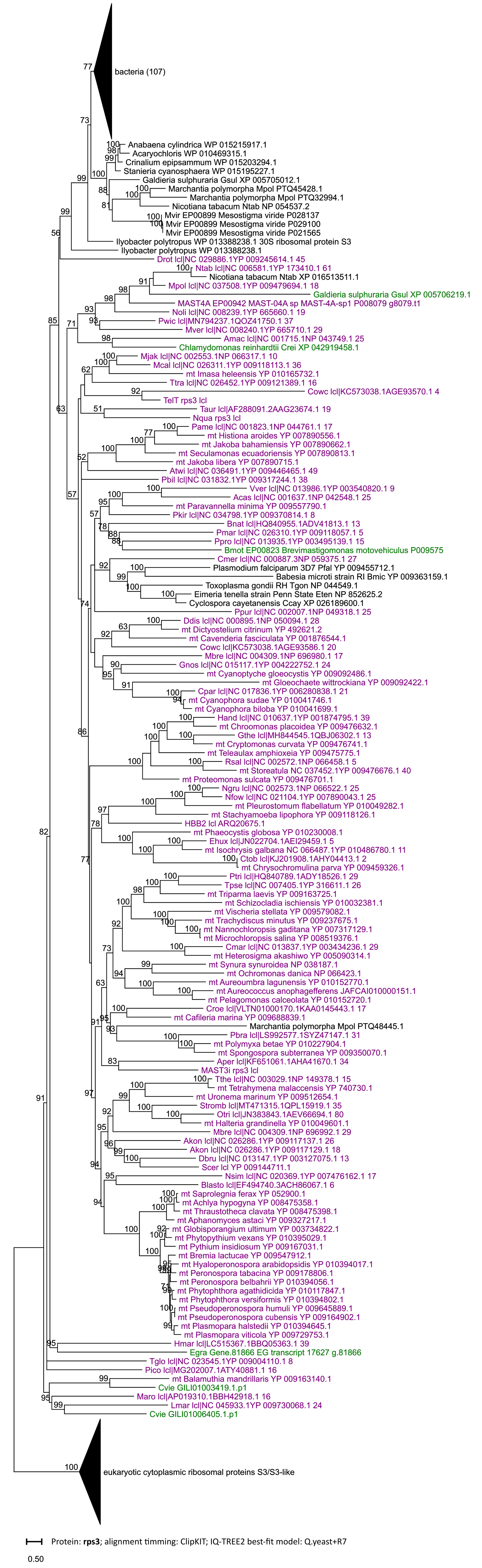
bacteria (107)
Anabaena cylindrica WP 015215917.1
Acaryochloris WP 010469315.1
Crinalium epipsammum WP 015203294.1
Stanieria cyanosphaera WP 015195227.1
Galdieria sulphuraria Gsul XP 005705012.1
Marchantia polymorpha Mpol PTQ45428.1
Marchantia polymorpha Mpol PTQ32994.1
Nicotiana tabacum Ntab NP 054537.2
Mvir EP00899 Mesostigma viride P028137
Mvir EP00899 Mesostigma viride P029100
Mvir EP00899 Mesostigma viride P021565
Ilyobacter polytropus WP 013388238.1 30S ribosomal protein S3
Ilyobacter polytropus WP 013388238.1
Drot lcl|NC 029886.1YP 009245614.1 45
Ntab lcl|NC 006581.1YP 173410.1 61
Nicotiana tabacum Ntab XP 016513511.1
Mpol lcl|NC 037508.1YP 009479694.1 18
Galdieria sulphuraria Gsul XP 005706219.1
MAST4A EP00942 MAST-04A sp MAST-4A-sp1 P008079 g8079.t1
Noli lcl|NC 008239.1YP 665660.1 19
Pwic lcl|MN794237.1QOZ41750.1 37
Mver lcl|NC 008240.1YP 665710.1 29
Amac lcl|NC 001715.1NP 043749.1 25
Chlamydomonas reinhardtii Crei XP 042919458.1
Mjak lcl|NC 002553.1NP 066317.1 10
Mcal lcl|NC 026311.1YP 009118113.1 36
mt Imasa heleensis YP 010165732.1
Ttra lcl|NC 026452.1YP 009121389.1 16
Cowc lcl|KC573038.1AGE93570.1 4
TelT rps3 lcl
Taur lcl|AF288091.2AAG23674.1 19
Nqua rps3 lcl
Pame lcl|NC 001823.1NP 044761.1 17
mt Histiona aroides YP 007890556.1
mt Jakoba bahamiensis YP 007890662.1
mt Seculamonas ecuadoriensis YP 007890813.1
mt Jakoba libera YP 007890715.1
Atwi lcl|NC 036491.1YP 009446465.1 49
Pbil lcl|NC 031832.1YP 009317244.1 38
Vver lcl|NC 013986.1YP 003540820.1 9
Acas lcl|NC 001637.1NP 042548.1 25
mt Paravannella minima YP 009557790.1
Pkir lcl|NC 034798.1YP 009370814.1 8
Bnat lcl|HQ840955.1ADV41813.1 13
Pmar lcl|NC 026310.1YP 009118057.1 5
Ppro lcl|NC 013935.1YP 003495139.1 15
Bmot EP00823 Brevimastigomonas motovehiculus P009575
Cmer lcl|NC 000887.3NP 059375.1 27
Plasmodium falciparum 3D7 Pfal YP 009455712.1
Babesia microti strain RI Bmic YP 009363159.1
Toxoplasma gondii RH Tgon NP 044549.1
Eimeria tenella strain Penn State Eten NP 852625.2
Cyclospora cayetanensis Ccay XP 026189600.1
Ppur lcl|NC 002007.1NP 049318.1 25
Ddis lcl|NC 000895.1NP 050094.1 28
mt Dictyostelium citrinum YP 492621.2
mt Cavenderia fasciculata YP 001876544.1
Cowc lcl|KC573038.1AGE93586.1 20
Mbre lcl|NC 004309.1NP 696980.1 17
Gnos lcl|NC 015117.1YP 004222752.1 24
mt Cyanoptyche gloeocystis YP 009092486.1
mt Gloeochaete wittrockiana YP 009092422.1
Cpar lcl|NC 017836.1YP 006280838.1 21
mt Cyanophora sudae YP 010041746.1
mt Cyanophora biloba YP 010041699.1
Hand lcl|NC 010637.1YP 001874795.1 39
mt Chroomonas placoidea YP 009476632.1
Gthe lcl|MH844545.1QBJ06302.1 13
mt Cryptomonas curvata YP 009476741.1
mt Teleaulax amphioxeia YP 009475775.1
Rsal lcl|NC 002572.1NP 066458.1 5
mt Storeatula NC 037452.1YP 009476676.1 40
mt Proteomonas sulcata YP 009476701.1
Ngru lcl|NC 002573.1NP 066522.1 25
Nfow lcl|NC 021104.1YP 007890043.1 25
mt Pleurostomum flabellatum YP 010049282.1
mt Stachyamoeba lipophora YP 009118126.1
HBB2 lcl ARQ20675.1
mt Phaeocystis globosa YP 010230008.1
Ehux lcl|JN022704.1AEI29459.1 5
mt Isochrysis galbana NC 066487.1YP 010486780.1 11
Ctob lcl|KJ201908.1AHY04413.1 2
mt Chrysochromulina parva YP 009459326.1
Ptri lcl|HQ840789.1ADY18526.1 29
Tpse lcl|NC 007405.1YP 316611.1 26
mt Triparma laevis YP 009163725.1
mt Schizocladia ischiensis YP 010032381.1
mt Vischeria stellata YP 009579082.1
mt Trachydiscus minutus YP 009237675.1
mt Nannochloropsis gaditana YP 007317129.1
mt Microchloropsis salina YP 008519376.1
Cmar lcl|NC 013837.1YP 003434236.1 29
mt Heterosigma akashiwo YP 005090314.1
mt Synura synuroidea NP 038187.1
mt Ochromonas danica NP 066423.1
mt Aureoumbra lagunensis YP 010152770.1
mt Aureococcus anophagefferens JAFCAI010000151.1
mt Pelagomonas calceolata YP 010152720.1
Croe lcl|VLTN01000170.1KAA0145443.1 17
mt Cafileria marina YP 009688839.1
Marchantia polymorpha Mpol PTQ48445.1
Pbra lcl|LS992577.1SYZ47147.1 31
mt Polymyxa betae YP 010227904.1
mt Spongospora subterranea YP 009350070.1
Aper lcl|KF651061.1AHA41670.1 34
MAST3i rps3 lcl
Tthe lcl|NC 003029.1NP 149378.1 15
mt Tetrahymena malaccensis YP 740730.1
mt Uronema marinum YP 009512654.1
Stromb lcl|MT471315.1QPL15919.1 35
Otri lcl|JN383843.1AEV66694.1 80
mt Halteria grandinella YP 010049601.1
Mbre lcl|NC 004309.1NP 696992.1 29
Akon lcl|NC 026286.1YP 009117137.1 26
Akon lcl|NC 026286.1YP 009117129.1 18
Dbru lcl|NC 013147.1YP 003127075.1 13
Scer lcl YP 009144711.1
Nsim lcl|NC 020369.1YP 007476162.1 17
Blasto lcl|EF494740.3ACH86067.1 6
mt Saprolegnia ferax YP 052900.1
mt Achlya hypogyna YP 008475358.1
mt Thraustotheca clavata YP 008475398.1
mt Aphanomyces astaci YP 009327217.1
mt Globisporangium ultimum YP 003734822.1
mt Phytopythium vexans YP 010395029.1
mt Pythium insidiosum YP 009167031.1
mt Bremia lactucae YP 009547912.1
mt Hyaloperonospora arabidopsidis YP 010394017.1
mt Peronospora tabacina YP 009178806.1
mt Peronospora belbahrii YP 010394056.1
mt Phytophthora agathidicida YP 010117847.1
mt Phytophthora versiformis YP 010394802.1
mt Pseudoperonospora humuli YP 009645889.1
mt Pseudoperonospora cubensis YP 009164902.1
mt Plasmopara halstedii YP 010394645.1
mt Plasmopara viticola YP 009729753.1
Hmar lcl|LC515367.1BBQ05363.1 39
Egra Gene.81866 EG transcript 17627 g.81866
Tglo lcl|NC 023545.1YP 009004110.1 8
Pico lcl|MG202007.1ATY40881.1 16
mt Balamuthia mandrillaris YP 009163140.1
Cvie GILI01003419.1.p1
Maro lcl|AP019310.1BBH42918.1 16
Lmar lcl|NC 045933.1YP 009730068.1 24
Cvie GILI01006405.1.p1
eukaryotic cytoplasmic ribosomal proteins S3/S3-like
0.50
Protein: rps3; alignment timming: ClipKIT; IQ-TREE2 best-fit model: Q.yeast+R7

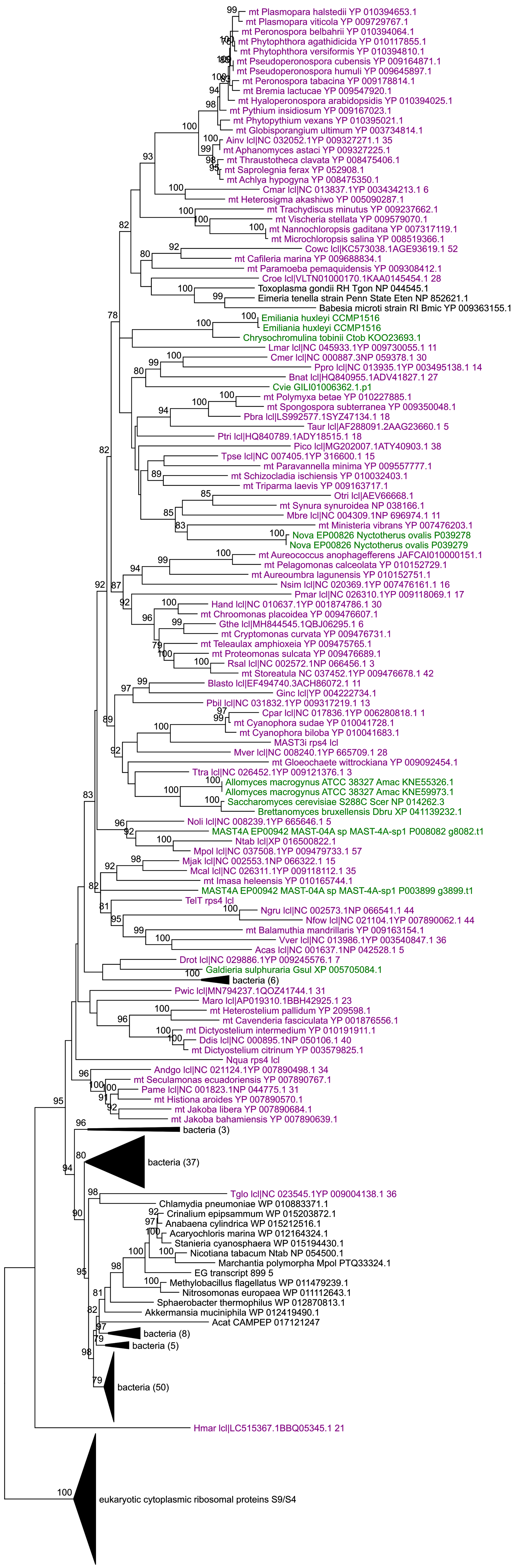

Protein: **rps4**; alignment timming: trimAl; IQ-TREE2 best-fit model: Q.yeast+R7

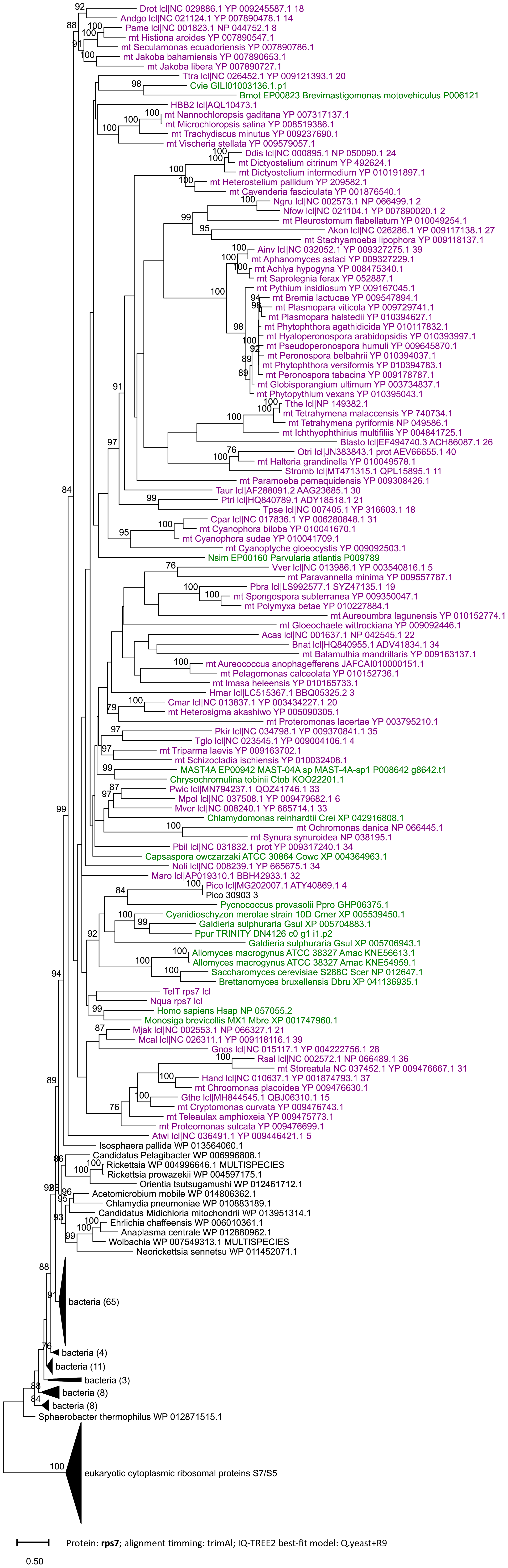

Protein: **rps7**; alignment timming: trimAl; IQ-TREE2 best-fit model: Q.yeast+R9

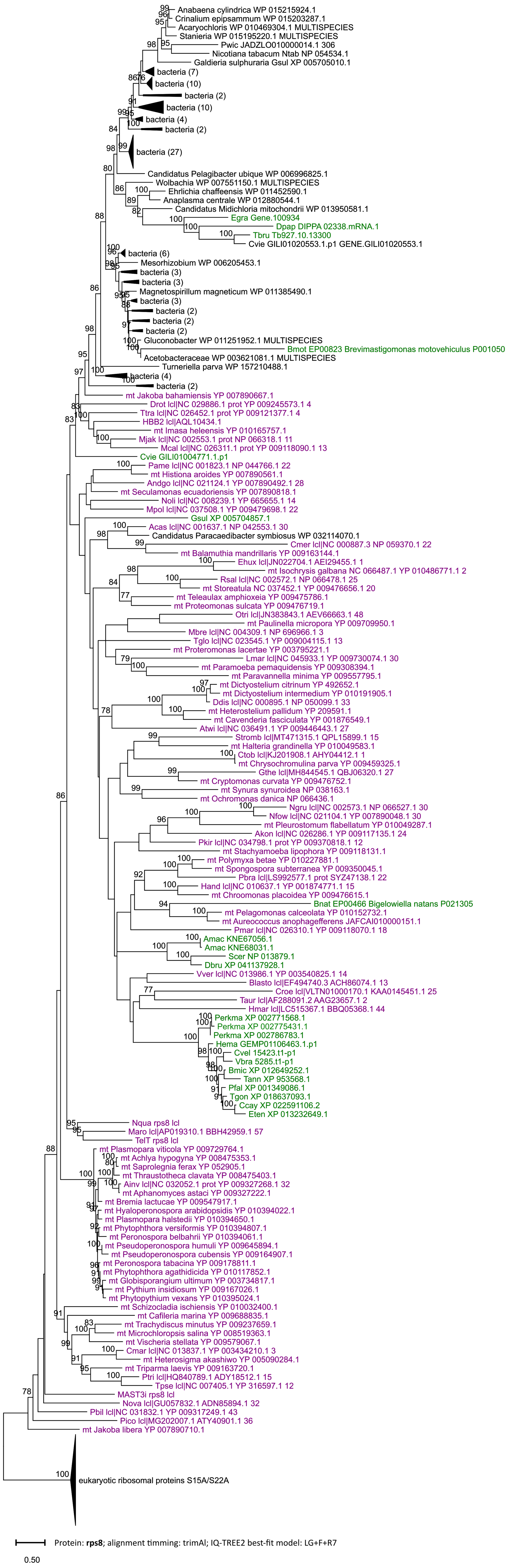
Anabaena cylindrica WP 015215924.1
Crinalium epipsammum WP 015203287.1
Acaryochloris WP 010469304.1 MULTISPECIES
Stanieria WP 015195220.1 MULTISPECIES
Pwic JADZLO010000014.1 306
Nicotiana tabacum Ntab NP 054534.1
Galdieria sulphuraria Gsul XP 005705010.1
bacteria (7)
bacteria (10)
bacteria (2)
bacteria (10)
bacteria (4)
bacteria (2)
bacteria (27)
Candidatus Pelagibacter ubique WP 006996825.1
Wolbachia WP 007551150.1 MULTISPECIES
Ehrlichia chaffeensis WP 011452590.1
Anaplasma centrale WP 012880544.1
Candidatus Midichloria mitochondrii WP 013950581.1
Egra Gene.100934
Dpap DIPPA 02338.mRNA.1
Tbru Tb927.10.13300
Cvie GILI01020553.1.p1 GENE.GILI01020553.1
bacteria (6)
Mesorhizobium WP 006205453.1
bacteria (3)
bacteria (3)
Magnetospirillum magneticum WP 011385490.1
bacteria (3)
bacteria (2)
bacteria (2)
bacteria (2)
Gluconobacter WP 011251952.1 MULTISPECIES
Bmot EP00823 Brevimastigomonas motovehiculus P001050
Acetobacteraceae WP 003621081.1 MULTISPECIES
Turneriella parva WP 157210488.1
bacteria (4)
bacteria (2)
mt Jakoba bahamiensis YP 007890667.1
Drot lcl|NC 029886.1 prot YP 009245573.1 4
Ttra lcl|NC 026452.1 prot YP 009121377.1 4
HBB2 lcl|AQL10434.1
mt Imasa heleensis YP 010165757.1
Mjak lcl|NC 002553.1 prot NP 066318.1 11
Mcal lcl|NC 026311.1 prot YP 009118090.1 13
Cvie GILI01004771.1.p1
Pame lcl|NC 001823.1 NP 044766.1 22
mt Histiona aroides YP 007890561.1
Andgo lcl|NC 021124.1 YP 007890492.1 28
mt Seculamonas ecuadoriensis YP 007890818.1
Noli lcl|NC 008239.1 YP 665655.1 14
Mpol lcl|NC 037508.1 YP 009479698.1 22
Gsul XP 005704857.1
Acas lcl|NC 001637.1 NP 042553.1 30
Candidatus Paracaedibacter symbiosus WP 032114070.1
Cmer lcl|NC 000887.3 NP 059370.1 22
mt Balamuthia mandrillaris YP 009163144.1
Ehux lcl|JN022704.1 AEI29455.1 1
mt Isochrysis galbana NC 066487.1 YP 010486771.1 2
Rsal lcl|NC 002572.1 NP 066478.1 25
mt Storeatula NC 037452.1 YP 009476656.1 20
mt Teleaulax amphioxeia YP 009475786.1
mt Proteomonas sulcata YP 009476719.1
Otri lcl|JN383843.1 AEV66663.1 48
mt Paulinella micropora YP 009709950.1
Mbre lcl|NC 004309.1 NP 696966.1 3
Tglo lcl|NC 023545.1 YP 009004115.1 13
mt Proteromonas lacertae YP 003795221.1
Lmar lcl|NC 045933.1 YP 009730074.1 30
mt Paramoeba pemaquidensis YP 009308394.1
mt Paravannella minima YP 009557795.1
mt Dictyostelium citrinum YP 492652.1
mt Dictyostelium intermedium YP 010191905.1
Ddis lcl|NC 000895.1 NP 050099.1 33
mt Heterostelium pallidum YP 209591.1
mt Cavenderia fasciculata YP 001876549.1
Atwi lcl|NC 036491.1 YP 009446443.1 27
Stromb lcl|MT471315.1 QPL15899.1 15
mt Halteria grandinella YP 010049583.1
Ctob lcl|KJ201908.1 AHY04412.1 1
mt Chrysochromulina parva YP 009459325.1
Gthe lcl|MH844545.1 QBJ06320.1 27
mt Cryptomonas curvata YP 009476752.1
mt Synura synuroidea NP 038163.1
mt Ochromonas danica NP 066436.1
Ngru lcl|NC 002573.1 NP 066527.1 30
Nfow lcl|NC 021104.1 YP 007890048.1 30
mt Pleurostomum flabellatum YP 010049287.1
Akon lcl|NC 026286.1 YP 009117135.1 24
Pkir lcl|NC 034798.1 prot YP 009370818.1 12
mt Stachyamoeba lipophora YP 009118131.1
mt Polymyxa betae YP 010227881.1
mt Spongospora subterranea YP 009350045.1
Pbra lcl|LS992577.1 prot SYZ47138.1 22
Hand lcl|NC 010637.1 YP 001874771.1 15
mt Chroomonas placoidea YP 009476615.1
Bnat EP00466 Bigelowiella natans P021305
mt Pelagomonas calceolata YP 010152732.1
mt Aureococcus anophagefferens JAFCAI010000151.1
Pmar lcl|NC 026310.1 YP 009118070.1 18
Amac KNE67056.1
Amac KNE68031.1
Scer NP 013879.1
Dbru XP 041137928.1
Vver lcl|NC 013986.1 YP 003540825.1 14
Blasto lcl|EF494740.3 ACH86074.1 13
Croe lcl|VLTN01000170.1 KAA0145451.1 25
Taur lcl|AF288091.2 AAG23657.1 2
Hmar lcl|LC515367.1 BBQ05368.1 44
Perkma XP 002771568.1
Perkma XP 002775431.1
Perkma XP 002786783.1
Hema GEMP01106463.1.p1
Cvel 15423.t1-p1
Vbra 5285.t1-p1
Bmic XP 012649252.1
Tann XP 953568.1
Pfal XP 001349086.1
Tgon XP 018637093.1
Ccay XP 022591106.2
Eten XP 013232649.1
Nqua rps8 lcl
Maro lcl|AP019310.1 BBH42959.1 57
TelT rps8 lcl
mt Plasmopara viticola YP 009729764.1
mt Achlya hypogyna YP 008475353.1
mt Saprolegnia ferax YP 052905.1
mt Thraustotheca clavata YP 008475403.1
Ainv lcl|NC 032052.1 prot YP 009327268.1 32
mt Aphanomyces astaci YP 009327222.1
mt Bremia lactucae YP 009547917.1
mt Hyaloperonospora arabidopsidis YP 010394022.1
mt Plasmopara halstedii YP 010394650.1
mt Phytophthora versiformis YP 010394807.1
mt Peronospora belbahrii YP 010394061.1
mt Pseudoperonospora humuli YP 009645894.1
mt Pseudoperonospora cubensis YP 009164907.1
mt Peronospora tabacina YP 009178811.1
mt Phytophthora agathidicida YP 010117852.1
mt Globisporangium ultimum YP 003734817.1
mt Pythium insidiosum YP 009167026.1
mt Phytopythium vexans YP 010395024.1
mt Schizocladia ischiensis YP 010032400.1
mt Cafileria marina YP 009688835.1
mt Trachydiscus minutus YP 009237659.1
mt Microchloropsis salina YP 008519363.1
mt Vischeria stellata YP 009579067.1
Cmar lcl|NC 013837.1 YP 003434210.1 3
mt Heterosigma akashiwo YP 005090284.1
mt Triparma laevis YP 009163720.1
Ptri lcl|HQ840789.1 ADY18512.1 15
Tpse lcl|NC 007405.1 YP 316597.1 12
MAST3i rps8 lcl
Nova lcl|GU057832.1 ADN85894.1 32
Pbil lcl|NC 031832.1 YP 009317249.1 43
Pico lcl|MG202007.1 ATY40901.1 36
mt Jakoba libera YP 007890710.1
eukaryotic ribosomal proteins S15A/S22A
0.50
Protein: rps8; alignment timming: trimAl; IQ-TREE2 best-fit model: LG+F+R7

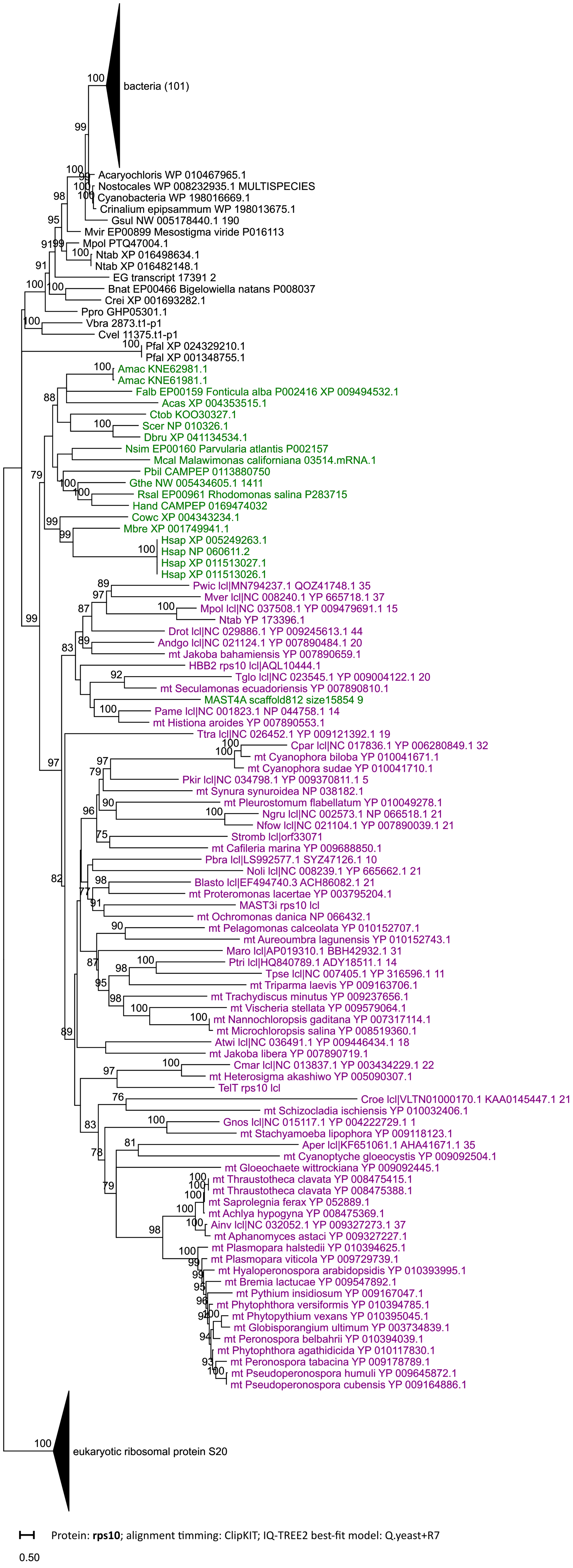

Protein: **rps10**; alignment timming: ClipKIT; IQ-TREE2 best-fit model: Q.yeast+R7

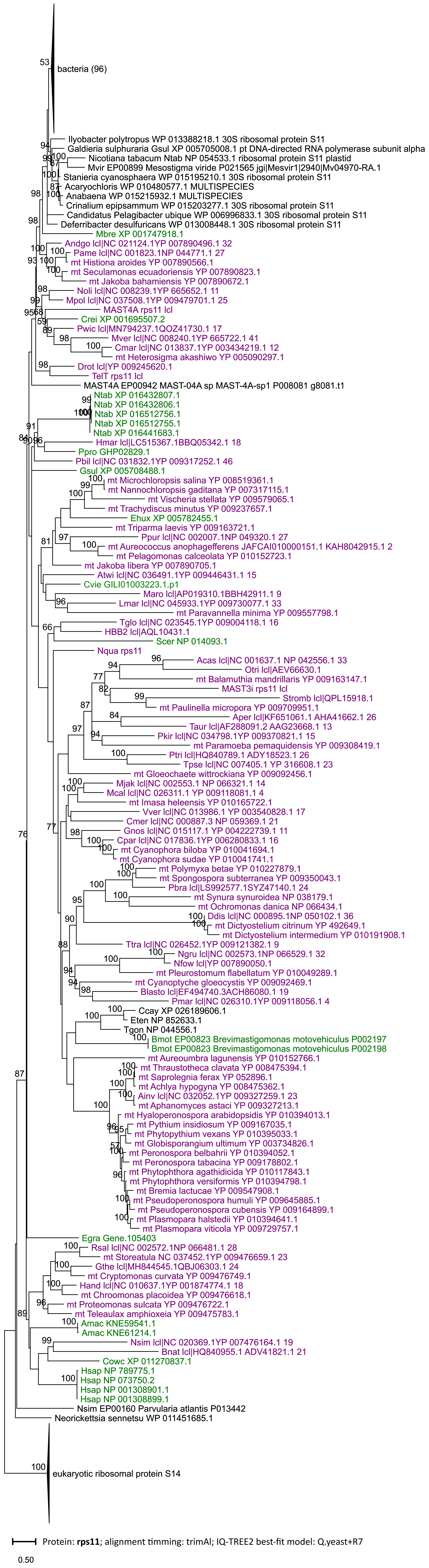

Protein: **rps11**; alignment timming: trimAl; IQ-TREE2 best-fit model: Q.yeast+R7

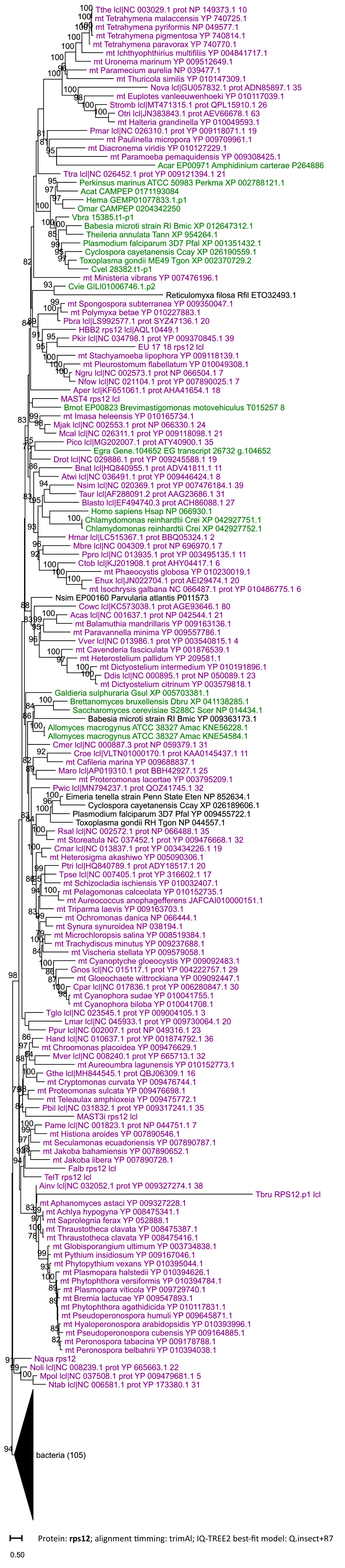
Tthe lcl|NC 003029.1 prot NP 149373.1 10
mt Tetrahymena malaccensis YP 740725.1
mt Tetrahymena pyriformis NP 049577.1
mt Tetrahymena pigmentosa YP 740814.1
mt Tetrahymena paravorax YP 740770.1
mt Ichthyophthirius multifiliis YP 004841717.1
mt Uronema marinum YP 009512649.1
mt Paramecium aurelia NP 039477.1
mt Thuricola similis YP 010147309.1
Nova lcl|GU057832.1 prot ADN85897.1 35
mt Euplotes vanleeuwenhoeki YP 010117039.1
Stromb lcl|MT471315.1 prot QPL15910.1 26
Otri lcl|JN383843.1 prot AEV66678.1 63
mt Halteria grandinella YP 010049593.1
Pmar lcl|NC 026310.1 prot YP 009118071.1 19
mt Paulinella micropora YP 009709961.1
mt Diacronema viridis YP 010127229.1
mt Paramoeba pemaquidensis YP 009308425.1
Acar EP00971 Amphidinium carterae P264886
Ttra lcl|NC 026452.1 prot YP 009121394.1 21
Perkinsus marinus ATCC 50983 Perkma XP 002788121.1
Acat CAMPEP 0171193084
Hema GEMP01077833.1.p1
Omar CAMPEP 0204342250
Vbra 15385.t1-p1
Babesia microti strain RI Bmic XP 012647312.1
Theileria annulata Tann XP 954264.1
Plasmodium falciparum 3D7 Pfal XP 001351432.1
Cyclospora cayetanensis Ccay XP 026190559.1
Toxoplasma gondii ME49 Tgon XP 002370729.2
Cvel 28382.t1-p1
mt Ministeria vibrans YP 007476196.1
Cvie GILI01006746.1.p2
Reticulomyxa filosa Rfil ETO32493.1
mt Spongospora subterranea YP 009350047.1
mt Polymyxa betae YP 010227883.1
Pbra lcl|LS992577.1 prot SYZ47136.1 20
HBB2 rps12 lcl|AQL10449.1
Pkir lcl|NC 034798.1 prot YP 009370845.1 39
EU 17 18 rps12 lcl
mt Stachyamoeba lipophora YP 009118139.1
mt Pleurostomum flabellatum YP 010049308.1
Ngru lcl|NC 002573.1 prot NP 066504.1 7
Nfow lcl|NC 021104.1 prot YP 007890025.1 7
Aper lcl|KF651061.1 prot AHA41654.1 18
MAST4 rps12 lcl
Bmot EP00823 Brevimastigomonas motovehiculus T015257 8
mt Imasa heleensis YP 010165734.1
Mjak lcl|NC 002553.1 prot NP 066330.1 24
Mcal lcl|NC 026311.1 prot YP 009118098.1 21
Pico lcl|MG202007.1 prot ATY40900.1 35
Egra Gene.104652 EG transcript 26732 g.104652
Drot lcl|NC 029886.1 prot YP 009245588.1 19
Bnat lcl|HQ840955.1 prot ADV41811.1 11
Atwi lcl|NC 036491.1 prot YP 009446424.1 8
Nsim lcl|NC 020369.1 prot YP 007476184.1 39
Taur lcl|AF288091.2 prot AAG23686.1 31
Blasto lcl|EF494740.3 prot ACH86088.1 27
Homo sapiens Hsap NP 066930.1
Chlamydomonas reinhardtii Crei XP 042927751.1
Chlamydomonas reinhardtii Crei XP 042927752.1
Hmar lcl|LC515367.1 prot BBQ05324.1 2
Mbre lcl|NC 004309.1 prot NP 696970.1 7
Ppro lcl|NC 013935.1 prot YP 003495135.1 11
Ctob lcl|KJ201908.1 prot AHY04417.1 6
mt Phaeocystis globosa YP 010230019.1
Ehux lcl|JN022704.1 prot AEI29474.1 20
mt Isochrysis galbana NC 066487.1 prot YP 010486775.1 6
Nsim EP00160 Parvularia atlantis P011573
Cowc lcl|KC573038.1 prot AGE93646.1 80
Acas lcl|NC 001637.1 prot NP 042544.1 21
mt Balamuthia mandrillaris YP 009163136.1
mt Paravannella minima YP 009557786.1
Vver lcl|NC 013986.1 prot YP 003540815.1 4
mt Cavenderia fasciculata YP 001876539.1
mt Heterostelium pallidum YP 209581.1
mt Dictyostelium intermedium YP 010191896.1
Ddis lcl|NC 000895.1 prot NP 050089.1 23
mt Dictyostelium citrinum YP 003579818.1
Galdieria sulphuraria Gsul XP 005703381.1
Brettanomyces bruxellensis Dbru XP 041138285.1
Saccharomyces cerevisiae S288C Scer NP 014434.1
Babesia microti strain RI Bmic XP 009363173.1
Allomyces macrogynus ATCC 38327 Amac KNE56228.1
Allomyces macrogynus ATCC 38327 Amac KNE54584.1
Cmer lcl|NC 000887.3 prot NP 059379.1 31
Croe lcl|VLTN01000170.1 prot KAA0145437.1 11
mt Cafileria marina YP 009688837.1
Maro lcl|AP019310.1 prot BBH42927.1 25
mt Proteromonas lacertae YP 003795209.1
Pwic lcl|MN794237.1 prot QOZ41745.1 32
Eimeria tenella strain Penn State Eten NP 852634.1
Cyclospora cayetanensis Ccay XP 026189606.1
Plasmodium falciparum 3D7 Pfal YP 009455722.1
Toxoplasma gondii RH Tgon NP 044557.1
Rsal lcl|NC 002572.1 prot NP 066488.1 35
mt Storeatula NC 037452.1 prot YP 009476668.1 32
Cmar lcl|NC 013837.1 prot YP 003434226.1 19
mt Heterosigma akashiwo YP 005090306.1
Ptri lcl|HQ840789.1 prot ADY18517.1 20
Tpse lcl|NC 007405.1 prot YP 316602.1 17
mt Schizocladia ischiensis YP 010032407.1
mt Pelagomonas calceolata YP 010152735.1
mt Aureococcus anophagefferens JAFCAI010000151.1
mt Triparma laevis YP 009163703.1
mt Ochromonas danica NP 066444.1
mt Synura synuroidea NP 038194.1
mt Microchloropsis salina YP 008519384.1
mt Trachydiscus minutus YP 009237688.1
mt Vischeria stellata YP 009579058.1
mt Cyanoptyche gloeocystis YP 009092483.1
Gnos lcl|NC 015117.1 prot YP 004222757.1 29
mt Gloeochaete wittrockiana YP 009092447.1
Cpar lcl|NC 017836.1 prot YP 006280847.1 30
mt Cyanophora sudae YP 010041755.1
mt Cyanophora biloba YP 010041708.1
Tglo lcl|NC 023545.1 prot YP 009004105.1 3
Lmar lcl|NC 045933.1 prot YP 009730064.1 20
Ppur lcl|NC 002007.1 prot NP 049316.1 23
Hand lcl|NC 010637.1 prot YP 001874792.1 36
mt Chroomonas placoidea YP 009476629.1
Mver lcl|NC 008240.1 prot YP 665713.1 32
mt Aureoumbra lagunensis YP 010152773.1
Gthe lcl|MH844545.1 prot QBJ06309.1 16
mt Cryptomonas curvata YP 009476744.1
mt Proteomonas sulcata YP 009476698.1
mt Teleaulax amphioxeia YP 009475772.1
Pbil lcl|NC 031832.1 prot YP 009317241.1 35
MAST3i rps12 lcl
Pame lcl|NC 001823.1 prot NP 044751.1 7
mt Histiona aroides YP 007890546.1
mt Seculamonas ecuadoriensis YP 007890787.1
mt Jakoba bahamiensis YP 007890652.1
mt Jakoba libera YP 007890728.1
Falb rps12 lcl
TelT rps12 lcl
Ainv lcl|NC 032052.1 prot YP 009327274.1 38
Tbru RPS12.p1 lcl
mt Aphanomyces astaci YP 009327228.1
mt Achlya hypogyna YP 008475341.1
mt Saprolegnia ferax YP 052888.1
mt Thraustotheca clavata YP 008475387.1
mt Thraustotheca clavata YP 008475416.1
mt Globisporangium ultimum YP 003734838.1
mt Pythium insidiosum YP 009167046.1
mt Phytopythium vexans YP 010395044.1
mt Plasmopara halstedii YP 010394626.1
mt Phytophthora versiformis YP 010394784.1
mt Plasmopara viticola YP 009729740.1
mt Bremia lactucae YP 009547893.1
mt Phytophthora agathidicida YP 010117831.1
mt Pseudoperonospora humuli YP 009645871.1
mt Hyaloperonospora arabidopsidis YP 010393996.1
mt Pseudoperonospora cubensis YP 009164885.1
mt Peronospora tabacina YP 009178788.1
mt Peronospora belbahrii YP 010394038.1
Nqua rps12
Noli lcl|NC 008239.1 prot YP 665663.1 22
Mpol lcl|NC 037508.1 prot YP 009479681.1 5
Ntab lcl|NC 006581.1 prot YP 173380.1 31
bacteria (105)
0.50
Protein: **rps12**; alignment timming: trimAl; IQ-TREE2 best-fit model: Q.insect+R7

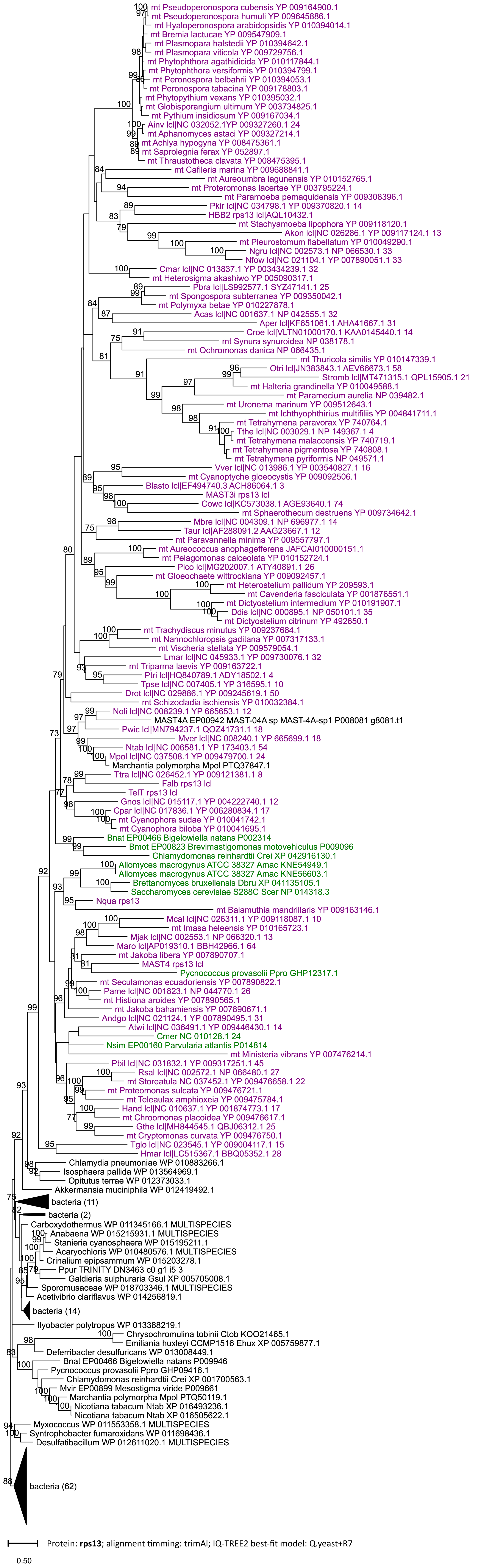

Protein: **rps13**; alignment timming: trimAl; IQ-TREE2 best-fit model: Q.yeast+R7

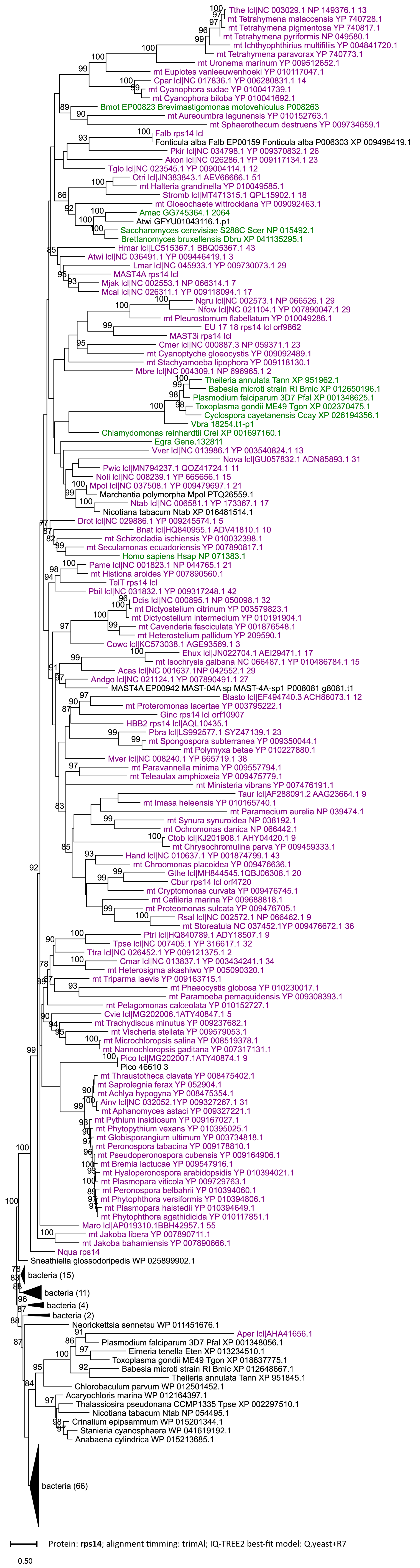

Protein: **rps14**; alignment timming: trimAl; IQ-TREE2 best-fit model: Q.yeast+R7

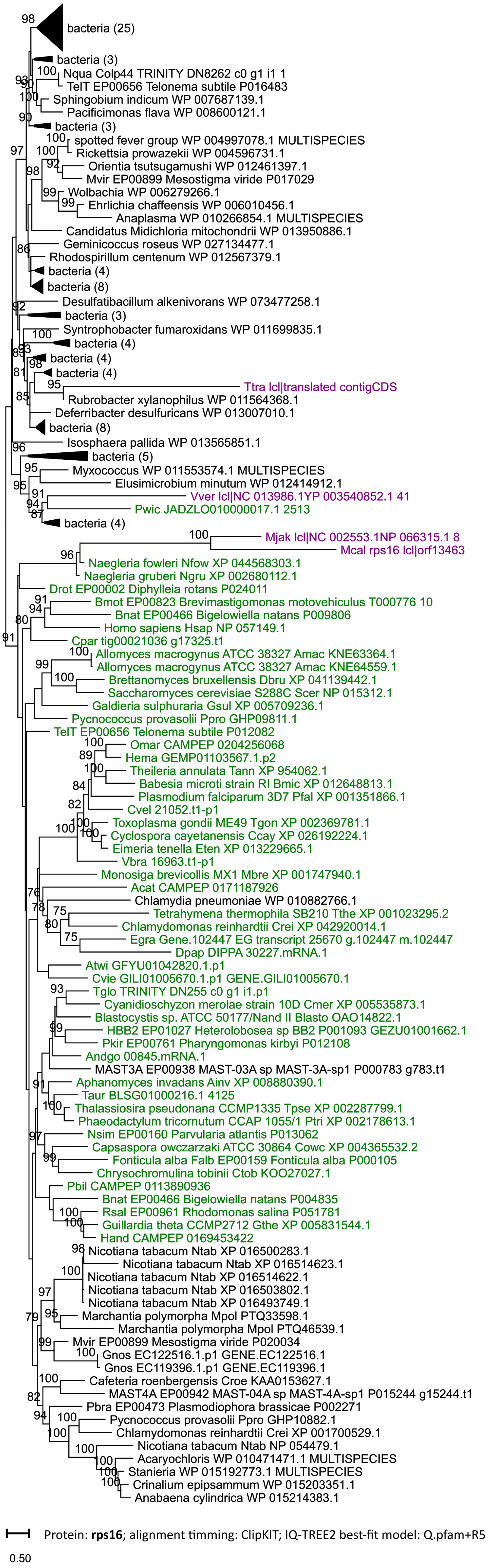

Protein: **rps16**; alignment timming: ClipKIT; IQ-TREE2 best-fit model: Q.pfam+R5

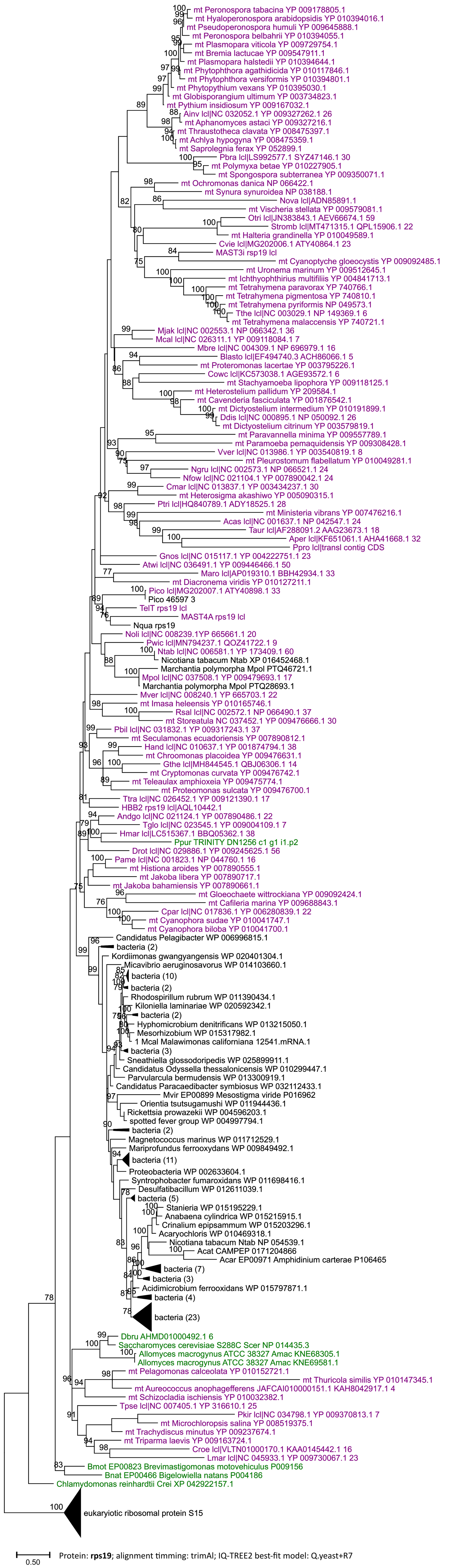

Protein: **rps19**; alignment timming: trimAl; IQ-TREE2 best-fit model: Q.yeast+R7

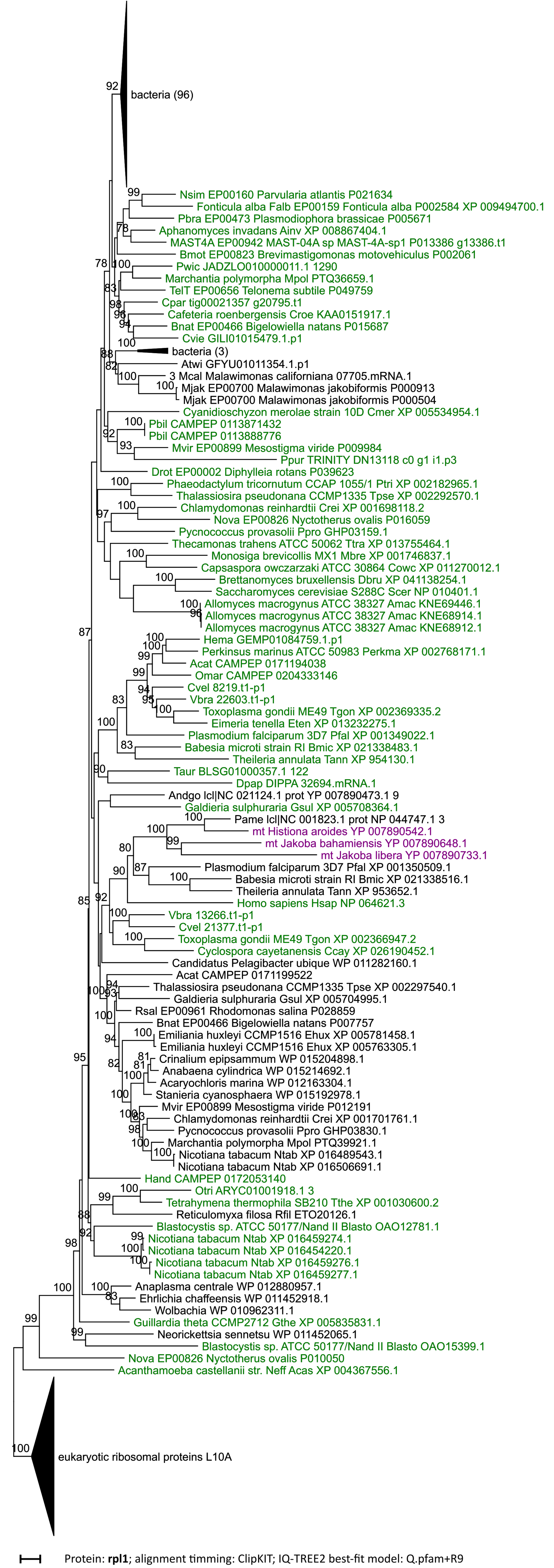

0.50 Protein: **rpl1**; alignment timming: ClipKIT; IQ-TREE2 best-fit model: Q.pfam+R9

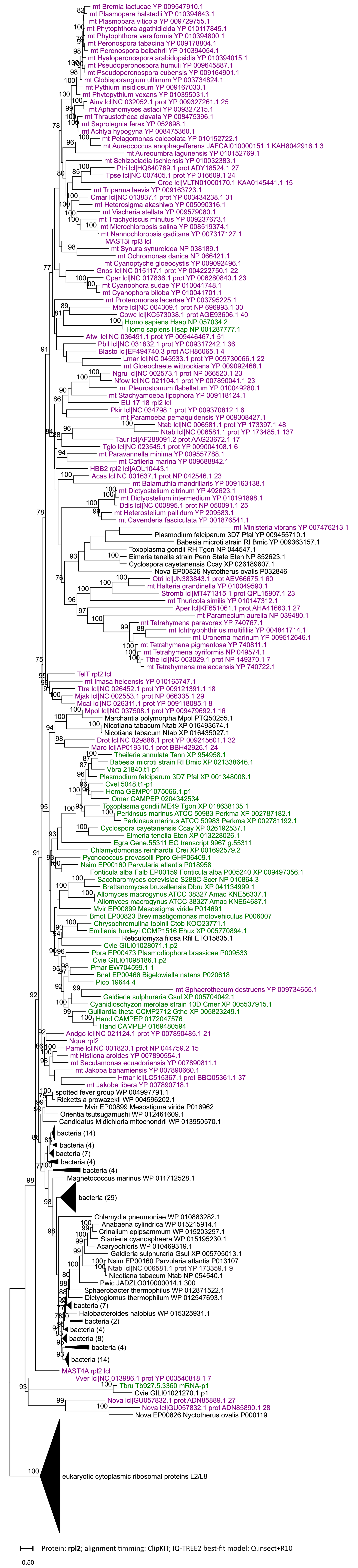

Protein: **rpl2**; alignment timming: ClipKIT; IQ-TREE2 best-fit model: Q.insect+R10

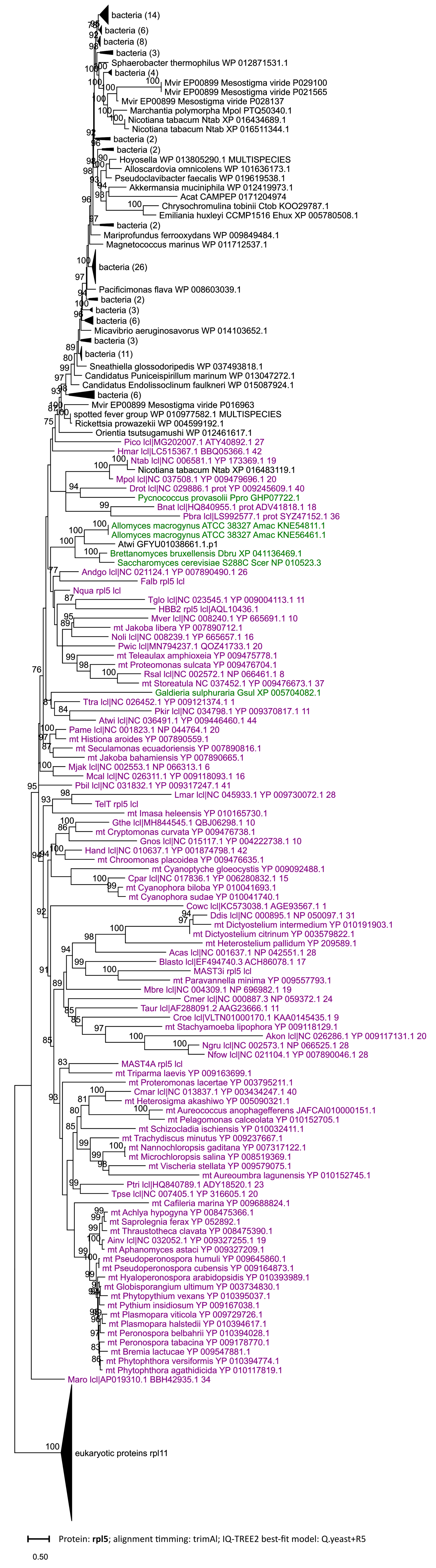

bacteria (14)
bacteria (6)
bacteria (8)
bacteria (3)
Sphaerobacter thermophilus WP 012871531.1
bacteria (4)
Mvir EP00899 Mesostigma viride P029100
Mvir EP00899 Mesostigma viride P021565
Mvir EP00899 Mesostigma viride P028137
Marchantia polymorpha Mpol PTQ50340.1
Nicotiana tabacum Ntab XP 016434689.1
Nicotiana tabacum Ntab XP 016511344.1
bacteria (2)
bacteria (2)
Hoyosella WP 013805290.1 MULTISPECIES
Alloscardovia omnicolens WP 101636173.1
Pseudoclavibacter faecalis WP 019619538.1
Akkermansia muciniphila WP 012419973.1
Acat CAMPEP 0171204974
Chrysochromulina tobinii Ctob KOO29787.1
Emiliania huxleyi CCMP1516 Ehux XP 005780508.1
bacteria (2)
Mariprofundus ferrooxydans WP 009849484.1
Magnetococcus marinus WP 011712537.1
bacteria (26)
Pacificimonas flava WP 008603039.1
bacteria (2)
bacteria (3)
bacteria (6)
Micavibrio aeruginosavorus WP 014103652.1
bacteria (3)
bacteria (11)
Sneathiella glossodoripedis WP 037493818.1
Candidatus Puniceispirillum marinum WP 013047272.1
Candidatus Endolissoclinum faulkneri WP 015087924.1
bacteria (6)
Mvir EP00899 Mesostigma viride P016963
spotted fever group WP 010977582.1 MULTISPECIES
Rickettsia prowazekii WP 004599192.1
Orientia tsutsugamushi WP 012461617.1
Pico lcl|MG202007.1 ATY40892.1 27
Hmar lcl|LC515367.1 BBQ05366.1 42
Ntab lcl|NC 006581.1 YP 173369.1 19
Nicotiana tabacum Ntab XP 016483119.1
Mpol lcl|NC 037508.1 YP 009479696.1 20
Drot lcl|NC 029886.1 prot YP 009245609.1 40
Pycnococcus provasolii Ppro GHP07722.1
Bnat lcl|HQ840955.1 prot ADV41818.1 18
Pbra lcl|LS992577.1 prot SYZ47152.1 36
Allomyces macrogynus ATCC 38327 Amac KNE54811.1
Allomyces macrogynus ATCC 38327 Amac KNE56461.1
Atwi GFYU01038661.1.p1
Brettanomyces bruxellensis Dbru XP 041136469.1
Saccharomyces cerevisiae S288C Scer NP 010523.3
Andgo lcl|NC 021124.1 YP 007890490.1 26
Falb rpl5 lcl
Nqua rpl5 lcl
Tglo lcl|NC 023545.1 YP 009004113.1 11
HBB2 rpl5 lcl|AQL10436.1
Mver lcl|NC 008240.1 YP 665691.1 10
mt Jakoba libera YP 007890712.1
Noli lcl|NC 008239.1 YP 665657.1 16
Pwic lcl|MN794237.1 QOZ41733.1 20
mt Teleaulax amphioxeia YP 009475778.1
mt Proteomonas sulcata YP 009476704.1
Rsal lcl|NC 002572.1 NP 066461.1 8
mt Storeatula NC 037452.1 YP 009476673.1 37
Galdieria sulphuraria Gsul XP 005704082.1
Ttra lcl|NC 026452.1 YP 009121374.1 1
Pkir lcl|NC 034798.1 YP 009370817.1 11
Atwi lcl|NC 036491.1 YP 009446460.1 44
Pame lcl|NC 001823.1 NP 044764.1 20
mt Histiona aroides YP 007890559.1
mt Seculamonas ecuadoriensis YP 007890816.1
mt Jakoba bahamiensis YP 007890665.1
Mjak lcl|NC 002553.1 NP 066313.1 6
Mcal lcl|NC 026311.1 YP 009118093.1 16
Pbil lcl|NC 031832.1 YP 009317247.1 41
Lmar lcl|NC 045933.1 YP 009730072.1 28
TelT rpl5 lcl
mt Imasa heleensis YP 010165730.1
Gthe lcl|MH844545.1 QBJ06298.1 10
mt Cryptomonas curvata YP 009476738.1
Gnos lcl|NC 015117.1 YP 004222738.1 10
Hand lcl|NC 010637.1 YP 001874798.1 42
mt Chroomonas placoidea YP 009476635.1
mt Cyanoptyche gloeocystis YP 009092488.1
Cpar lcl|NC 017836.1 YP 006280832.1 15
mt Cyanophora biloba YP 010041693.1
mt Cyanophora sudae YP 010041740.1
Cowc lcl|KC573038.1 AGE93567.1 1
Ddis lcl|NC 000895.1 NP 050097.1 31
mt Dictyostelium intermedium YP 010191903.1
mt Dictyostelium citrinum YP 003579822.1
mt Heterostelium pallidum YP 209589.1
Acas lcl|NC 001637.1 NP 042551.1 28
Blasto lcl|EF494740.3 ACH86078.1 17
MAST3i rpl5 lcl
mt Paravannella minima YP 009557793.1
Mbre lcl|NC 004309.1 NP 696982.1 19
Cmer lcl|NC 000887.3 NP 059372.1 24
Taur lcl|AF288091.2 AAG23666.1 11
Croe lcl|VLTN01000170.1 KAA0145435.1 9
mt Stachyamoeba lipophora YP 009118129.1
Akon lcl|NC 026286.1 YP 009117131.1 20
Ngru lcl|NC 002573.1 NP 066525.1 28
Nfow lcl|NC 021104.1 YP 007890046.1 28
MAST4A rpl5 lcl
mt Triparma laevis YP 009163699.1
mt Proteromonas lacertae YP 003795211.1
Cmar lcl|NC 013837.1 YP 003434247.1 40
mt Heterosigma akashiwo YP 005090321.1
mt Aureococcus anophagefferens JAFCAI010000151.1
mt Pelagomonas calceolata YP 010152705.1
mt Schizocladia ischiensis YP 010032411.1
mt Trachydiscus minutus YP 009237667.1
mt Nannochloropsis gaditana YP 007317122.1
mt Microchloropsis salina YP 008519369.1
mt Vischeria stellata YP 009579075.1
mt Aureoumbra lagunensis YP 010152745.1
Ptri lcl|HQ840789.1 ADY18520.1 23
Tpse lcl|NC 007405.1 YP 316605.1 20
mt Cafileria marina YP 009688824.1
mt Achlya hypogyna YP 008475366.1
mt Saprolegnia ferax YP 052892.1
mt Thraustotheca clavata YP 008475390.1
Ainv lcl|NC 032052.1 YP 009327255.1 19
mt Aphanomyces astaci YP 009327209.1
mt Pseudoperonospora humuli YP 009645860.1
mt Pseudoperonospora cubensis YP 009164873.1
mt Hyaloperonospora arabidopsidis YP 010393989.1
mt Globisporangium ultimum YP 003734830.1
mt Phytopythium vexans YP 010395037.1
mt Pythium insidiosum YP 009167038.1
mt Plasmopara viticola YP 009729726.1
mt Plasmopara halstedii YP 010394617.1
mt Peronospora belbahrii YP 010394028.1
mt Peronospora tabacina YP 009178770.1
mt Bremia lactucae YP 009547881.1
mt Phytophthora versiformis YP 010394774.1
mt Phytophthora agathidicida YP 010117819.1
Maro lcl|AP019310.1 BBH42935.1 34
eukaryotic proteins rpl11
Protein: rpl5; alignment timming: trimAl; IQ-TREE2 best-fit model: Q.yeast+R5
0.50

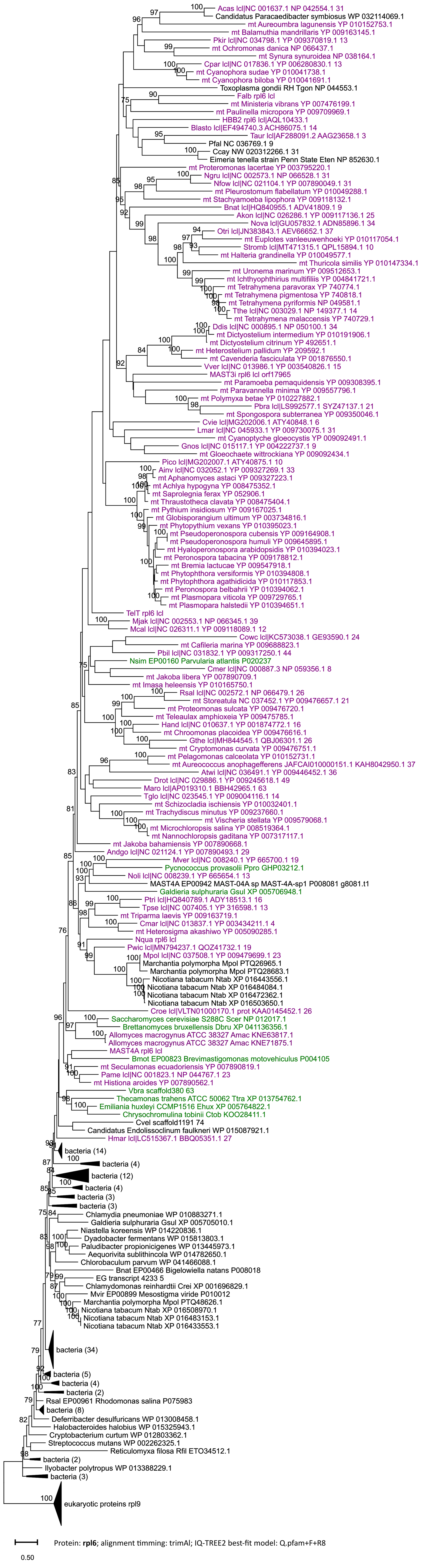

Protein: **rpl6**; alignment timming: trimAl; IQ-TREE2 best-fit model: Q.pfam+F+R8

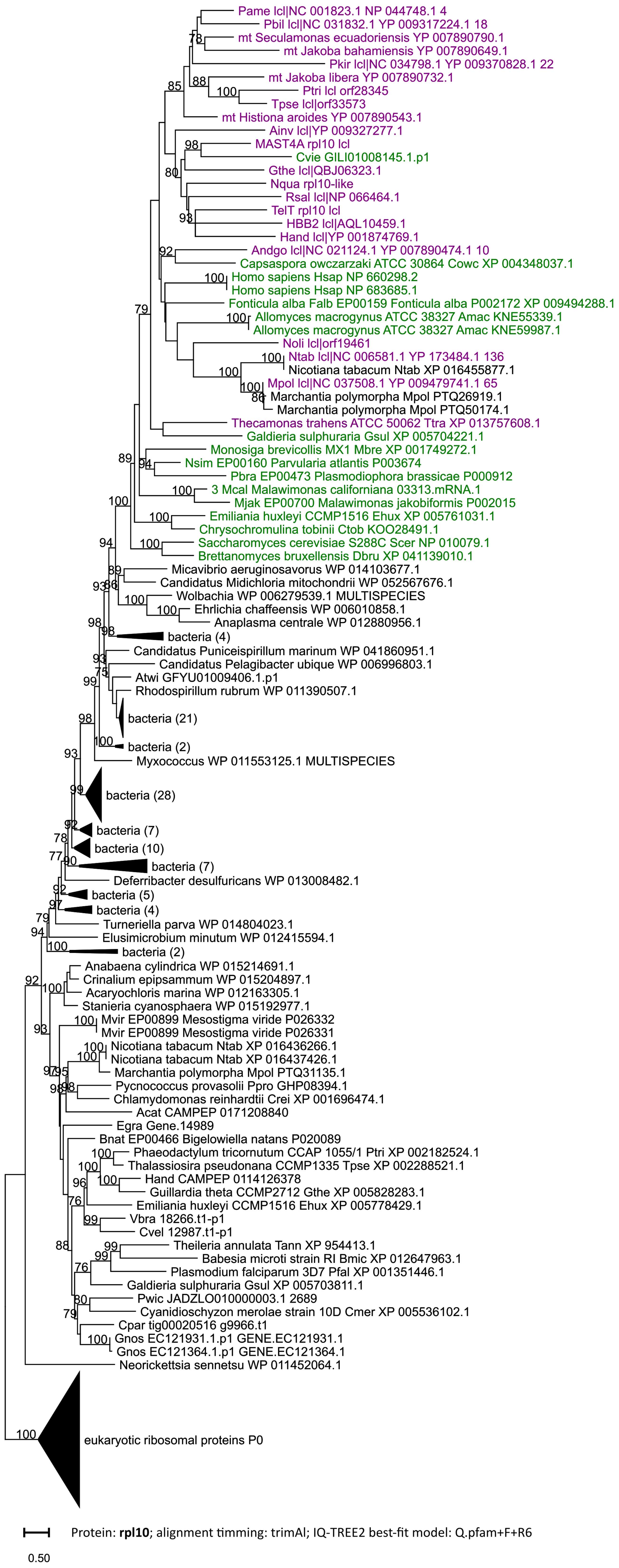

Protein: **rpl10**; alignment timming: trimAl; IQ-TREE2 best-fit model: Q.pfam+F+R6

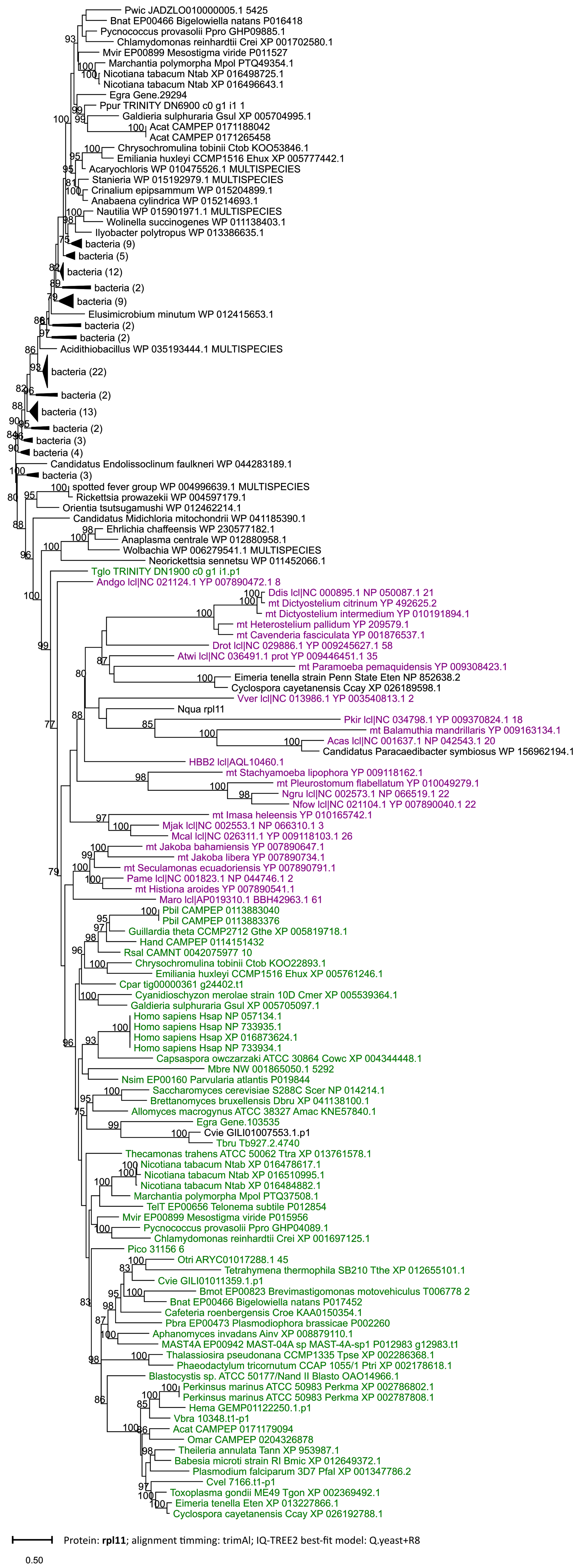

Protein: **rpl11**; alignment timming: trimAl; IQ-TREE2 best-fit model: Q.yeast+R8

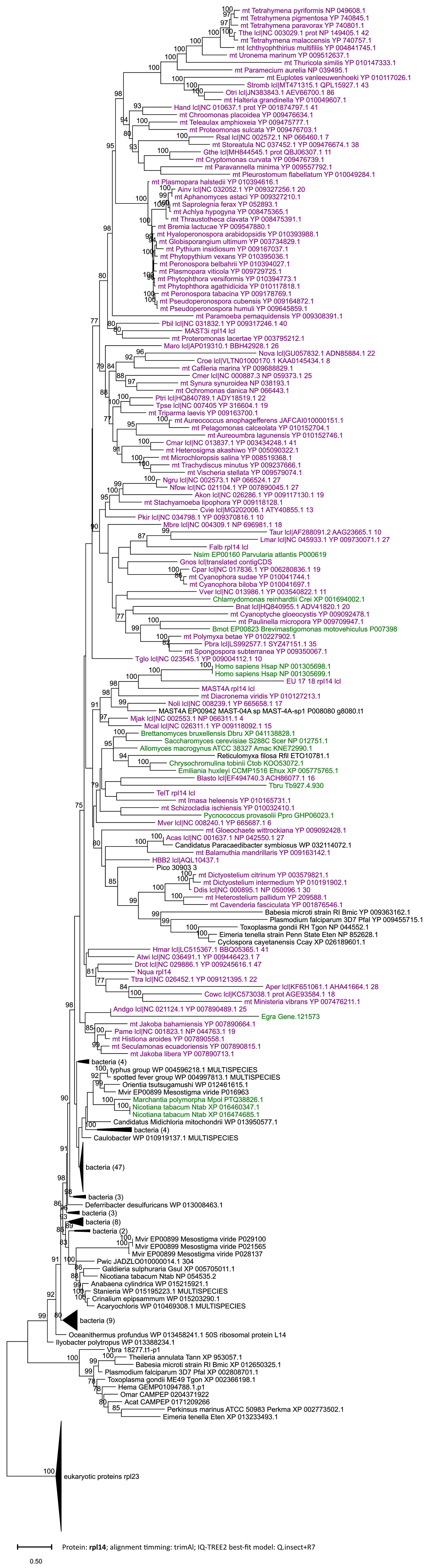

Protein: **rpl14**; alignment timming: trimAl; IQ-TREE2 best-fit model: Q.insect+R7

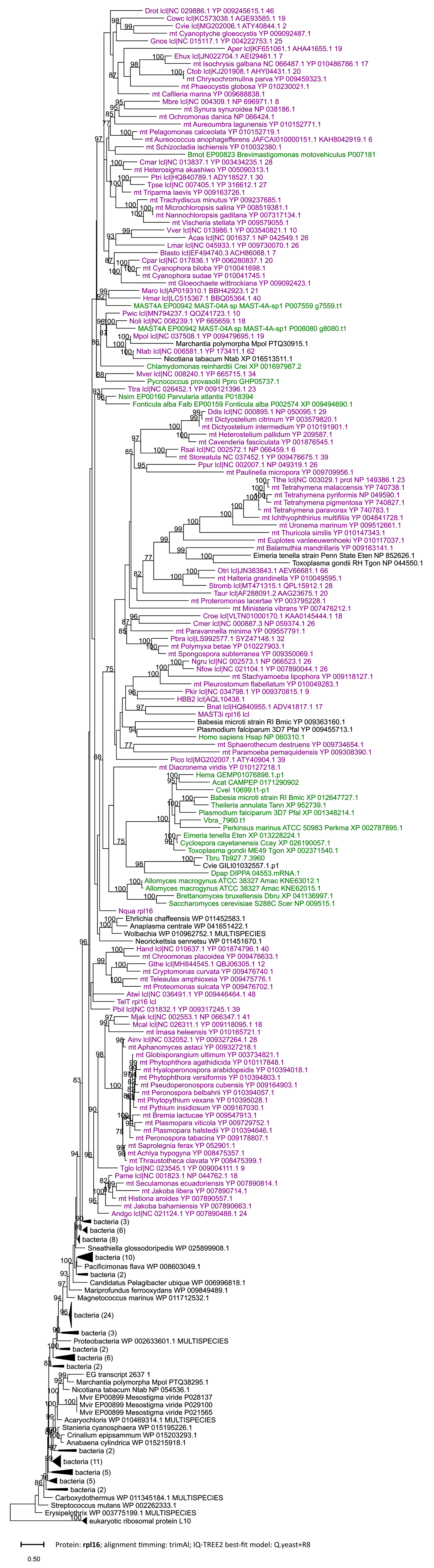

Protein: **rpl16**; alignment timming: trimAl; IQ-TREE2 best-fit model: Q.yeast+R8

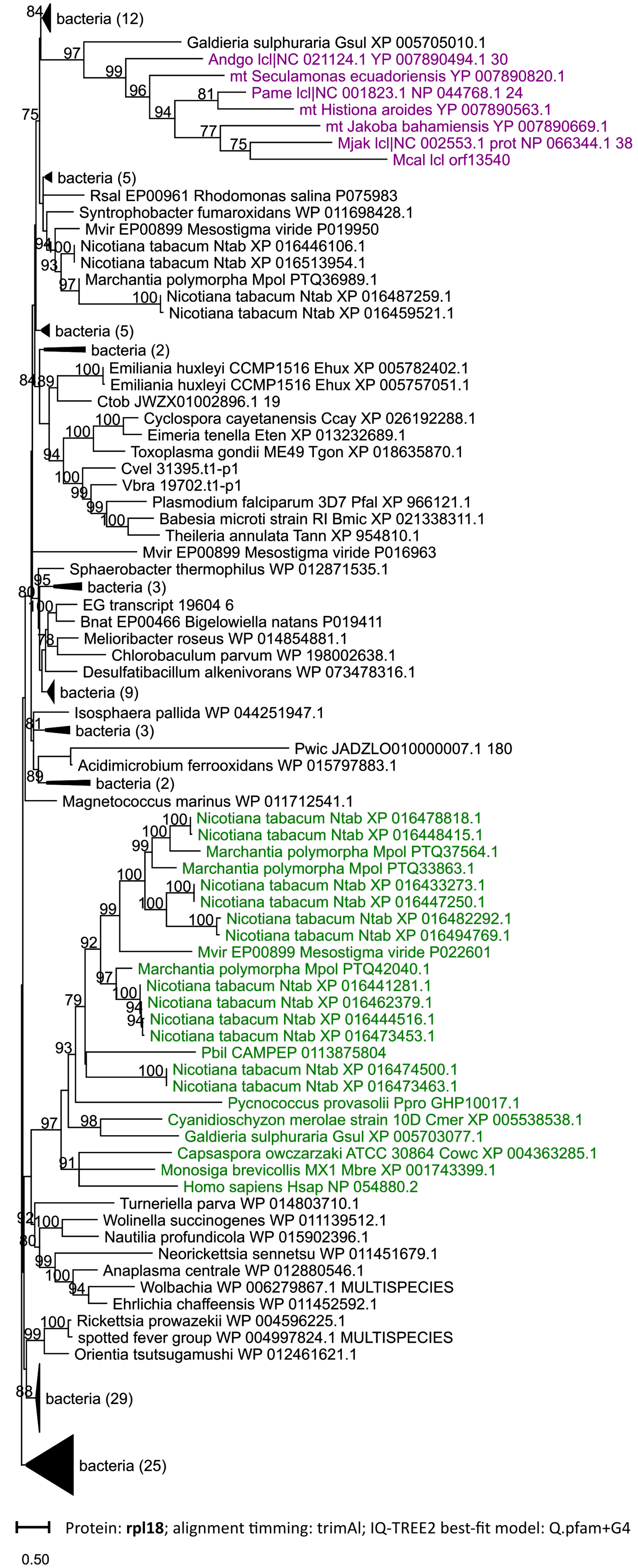

Protein: **rpl18**; alignment timming: trimAl; IQ-TREE2 best-fit model: Q.pfam+G4

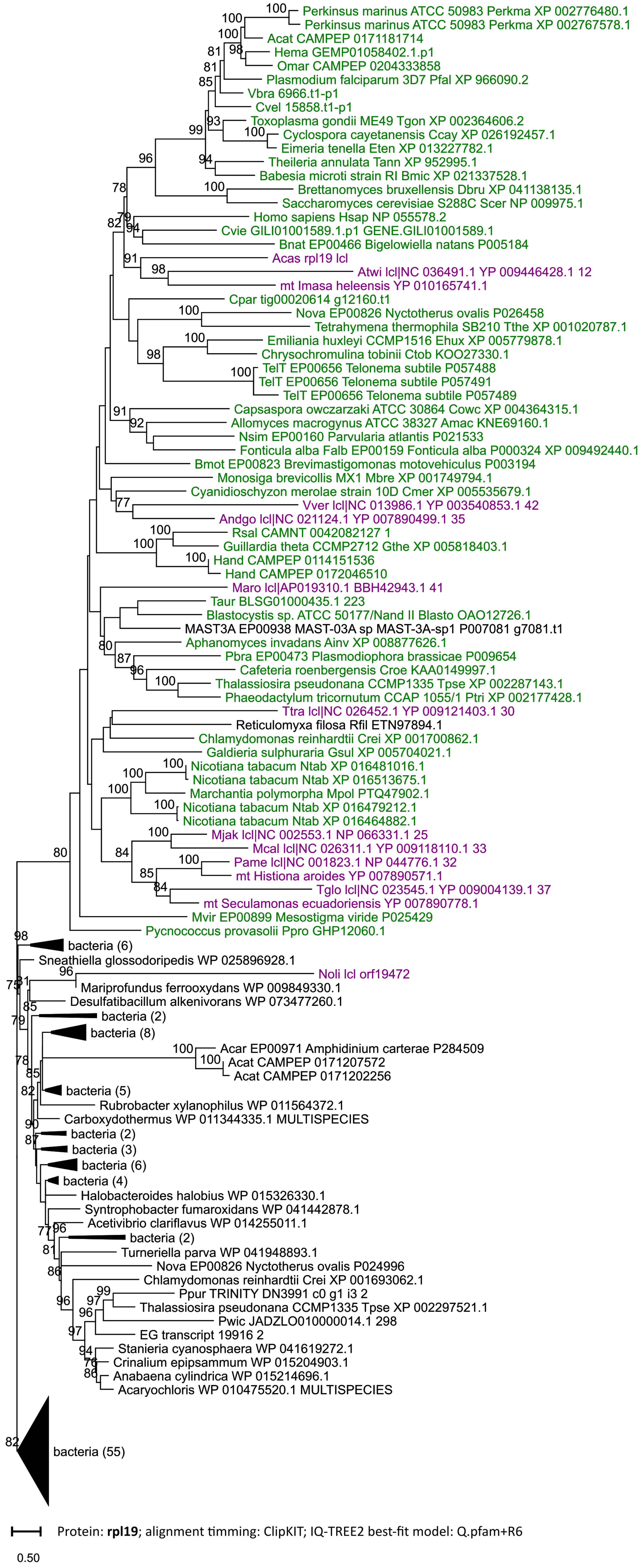

Protein: **rpl19**; alignment timming: ClipKIT; IQ-TREE2 best-fit model: Q.pfam+R6

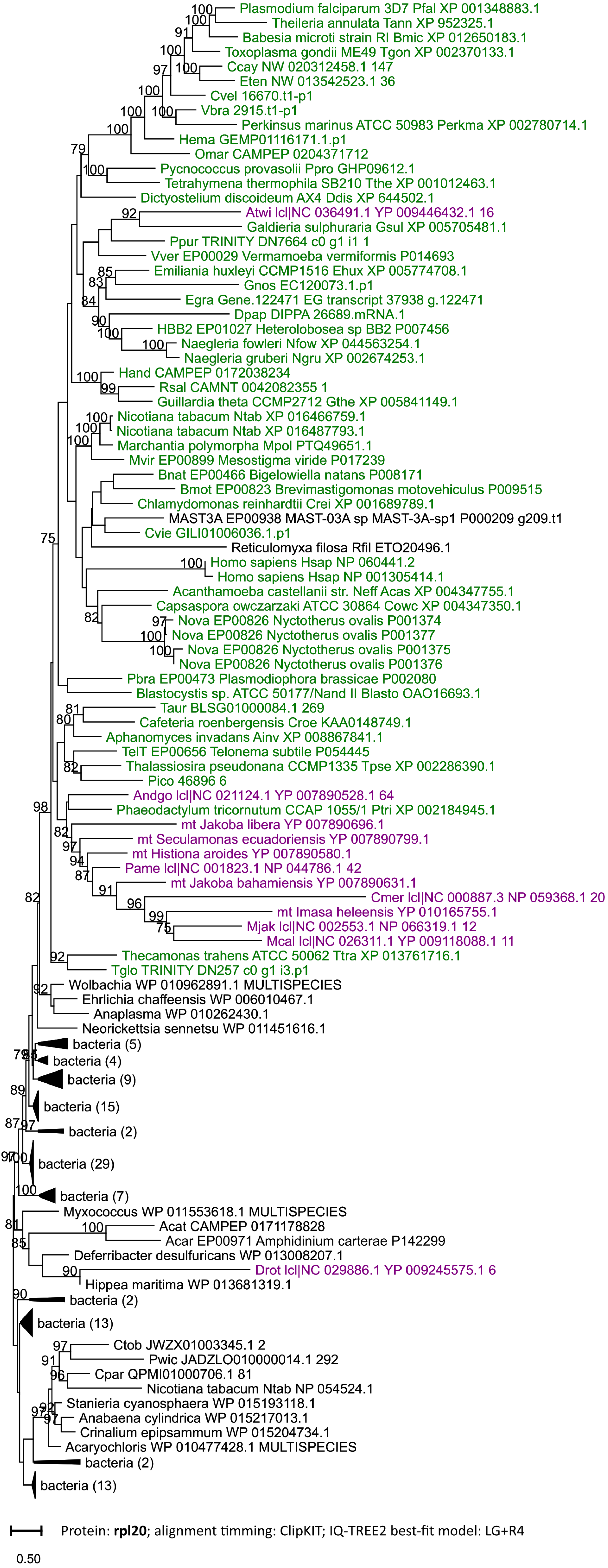

Protein: **rpl20**; alignment timming: ClipKIT; IQ-TREE2 best-fit model: LG+R4

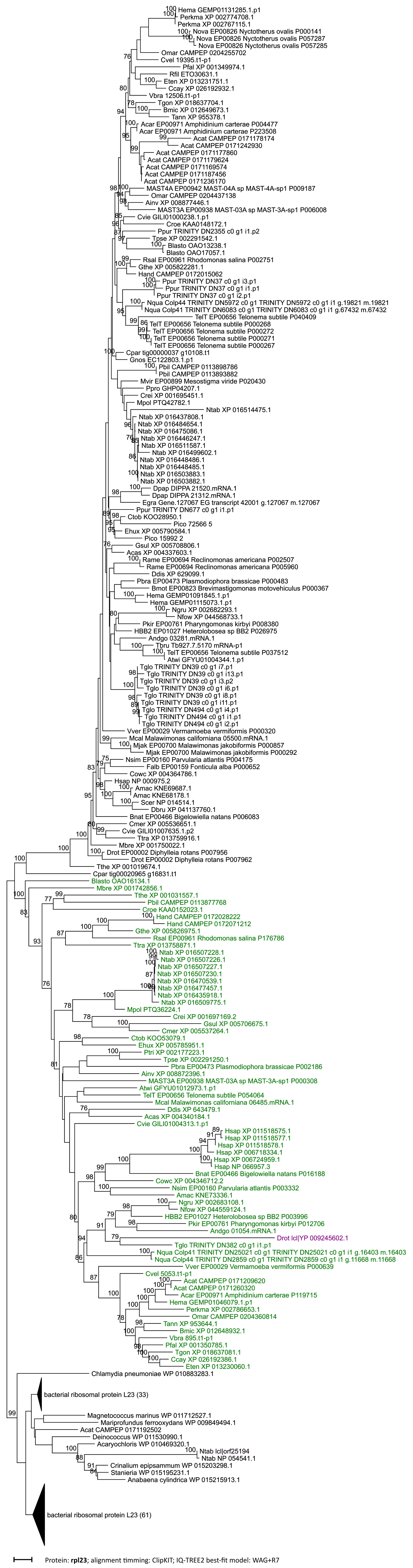

Protein: **rpl23**; alignment timming: ClipKIT; IQ-TREE2 best-fit model: WAG+R7

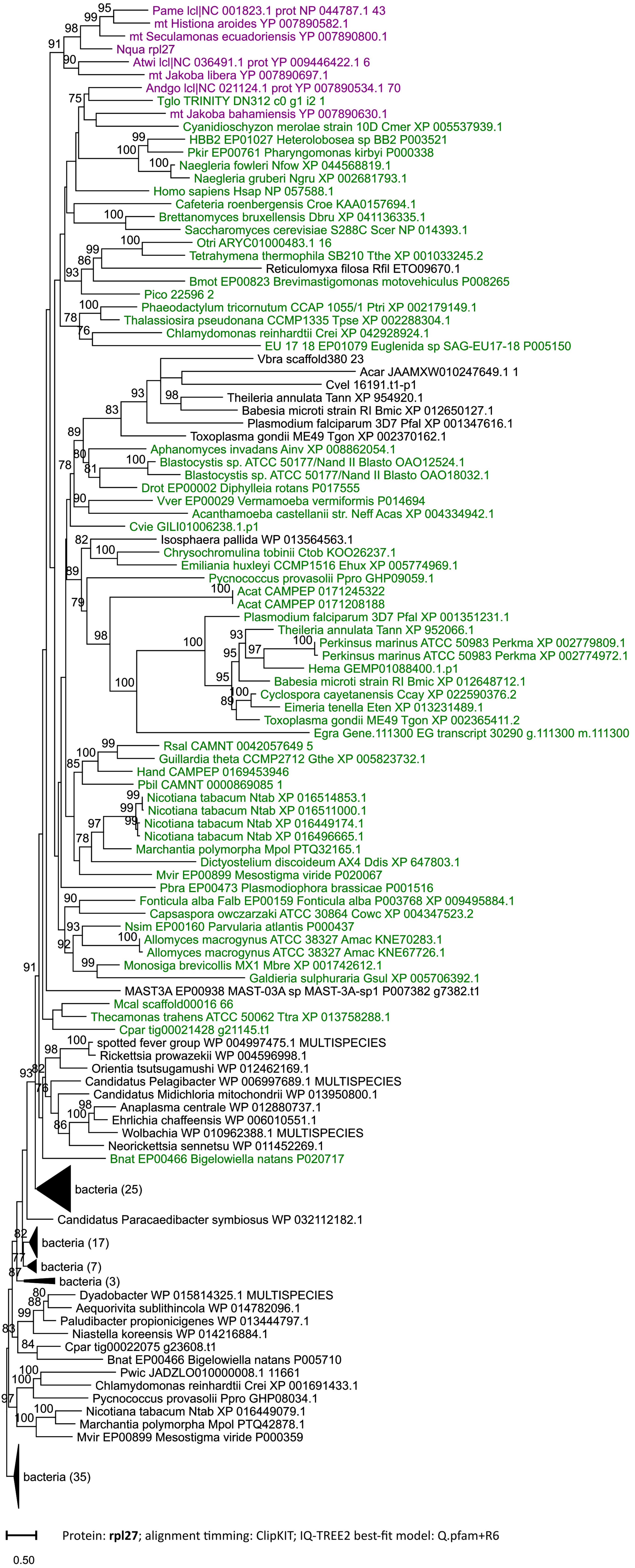

Protein: **rpl27**; alignment timming: ClipKIT; IQ-TREE2 best-fit model: Q.pfam+R6

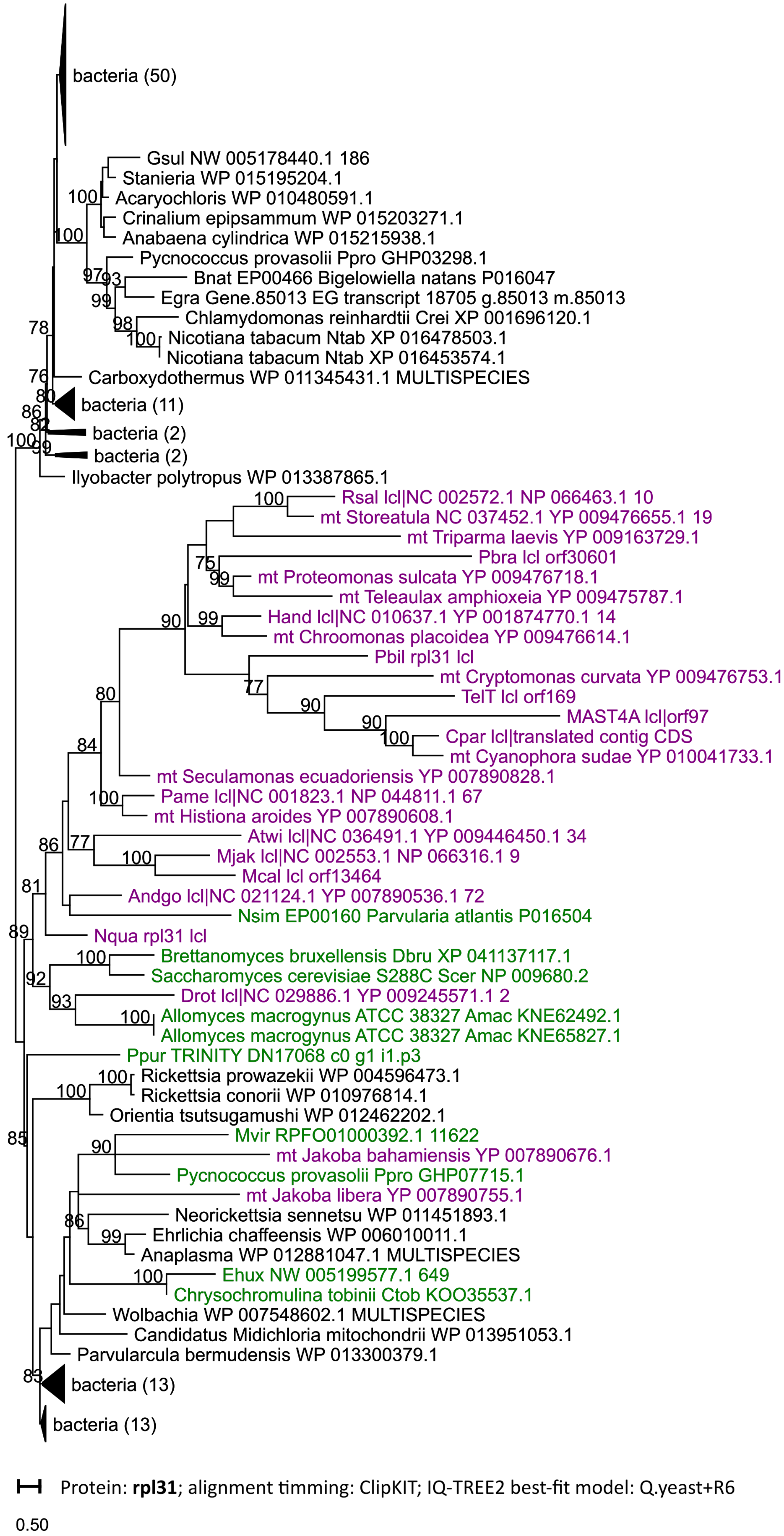

Protein: **rpl31**; alignment timming: ClipKIT; IQ-TREE2 best-fit model: Q.yeast+R6

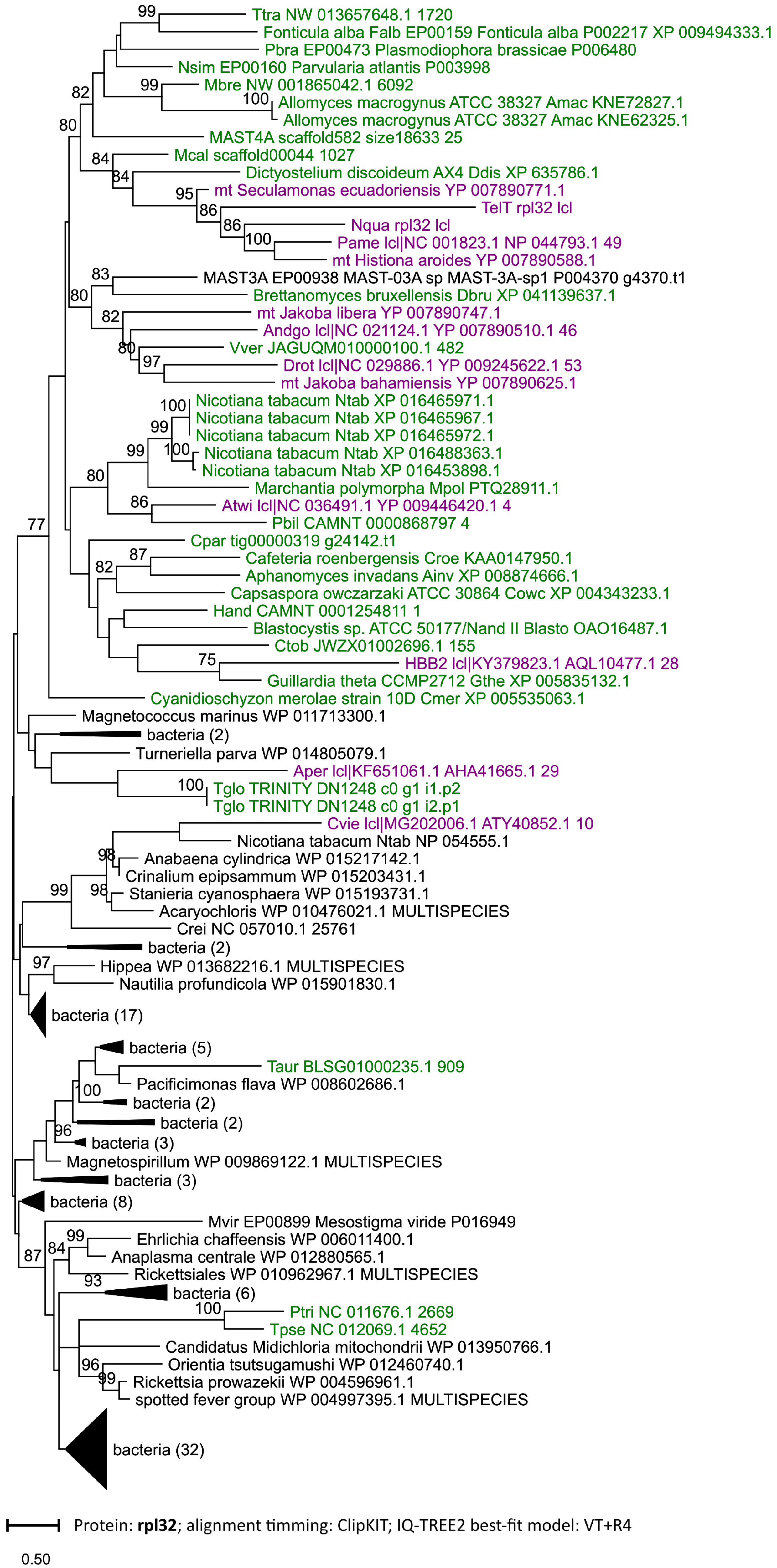

Protein: **rpl32**; alignment timming: ClipKIT; IQ-TREE2 best-fit model: VT+R4

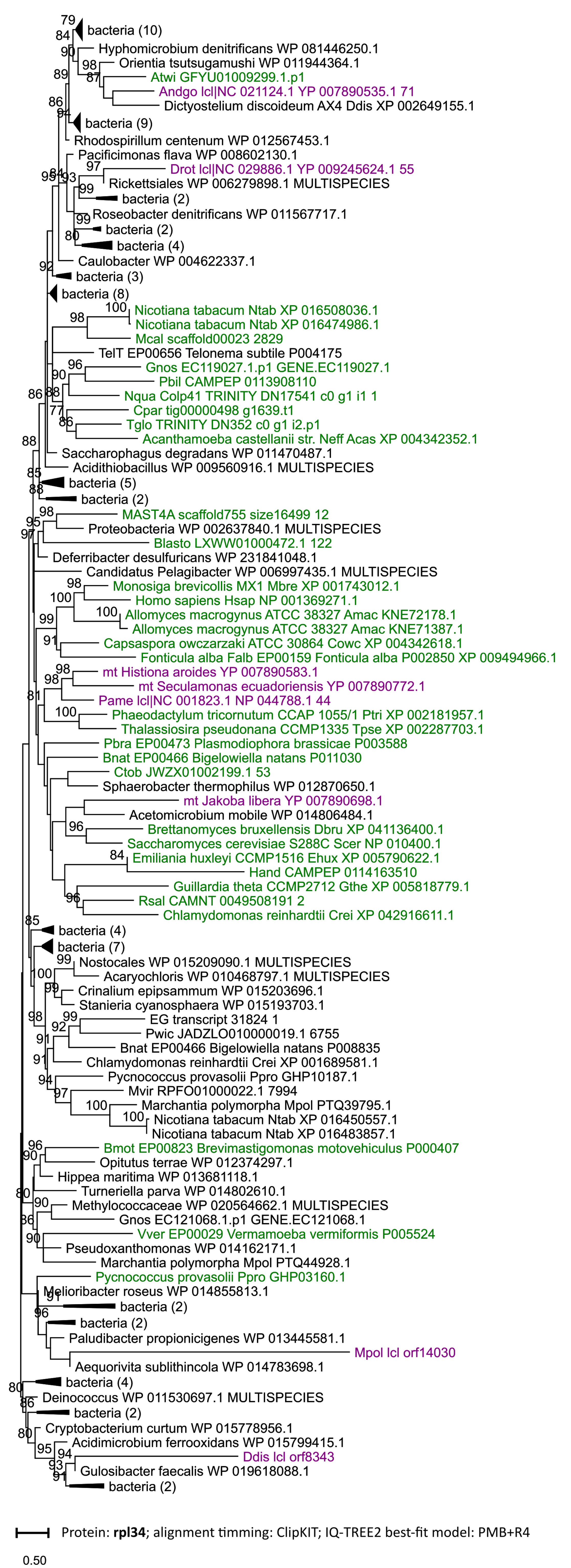

Protein: **rpl34**; alignment timming: ClipKIT; IQ-TREE2 best-fit model: PMB+R4

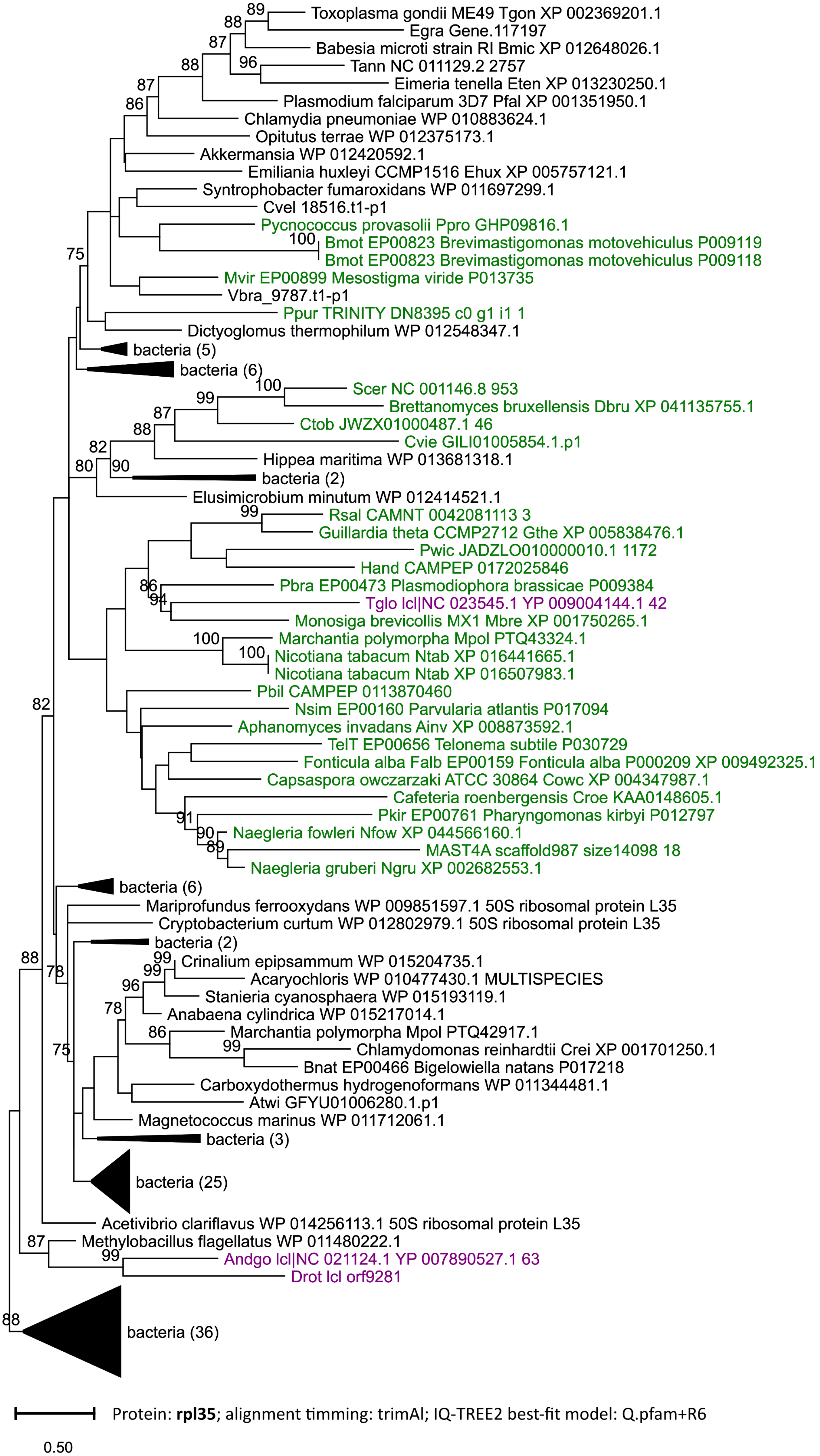

Protein: **rpl35**; alignment timming: trimAl; IQ-TREE2 best-fit model: Q.pfam+R6

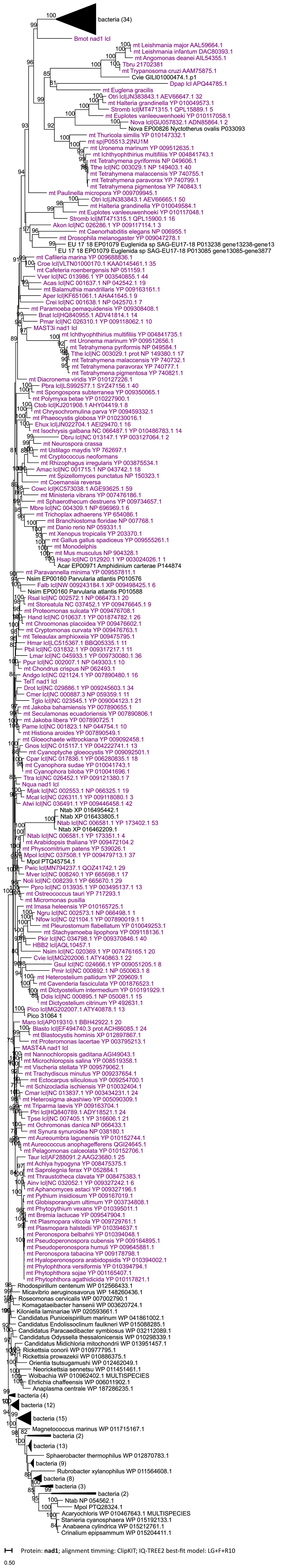

Protein: **nad1**; alignment timming: ClipKIT; IQ-TREE2 best-fit model: LG+F+R10

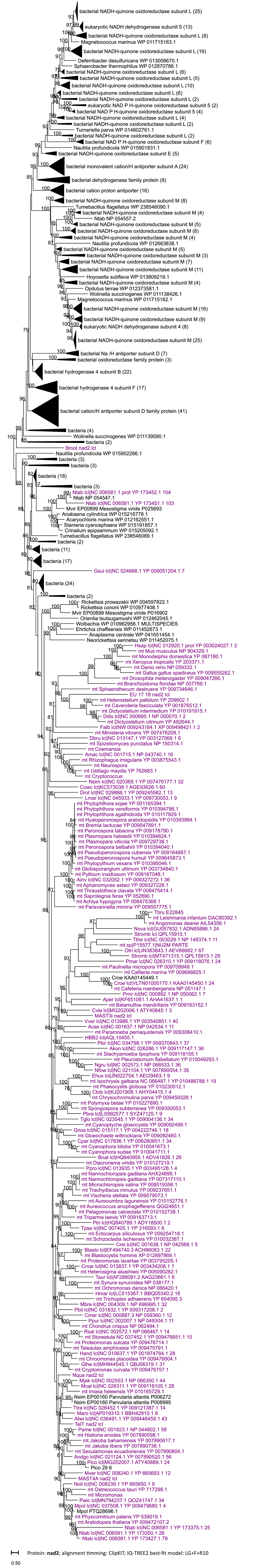

Protein: **nad2**; alignment timming: ClipKIT; IQ-TREE2 best-fit model: LG+F+R10

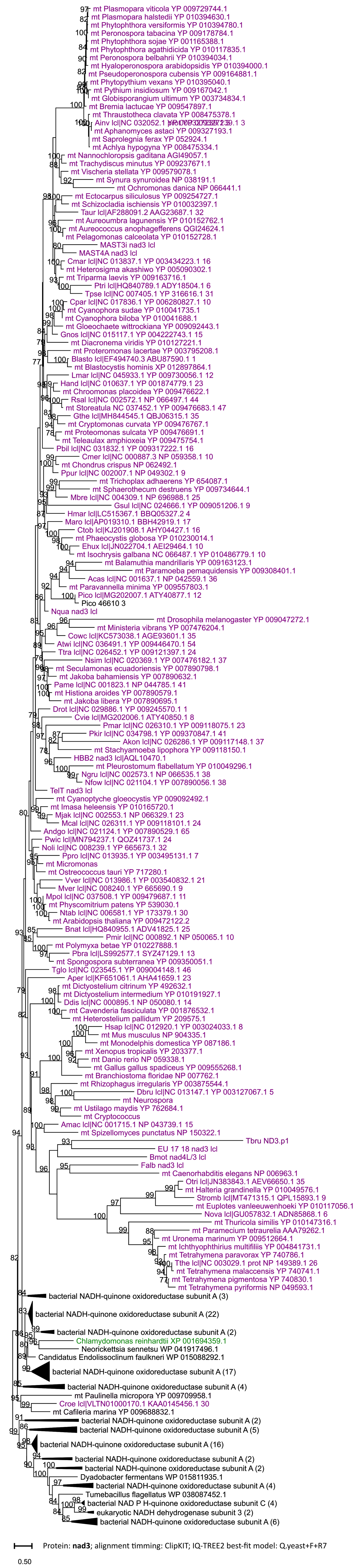

Protein: **nad3**; alignment timming: ClipKIT; IQ-TREE2 best-fit model: Q.yeast+F+R7

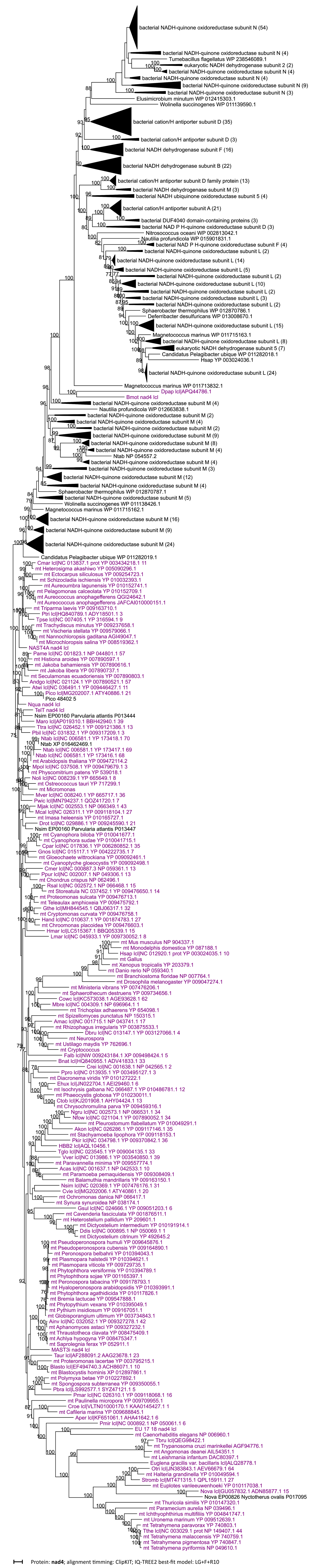

Protein: **nad4**; alignment timming: ClipKIT; IQ-TREE2 best-fit model: LG+F+R10

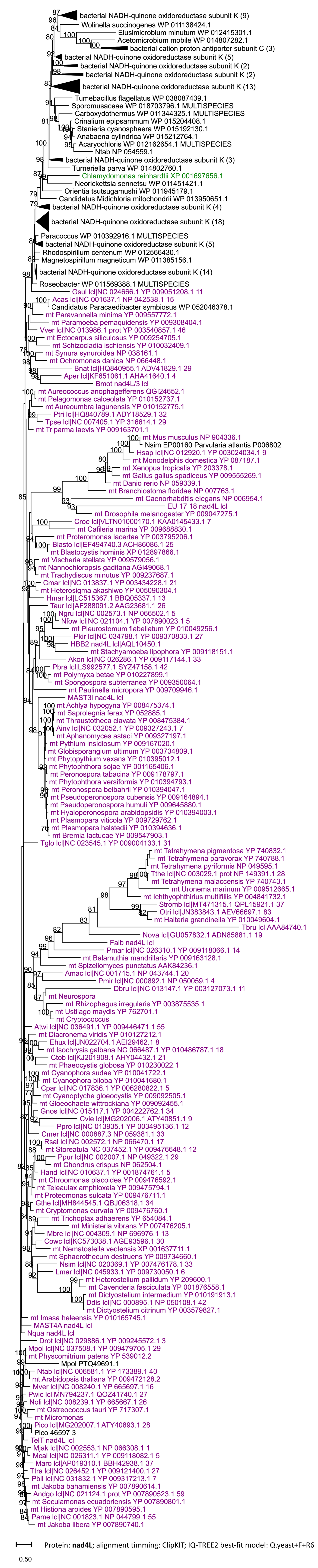

Protein: **nad4L**; alignment timming: ClipKIT; IQ-TREE2 best-fit model: Q.yeast+F+R6

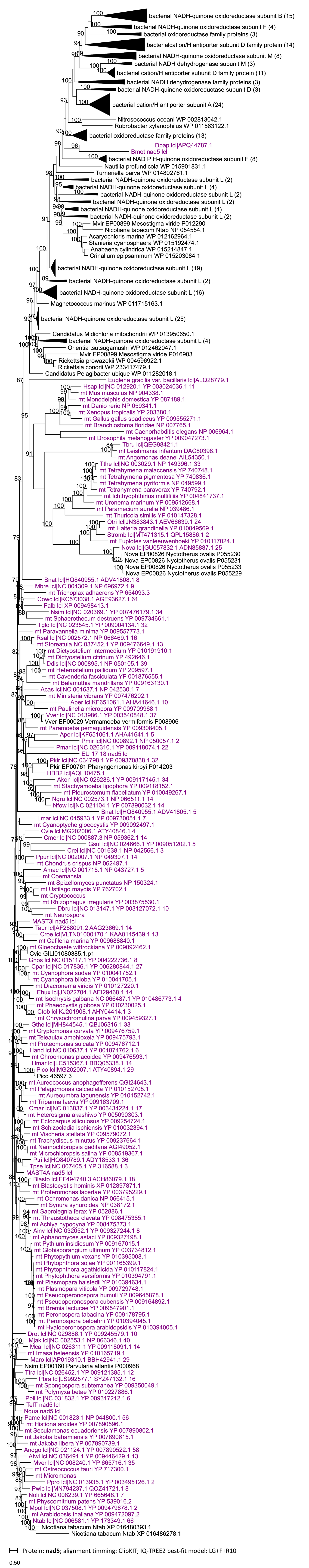

Protein: **nad5**; alignment timming: ClipKIT; IQ-TREE2 best-fit model: LG+F+R10

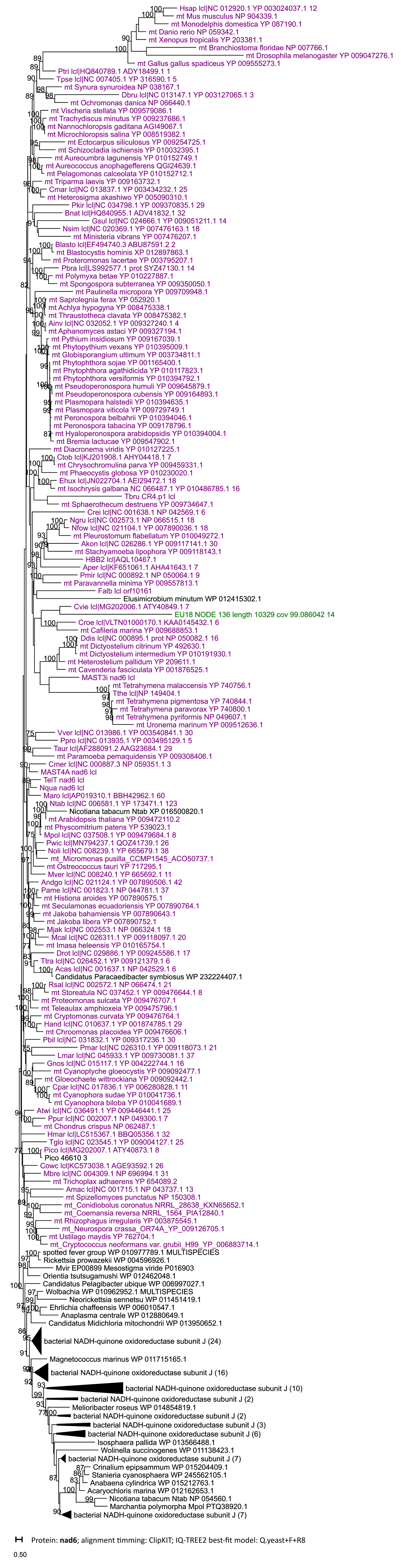

Protein: **nad6**; alignment timming: ClipKIT; IQ-TREE2 best-fit model: Q.yeast+F+R8

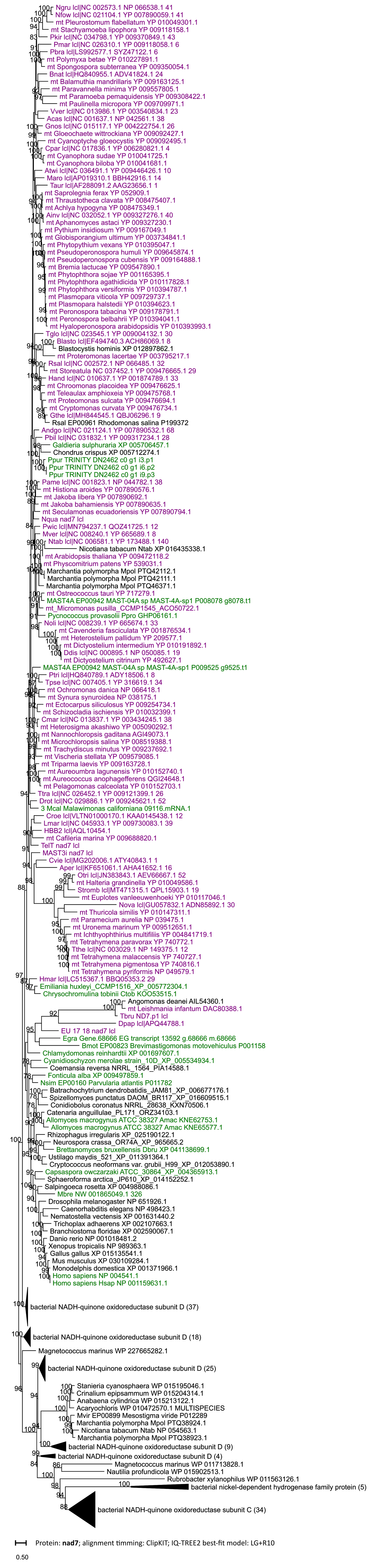
Ngru lcl|NC 002573.1 NP 066538.1 41
Nfow lcl|NC 021104.1 YP 007890059.1 41
mt Pleurostomum flabellatum YP 010049301.1
mt Stachyamoeba lipophora YP 009118158.1
Pkir lcl|NC 034798.1 YP 009370849.1 43
Pmar lcl|NC 026310.1 YP 009118058.1 6
Pbra lcl|LS992577.1 SYZ47122.1 6
mt Polymyxa betae YP 010227891.1
mt Spongospora subterranea YP 009350054.1
Bnat lcl|HQ840955.1 ADV41824.1 24
mt Balamuthia mandrillaris YP 009463125.1
mt Paravannella minima YP 009557805.1
mt Paramoeba pemaquidensis YP 009308422.1
mt Paulinella micropora YP 009709971.1
Vver lcl|NC 013986.1 YP 003540834.1 23
Acas lcl|NC 001637.1 NP 042561.1 38
Gnos lcl|NC 015117.1 YP 004222754.1 26
mt Gloeochaete wittrockiana YP 009092427.1
mt Cyanoptyche gloeocystis YP 009092495.1
Cpar lcl|NC 017836.1 YP 006280821.1 4
mt Cyanophora sudae YP 010041725.1
mt Cyanophora biloba YP 010041681.1
Atwi lcl|NC 036491.1 YP 009446426.1 10
Maro lcl|AP019310.1 BBH42916.1 14
Taur lcl|AF288091.2 AAG23656.1 1
mt Saprolegnia ferax YP 052909.1
mt Thraustotheca clavata YP 008475407.1
mt Achlya hypogyna YP 008475349.1
Ainv lcl|NC 032052.1 YP 009327276.1 40
mt Aphanomyces astaci YP 009327230.1
mt Pythium insidiosum YP 009167049.1
mt Globisporangium ultimum YP 003734841.1
mt Phytopythium vexans YP 010395047.1
mt Pseudoperonospora humuli YP 009645874.1
mt Pseudoperonospora cubensis YP 009164888.1
mt Bremia lactucae YP 009547890.1
mt Phytophthora sojae YP 001165395.1
mt Phytophthora agathidicida YP 010117828.1
mt Phytophthora versiformis YP 010394787.1
mt Plasmopara viticola YP 009729737.1
mt Plasmopara halstedii YP 010394623.1
mt Peronospora tabacina YP 009178791.1
mt Peronospora belbahrii YP 010394041.1
mt Hyaloperonospora arabidopsidis YP 010393993.1
Tglo lcl|NC 023545.1 YP 009004132.1 30
Blasto lcl|EF494740.3 ACH86069.1 8
Blastocystis hominis XP 012897862.1
mt Proteromonas lacertae YP 003795217.1
Rsal lcl|NC 002572.1 NP 066485.1 32
mt Storeatula NC 037452.1 YP 009476665.1 29
Hand lcl|NC 010637.1 YP 001874789.1 33
mt Chroomonas placoidea YP 009476625.1
mt Teleaulax amphioxeia YP 009475768.1
mt Proteomonas sulcata YP 009476694.1
mt Cryptomonas curvata YP 009476734.1
Gthe lcl|MH844545.1 QBJ06296.1 9
Rsal EP00961 Rhodomonas salina P199372
Andgo lcl|NC 021124.1 YP 007890532.1 68
Pbil lcl|NC 031832.1 YP 009317234.1 28
Galdieria sulphuraria XP 005706457.1
Chondrus crispus XP 005712274.1
Ppur TRINITY DN2462 c0 g1 i3.p1
Ppur TRINITY DN2462 c0 g1 i6.p2
Ppur TRINITY DN2462 c0 g1 i9.p3
Pame lcl|NC 001823.1 NP 044782.1 38
mt Histiona aroides YP 007890576.1
mt Jakoba libera YP 007890692.1
mt Jakoba bahamiensis YP 007890635.1
mt Seculamonas ecuadoriensis YP 007890794.1
Nqua nad7 lcl
Pwic lcl|MN794237.1 QOZ41725.1 12
Mver lcl|NC 008240.1 YP 665689.1 8
Ntab lcl|NC 006581.1 YP 173488.1 140
Nicotiana tabacum Ntab XP 016435338.1
mt Arabidopsis thaliana YP 009472118.2
mt Physcomitrium patens YP 539031.1
Marchantia polymorpha Mpol PTQ42112.1
Marchantia polymorpha Mpol PTQ42111.1
Marchantia polymorpha Mpol PTQ46371.1
mt Ostreococcus tauri YP 717279.1
MAST4A EP00942 MAST-04A sp MAST-4A-sp1 P008078 g8078.t1
mt_Micromonas pusilla_CCMP1545_ACO50722.1
Pycnococcus provasolii Ppro GHP06161.1
Noli lcl|NC 008239.1 YP 665674.1 33
mt Cavenderia fasciculata YP 001876534.1
mt Heterostelium pallidum YP 209577.1
mt Dictyostelium intermedium YP 010191892.1
Ddis lcl|NC 000895.1 NP 050085.1 19
mt Dictyostelium citrinum YP 492627.1
MAST4A EP00942 MAST-04A sp MAST-4A-sp1 P009525 g9525.t1
Ptri lcl|HQ840789.1 ADY18506.1 8
Tpse lcl|NC 007405.1 YP 316619.1 34
mt Ochromonas danica NP 066418.1
mt Synura synuroidea NP 038175.1
mt Ectocarpus siliculosus YP 009254734.1
mt Schizocladia ischiensis YP 010032399.1
Cmar lcl|NC 013837.1 YP 003434245.1 38
mt Heterosigma akashiwo YP 005090292.1
mt Nannochloropsis gaditana AGI49073.1
mt Microchloropsis salina YP 008519388.1
mt Trachydiscus minutus YP 009237692.1
mt Vischeria stellata YP 009579085.1
mt Triparma laevis YP 009163728.1
mt Aureoumbra lagunensis YP 010152740.1
mt Aureococcus anophagefferens QGI24648.1
mt Pelagomonas calceolata YP 010152703.1
Ttra lcl|NC 026452.1 YP 009121399.1 26
Drot lcl|NC 029886.1 YP 009245621.1 52
3 Mcal Malawimonas californiana 09116.mRNA.1
Croe lcl|VLTN01000170.1 KAA0145438.1 12
Lmar lcl|NC 045933.1 YP 009730083.1 39
HBB2 lcl|AQL10454.1
mt Cafileria marina YP 009688820.1
TelT nad7 lcl
MAST3i nad7 lcl
Cvie lcl|MG202006.1 ATY40843.1 1
Aper lcl|KF651061.1 AHA41652.1 16
Otri lcl|JN383843.1 AEV66667.1 52
mt Halteria grandinella YP 010049586.1
Stromb lcl|MT471315.1 QPL15903.1 19
mt Euplotes vanleeuwenhoeki YP 010117046.1
Nova lcl|GU057832.1 ADN85892.1 30
mt Thuricola similis YP 010147311.1
mt Paramecium aurelia NP 039475.1
mt Uronema marinum YP 009512651.1
mt Ichthyophthirius multifiliis YP 004841719.1
mt Tetrahymena paravorax YP 740772.1
Tthe lcl|NC 003029.1 NP 149375.1 12
mt Tetrahymena malaccensis YP 740727.1
mt Tetrahymena pigmentosa YP 740816.1
mt Tetrahymena pyriformis NP 049579.1
Hmar lcl|LC515367.1 BBQ05353.2 29
Emiliania huxleyi_CCMP1516_XP_005772304.1
Chrysochromulina tobinii Ctob KOO53515.1
Angomonas deanei AIL54360.1
mt Leishmania infantum DAC80388.1
Tbru ND7.p1 lcl
Dpap lcl|APQ44788.1
EU 17 18 nad7 lcl
Egra Gene.68666 EG transcript 13592 g.68666 m.68666
Bmot EP00823 Brevimastigomonas motovehiculus P001158
Chlamydomonas reinhardtii XP 001697607.1
Cyanidioschyzon merolae strain_10D_XP_005534934.1
Coemansia reversa NRRL_1564_PIA14588.1
Fonticula alba XP 009497859.1
Nsim EP00160 Parvularia atlantis P011782
Batrachochytrium dendrobatidis_JAM81_XP_006677176.1
Spizellomyces punctatus DAOM_BR117_XP_016609515.1
Conidiobolus coronatus NRRL_28638_KXN70506.1
Catenaria anguillulae_PL171_ORZ34103.1
Allomyces macrogynus ATCC 38327 Amac KNE62753.1
Allomyces macrogynus ATCC 38327 Amac KNE65577.1
Rhizophagus irregularis XP_025190122.1
Neurospora crassa_OR74A_XP_965665.2
Brettanomyces bruxellensis Dbru XP 041138699.1
Ustilago maydis_521_XP_011391364.1
Cryptococcus neoformans var. grubii_H99_XP_012053890.1
Capsaspora owczarzaki ATCC_30864_XP_004365913.1
Sphaeroforma arctica_JP610_XP_014152252.1
Salpingoeca rosetta XP 004988086.1
Mbre NW 001865049.1 326
Drosophila melanogaster NP 651926.1
Caenorhabditis elegans NP 498423.1
Nematostella vectensis XP 001631440.2
Trichoplax adhaerens XP 002107663.1
Branchiostoma floridae XP 002590067.1
Danio rerio NP 001018481.2
Xenopus tropicalis NP 989363.1
Gallus gallus XP 015135541.1
Mus musculus XP 030109284.1
Monodelphis domestica XP 001371966.1
Homo sapiens NP 004541.1
Homo sapiens Hsap NP 001159631.1
bacterial NADH-quinone oxidoreductase subunit D (37)
bacterial NADH-quinone oxidoreductase subunit D (18)
Magnetococcus marinus WP 227665282.1
bacterial NADH-quinone oxidoreductase subunit D (25)
Stanieria cyanosphaera WP 015195046.1
Crinalium epipsammum WP 015204314.1
Anabaena cylindrica WP 015213122.1
Acaryochloris WP 010472570.1 MULTISPECIES
Mvir EP00899 Mesostigma viride P012289
Marchantia polymorpha Mpol PTQ38924.1
Nicotiana tabacum Ntab NP 054563.1
Marchantia polymorpha Mpol PTQ38923.1
bacterial NADH-quinone oxidoreductase subunit D (9)
bacterial NADH-quinone oxidoreductase subunit D (4)
Magnetococcus marinus WP 011713828.1
Nautilia profundicola WP 015902513.1
Rubrobacter xylanophilus WP 011563126.1
bacterial nickel-dependent hydrogenase family protein (5)
bacterial NADH-quinone oxidoreductase subunit C (34)
Protein: nad7; alignment timming: ClipKIT; IQ-TREE2 best-fit model: LG+R10
0.50

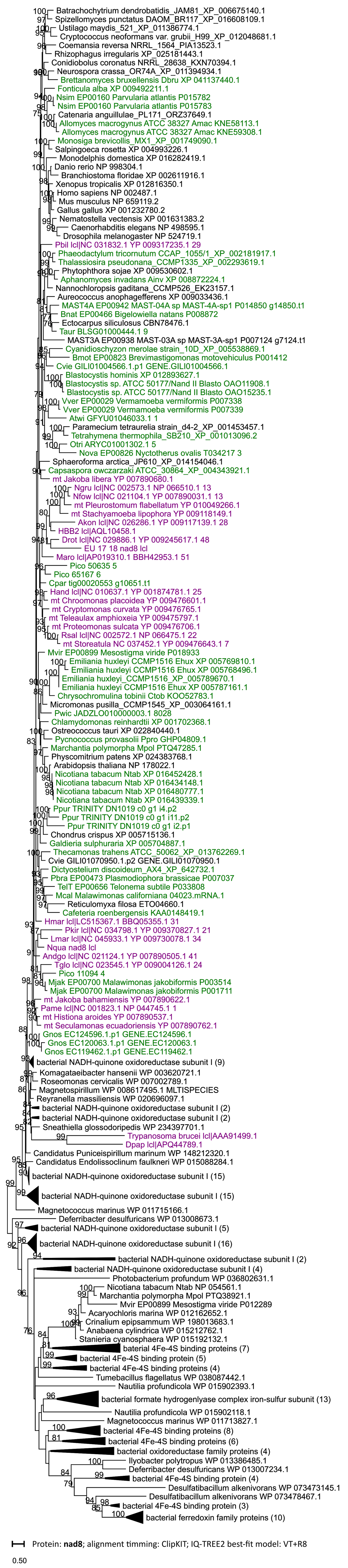
Batrachochytrium dendrobatidis_JAM81_XP_006675140.1
Spizellomyces punctatus DAOM_BR117_XP_016608109.1
Ustilago maydis_521_XP_011386774.1
Cryptococcus neoformans var. grubii_H99_XP_012048681.1
Coemansia reversa NRRL_1564_PIA13523.1
Rhizophagus irregularis XP_025181443.1
Conidiobolus coronatus NRRL_28638_KXN70394.1
Neurospora crassa_OR74A_XP_011394934.1
Brettanomyces bruxellensis Dbru XP 041137440.1
Fonticula alba XP 009492211.1
Nsim EP00160 Parvularia atlantis P015782
Nsim EP00160 Parvularia atlantis P015783
Catenaria anguillulae_PL171_ORZ37649.1
Allomyces macrogynus ATCC 38327 Amac KNE58113.1
Allomyces macrogynus ATCC 38327 Amac KNE59308.1
Monosiga brevicollis_MX1_XP_001749090.1
Salpingoeca rosetta XP 004993226.1
Monodelphis domestica XP 016282419.1
Danio rerio NP 998304.1
Branchiostoma floridae XP 002611916.1
Xenopus tropicalis XP 012816350.1
Homo sapiens NP 002487.1
Mus musculus NP 659119.2
Gallus gallus XP 001232780.2
Nematostella vectensis XP 001631383.2
Caenorhabditis elegans NP 498595.1
Drosophila melanogaster NP 524719.1
Pbil lcl|NC 031832.1 YP 009317235.1 29
Phaeodactylum tricornutum CCAP_1055/1_XP_002181917.1
Thalassiosira pseudonana_CCMP1335_XP_002293619.1
Phytophthora sojae XP 009530602.1
Aphanomyces invadans Ainv XP 008872224.1
Nannochloropsis gaditana_CCMP526_EK23157.1
Aureococcus anophagefferens XP 009033436.1
MAST4A EP00942 MAST-04A sp MAST-4A-sp1 P014850 g14850.t1
Bnat EP00466 Bigelowiella natans P008872
Ectocarpus siliculosus CBN78476.1
Taur BLSG01000444.1 9
MAST3A EP00938 MAST-03A sp MAST-3A-sp1 P007124 g7124.t1
Cyanidioschyzon merolae strain_10D_XP_005538869.1
Bmot EP00823 Brevimastigomonas motovehiculus P001412
Cvie GILI01004566.1.p1 GENE.GILI01004566.1
Blastocystis hominis XP 012893627.1
Blastocystis sp. ATCC 50177/Nand II Blasto OAO11908.1
Blastocystis sp. ATCC 50177/Nand II Blasto OAO15235.1
Vver EP00029 Vermamoeba vermiformis P007338
Vver EP00029 Vermamoeba vermiformis P007339
Atwi GFYU01046033.1 1
Paramecium tetraurelia strain_d4-2_XP_001453457.1
Tetrahymena thermophila_SB210_XP_001013096.2
Otri ARYC01001302.1 5
Nova EP00826 Nyctotherus ovalis T034217 3
Sphaeroforma arctica_JP610_XP_014154046.1
Capsaspora owczarzaki ATCC_30864_XP_004343921.1
mt Jakoba libera YP 007890680.1
Ngru lcl|NC 002573.1 NP 066510.1 13
Nfow lcl|NC 021104.1 YP 007890031.1 13
mt Pleurostomum flabellatum YP 010049266.1
mt Stachyamoeba lipophora YP 009118149.1
Akon lcl|NC 026286.1 YP 009117139.1 28
HBB2 lcl|AQL10458.1
Drot lcl|NC 029886.1 YP 009245617.1 48
EU 17 18 nad8 lcl
Maro lcl|AP019310.1 BBH42953.1 51
Pico 50635 5
Pico 65167 6
Cpar tig00020553 g10651.t1
Hand lcl|NC 010637.1 YP 001874781.1 25
mt Chroomonas placoidea YP 009476601.1
mt Cryptomonas curvata YP 009476765.1
mt Teleaulax amphioxeia YP 009475797.1
mt Proteomonas sulcata YP 009476706.1
Rsal lcl|NC 002572.1 NP 066475.1 22
mt Storeatula NC 037452.1 YP 009476643.1 7
Mvir EP00899 Mesostigma viride P018933
Emiliania huxleyi CCMP1516 Ehux XP 005769810.1
Emiliania huxleyi CCMP1516 Ehux XP 005768496.1
Emiliania huxleyi_CCMP1516_XP_005789670.1
Emiliania huxleyi CCMP1516 Ehux XP 005787161.1
Chrysochromulina tobinii Ctob KOO52783.1
Micromonas pusilla_CCMP1545_XP_003064161.1
Pwic JADZLO010000003.1 8028
Chlamydomonas reinhardtii XP 001702368.1
Ostreococcus tauri XP 022840440.1
Pycnococcus provasolii Ppro GHP04809.1
Marchantia polymorpha Mpol PTQ47285.1
Physcomitrium patens XP 024383768.1
Arabidopsis thaliana NP 178022.1
Nicotiana tabacum Ntab XP 016452428.1
Nicotiana tabacum Ntab XP 016434148.1
Nicotiana tabacum Ntab XP 016480777.1
Nicotiana tabacum Ntab XP 016439339.1
Ppur TRINITY DN1019 c0 g1 i4.p2
Ppur TRINITY DN1019 c0 g1 i11.p2
Ppur TRINITY DN1019 c0 g1 i2.p1
Chondrus crispus XP 005715136.1
Galdieria sulphuraria XP 005704887.1
Thecamonas trahens ATCC_50062_XP_013762269.1
Cvie GILI01070950.1.p2 GENE.GILI01070950.1
Dictyostelium discoideum_AX4_XP_642732.1
Pbra EP00473 Plasmodiophora brassicae P007037
TelT EP00656 Telonema subtile P033808
Mcal Malawimonas californiana 04023.mRNA.1
Reticulomyxa filosa ETO04660.1
Cafeteria roenbergensis KAA0148419.1
Hmar lcl|LC515367.1 BBQ05355.1 31
Pkir lcl|NC 034798.1 YP 009370827.1 21
Lmar lcl|NC 045933.1 YP 009730078.1 34
Nqua nad8 lcl
Andgo lcl|NC 021124.1 YP 007890505.1 41
Tglo lcl|NC 023545.1 YP 009004126.1 24
Pico 11094 4
Mjak EP00700 Malawimonas jakobiformis P003514
Mjak EP00700 Malawimonas jakobiformis P001711
mt Jakoba bahamiensis YP 007890622.1
Pame lcl|NC 001823.1 NP 044745.1 1
mt Histiona aroides YP 007890537.1
mt Seculamonas ecuadoriensis YP 007890762.1
Gnos EC124596.1.p1 GENE.EC124596.1
Gnos EC120063.1.p1 GENE.EC120063.1
Gnos EC119462.1.p1 GENE.EC119462.1
bacterial NADH-quinone oxidoreductase subunit I (9)
Komagataeibacter hansenii WP 003620721.1
Roseomonas cervicalis WP 007002789.1
Magnetospirillum WP 008617495.1 MLTISPECIES
Reyranella massiliensis WP 020696097.1
bacterial NADH-quinone oxidoreductase subunit I (2)
bacterial NADH-quinone oxidoreductase subunit I (2)
Sneathiella glossodoripedis WP 234397701.1
Trypanosoma brucei lcl|AAA91499.1
Dpap lcl|APQ44789.1
Candidatus Puniceispirillum marinum WP 148212320.1
Candidatus Endolissoclinum faulkneri WP 015088284.1
bacterial NADH-quinone oxidoreductase subunit I (15)
bacterial NADH-quinone oxidoreductase subunit I (15)
Magnetococcus marinus WP 011715166.1
Deferribacter desulfuricans WP 013008673.1
bacterial NADH-quinone oxidoreductase subunit I (5)
bacterial NADH-quinone oxidoreductase subunit I (16)
bacterial NADH-quinone oxidoreductase subunit I (2)
bacterial NADH-quinone oxidoreductase subunit I (4)
Photobacterium profundum WP 036802631.1
Nicotiana tabacum Ntab NP 054561.1
Marchantia polymorpha Mpol PTQ38921.1
Mvir EP00899 Mesostigma viride P012289
Acaryochloris marina WP 012162652.1
Crinalium epipsammum WP 198013683.1
Anabaena cylindrica WP 015212762.1
Stanieria cyanosphaera WP 015192132.1
bateria 4Fe-4S binding proteins (7)
bacterial 4Fe-4S binding protein (5)
bacterial 4Fe-4S binding proteins (4)
Tumebacillus flagellatus WP 038087442.1
Nautilia profundicola WP 015902393.1
bacterial formate hydrogenlyase complex iron-sulfur protein (13)
Nautilia profundicola WP 015902118.1
Magnetococcus marinus WP 011713827.1
bacterial 4Fe-4S binding proteins (8)
bacterial 4Fe-4S binding proteins (6)
bacterial oxidoreductase family proteins (4)
Ilyobacter polytropus WP 013386485.1
Deferribacter desulfuricans WP 013007234.1
bacterial 4Fe-4S binding protein (4)
Desulfatibacillum alkenivorans WP 073473145.1
Desulfatibacillum alkenivorans WP 073478467.1
bacterial 4Fe-4S binding protein (3)
bacterial ferredoxin family proteins (10)
Protein: nad8; alignment timming: ClipKIT; IQ-TREE2 best-fit model: VT+R8
0.50

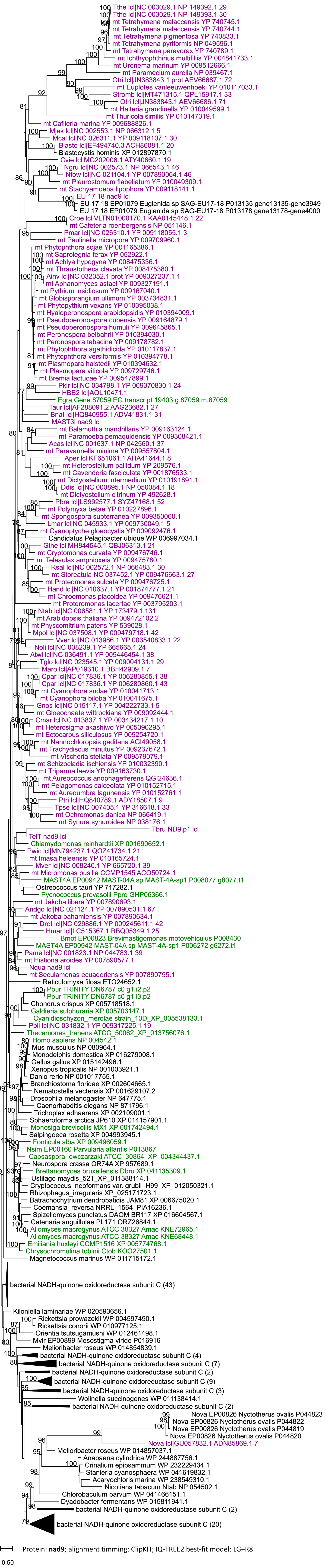

Protein: **nad9**; alignment timming: ClipKIT; IQ-TREE2 best-fit model: LG+R8

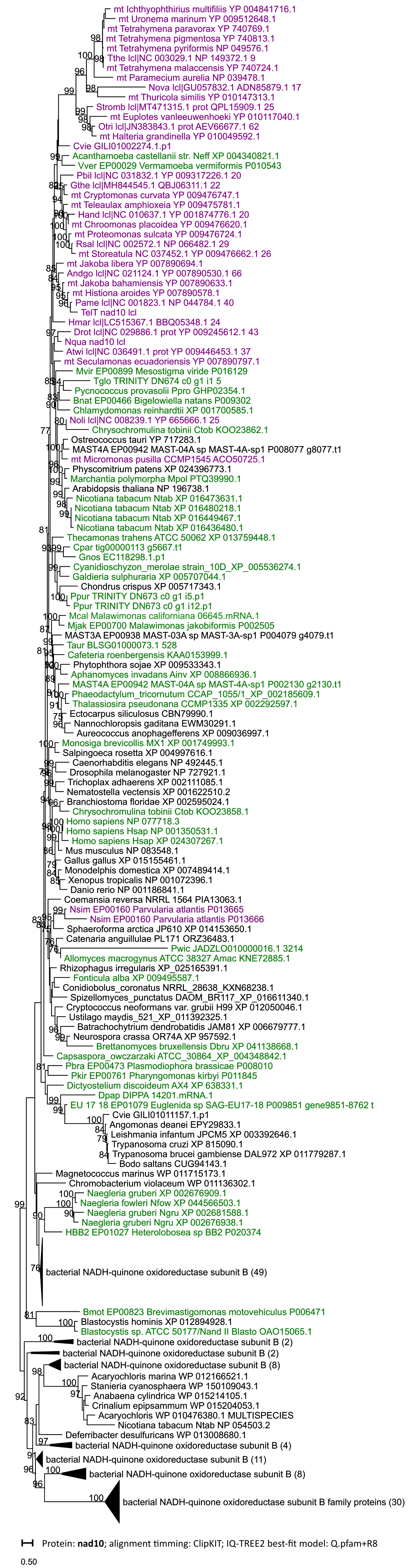

Protein: **nad10**; alignment timming: ClipKIT; IQ-TREE2 best-fit model: Q.pfam+R8

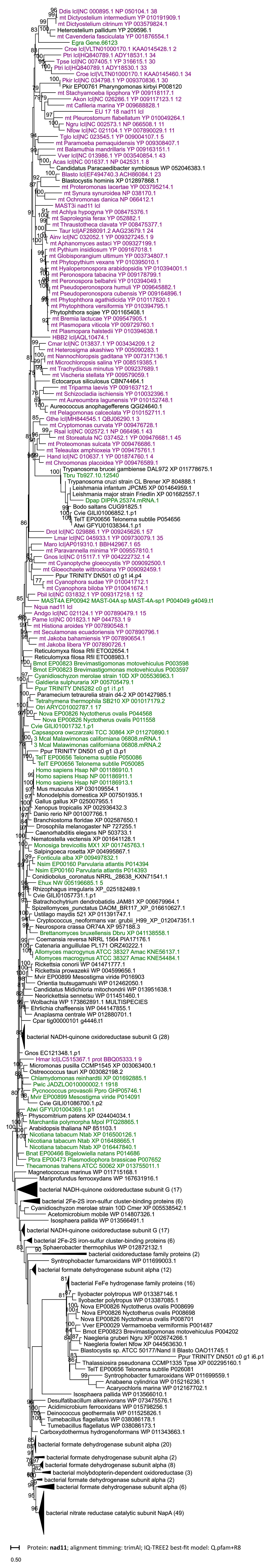

Protein: **nad11**; alignment timming: trimAl; IQ-TREE2 best-fit model: Q.pfam+R8

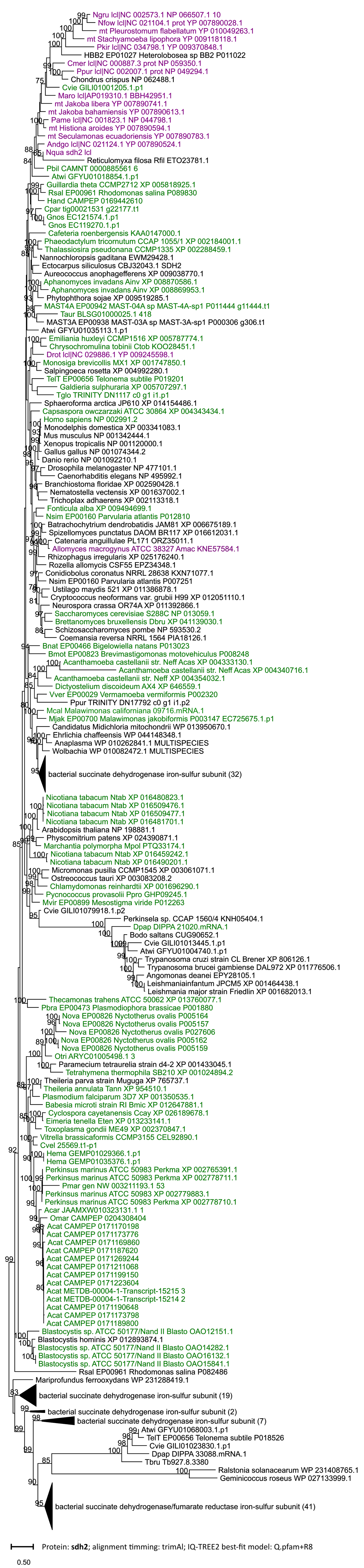

Protein: **sdh2**; alignment timming: trimAl; IQ-TREE2 best-fit model: Q.pfam+R8

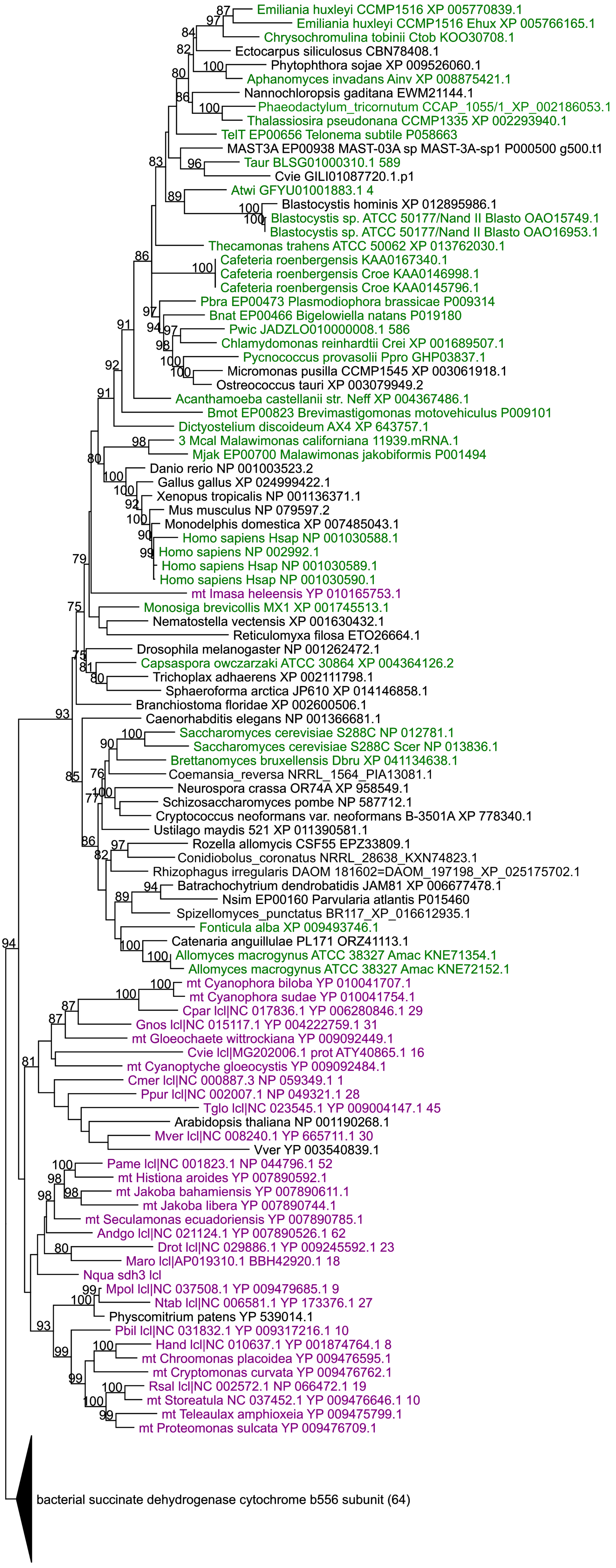

Protein: **sdh3**; alignment timming: ClipKIT; IQ-TREE2 best-fit model: VT+F+R5

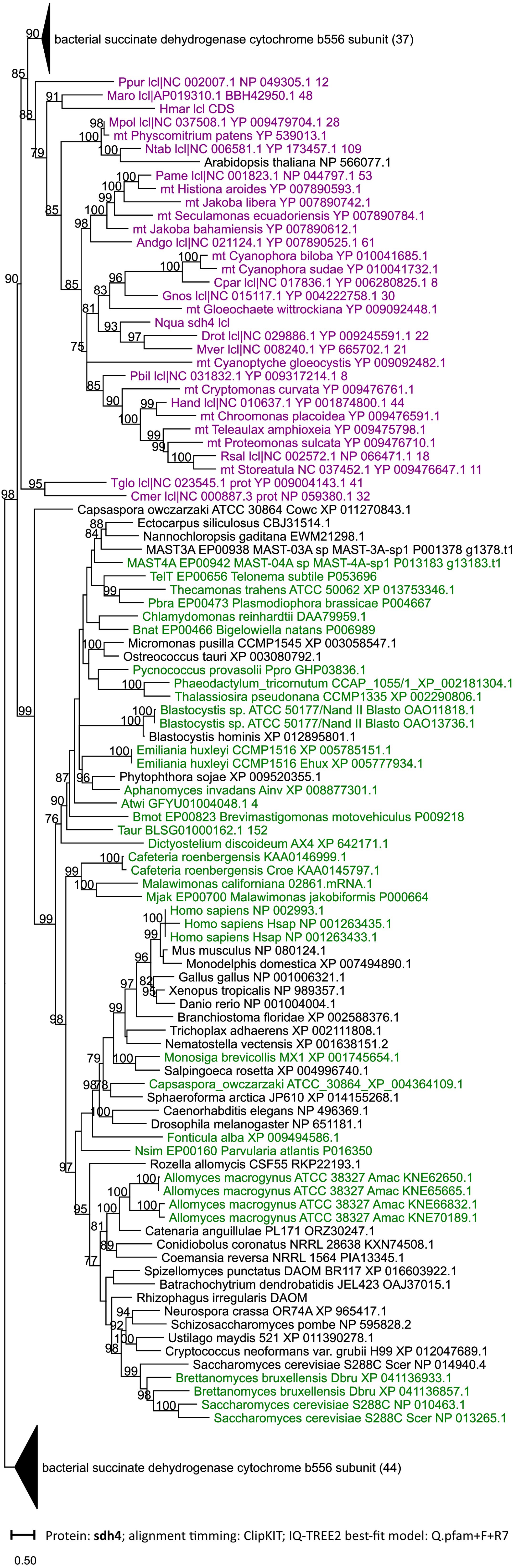

Protein: **sdh4**; alignment timming: ClipKIT; IQ-TREE2 best-fit model: Q.pfam+F+R7

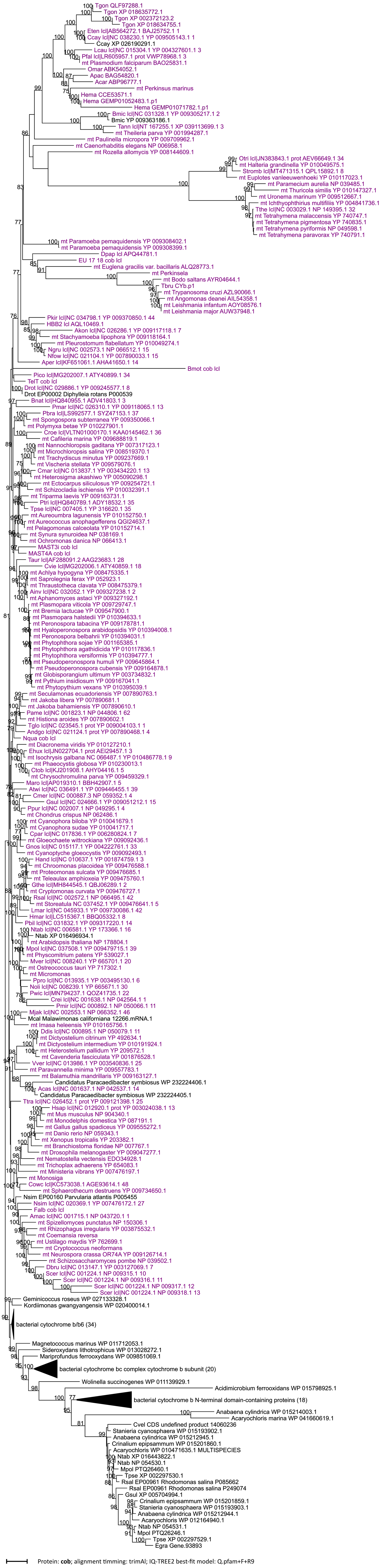

Protein: **cob**; alignment timming: trimAl; IQ-TREE2 best-fit model: Q.pfam+F+R9

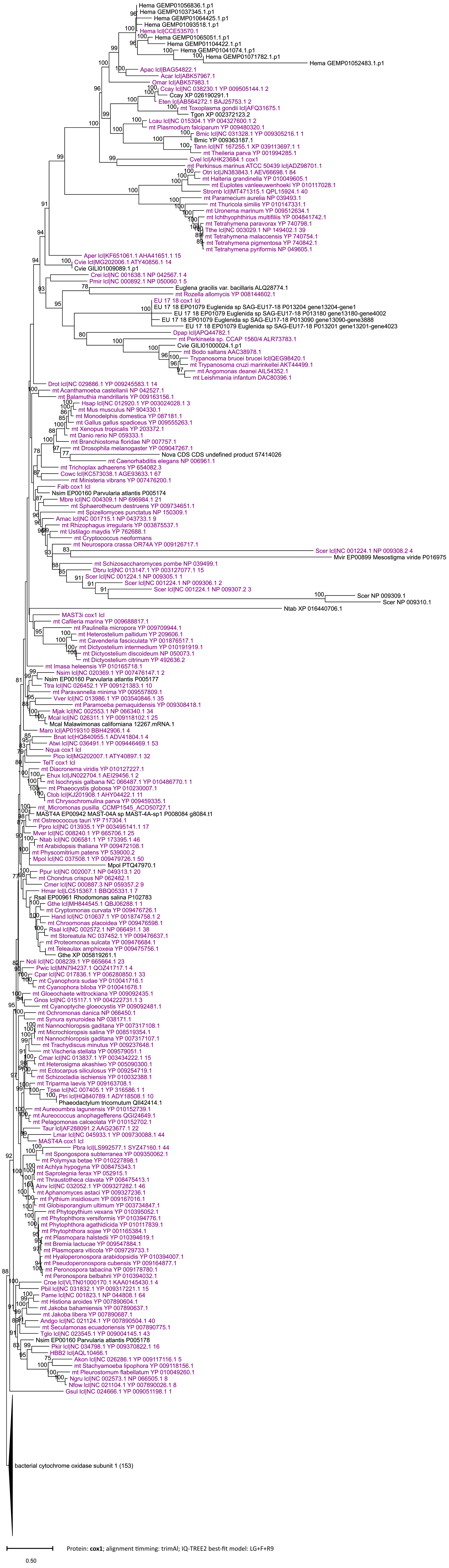

Protein: **cox1**; alignment timming: trimAl; IQ-TREE2 best-fit model: LG+F+R9

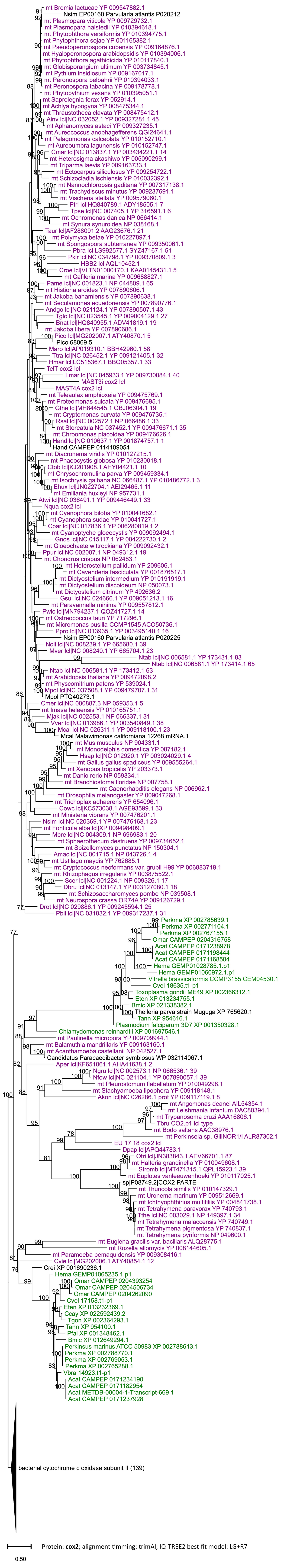

Protein: **cox2**; alignment timming: trimAl; IQ-TREE2 best-fit model: LG+R7

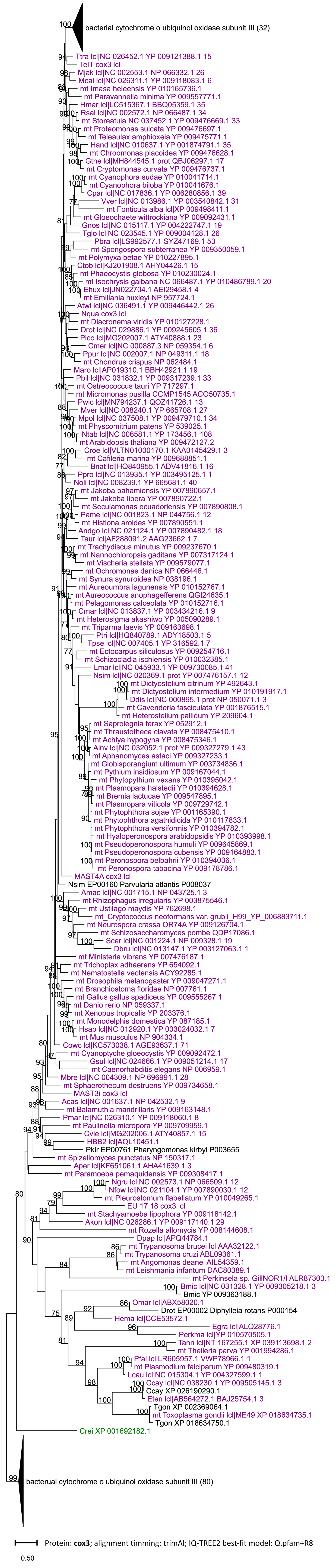
bacterial cytochrome o ubiquinol oxidase subunit III (32)
Ttra lcl|NC 026452.1 YP 009121388.1 15
TelT cox3 lcl
Mjak lcl|NC 002553.1 NP 066332.1 26
Mcal lcl|NC 026311.1 YP 009118083.1 6
mt Imasa heleensis YP 010165736.1
mt Paravannella minima YP 009557771.1
Hmar lcl|LC515367.1 BBQ05359.1 35
Rsal lcl|NC 002572.1 NP 066487.1 34
mt Storeatula NC 037452.1 YP 009476669.1 33
mt Proteomonas sulcata YP 009476697.1
mt Teleaulax amphioxeia YP 009475771.1
Hand lcl|NC 010637.1 YP 001874791.1 35
mt Chroomonas placoidea YP 009476628.1
Gthe lcl|MH844545.1 prot QBJ06297.1 17
mt Cryptomonas curvata YP 009476737.1
mt Cyanophora sudae YP 010041714.1
mt Cyanophora biloba YP 010041676.1
Cpar lcl|NC 017836.1 YP 006280856.1 39
Vver lcl|NC 013986.1 YP 003540842.1 31
mt Fonticula alba lcl|XP 009498411.1
mt Gloeochaete wittrockiana YP 009092431.1
Gnos lcl|NC 015117.1 YP 004222747.1 19
Tglo lcl|NC 023545.1 YP 009004128.1 26
Pbra lcl|LS992577.1 SYZ47169.1 53
mt Spongospora subterranea YP 009350059.1
mt Polymyxa betae YP 010227895.1
Ctob lcl|KJ201908.1 AHY04426.1 15
mt Phaeocystis globosa YP 010230024.1
mt Isochrysis galbana NC 066487.1 YP 010486789.1 20
Ehux lcl|JN022704.1 AEI29458.1 4
mt Emiliania huxleyi NP 957724.1
Atwi lcl|NC 036491.1 YP 009446442.1 26
Nqua cox3 lcl
mt Diacronema viridis YP 010127228.1
Drot lcl|NC 029886.1 YP 009245605.1 36
Pico lcl|MG202007.1 ATY40888.1 23
Cmer lcl|NC 000887.3 NP 059354.1 6
Ppur lcl|NC 002007.1 NP 049311.1 18
mt Chondrus crispus NP 062484.1
Maro lcl|AP019310.1 BBH42921.1 19
Pbil lcl|NC 031832.1 YP 009317239.1 33
mt Ostreococcus tauri YP 717297.1
mt Micromonas pusilla CCMP1545 ACO50735.1
Pwic lcl|MN794237.1 QOZ41726.1 13
Mver lcl|NC 008240.1 YP 665708.1 27
Mpol lcl|NC 037508.1 YP 009479710.1 34
mt Physcomitrium patens YP 539025.1
Ntab lcl|NC 006581.1 YP 173456.1 108
mt Arabidopsis thaliana YP 009472127.2
Croe lcl|VLTN01000170.1 KAA0145429.1 3
mt Cafileria marina YP 009688851.1
Bnat lcl|HQ840955.1 ADV41816.1 16
Ppro lcl|NC 013935.1 YP 003495125.1 1
Noli lcl|NC 008239.1 YP 665681.1 40
mt Jakoba bahamiensis YP 007890657.1
mt Jakoba libera YP 007890722.1
mt Seculamonas ecuadoriensis YP 007890808.1
Pame lcl|NC 001823.1 NP 044756.1 12
mt Histiona aroides YP 007890551.1
Andgo lcl|NC 021124.1 YP 007890482.1 18
Taur lcl|AF288091.2 AAG23662.1 7
mt Trachydiscus minutus YP 009237670.1
mt Nannochloropsis gaditana YP 007317124.1
mt Vischeria stellata YP 009579077.1
mt Ochromonas danica NP 066446.1
mt Synura synuroidea NP 038196.1
mt Aureoumbra lagunensis YP 010152767.1
mt Aureococcus anophagefferens QGI24635.1
mt Pelagomonas calceolata YP 010152716.1
Cmar lcl|NC 013837.1 YP 003434216.1 9
mt Heterosigma akashiwo YP 005090289.1
mt Triparma laevis YP 009163698.1
Ptri lcl|HQ840789.1 ADY18503.1 5
Tpse lcl|NC 007405.1 YP 316592.1 7
mt Ectocarpus siliculosus YP 009254716.1
mt Schizocladia ischiensis YP 010032385.1
Lmar lcl|NC 045933.1 YP 009730085.1 41
Nsim lcl|NC 020369.1 prot YP 007476157.1 12
mt Dictyostelium citrinum YP 492643.1
mt Dictyostelium intermedium YP 010191917.1
Ddis lcl|NC 000895.1 prot NP 050071.1 3
mt Cavenderia fasciculata YP 001876515.1
mt Heterostelium pallidum YP 209604.1
mt Saprolegnia ferax YP 052912.1
mt Thraustotheca clavata YP 008475410.1
mt Achlya hypogyna YP 008475346.1
Ainv lcl|NC 032052.1 prot YP 009327279.1 43
mt Aphanomyces astaci YP 009327233.1
mt Globisporangium ultimum YP 003734836.1
mt Pythium insidiosum YP 009167044.1
mt Phytopythium vexans YP 010395042.1
mt Plasmopara halstedii YP 010394628.1
mt Bremia lactucae YP 009547895.1
mt Plasmopara viticola YP 009729742.1
mt Phytophthora sojae YP 001165390.1
mt Phytophthora agathidicida YP 010117833.1
mt Phytophthora versiformis YP 010394782.1
mt Hyaloperonospora arabidopsidis YP 010393998.1
mt Pseudoperonospora humuli YP 009645869.1
mt Pseudoperonospora cubensis YP 009164883.1
mt Peronospora belbahrii YP 010394036.1
mt Peronospora tabacina YP 009178786.1
MAST4A cox3 lcl
Nsim EP00160 Parvularia atlantis P008037
Amac lcl|NC 001715.1 NP 043725.1 3
mt Rhizophagus irregularis YP 003875546.1
mt Ustilago maydis YP 762698.1
mt_Cryptococcus neoformans var. grubii_H99_YP_006883711.1
mt Neurospora crassa OR74A YP 009126704.1
mt Schizosaccharomyces pombe QDP17086.1
Scer lcl|NC 001224.1 NP 009328.1 19
Dbru lcl|NC 013147.1 YP 003127063.1 1
mt Ministeria vibrans YP 007476187.1
mt Trichoplax adhaerens YP 654092.1
mt Nematostella vectensis ACY92285.1
mt Drosophila melanogaster YP 009047271.1
mt Branchiostoma floridae NP 007761.1
mt Gallus gallus spadiceus YP 009555267.1
mt Danio rerio NP 059337.1
mt Xenopus tropicalis YP 203376.1
mt Monodelphis domestica YP 087185.1
Hsap lcl|NC 012920.1 YP 003024032.1 7
mt Mus musculus NP 904334.1
Cowc lcl|KC573038.1 AGE93637.1 71
mt Cyanoptyche gloeocystis YP 009092472.1
Gsul lcl|NC 024666.1 YP 009051214.1 17
mt Caenorhabditis elegans NP 006959.1
Mbre lcl|NC 004309.1 NP 696991.1 28
mt Sphaerothecum destruens YP 009734658.1
MAST3i cox3 lcl
Acas lcl|NC 001637.1 NP 042532.1 9
mt Balamuthia mandrillaris YP 009163148.1
Pmar lcl|NC 026310.1 YP 009118060.1 8
mt Paulinella micropora YP 009709959.1
Cvie lcl|MG202006.1 ATY40857.1 15
HBB2 lcl|AQL10451.1
Pkir EP00761 Pharyngomonas kirbyi P003655
mt Spizellomyces punctatus NP 150317.1
Aper lcl|KF651061.1 AHA41639.1 3
mt Paramoeba pemaquidensis YP 009308417.1
Ngru lcl|NC 002573.1 NP 066509.1 12
Nfow lcl|NC 021104.1 YP 007890030.1 12
mt Pleurostomum flabellatum YP 010049265.1
EU 17 18 cox3 lcl
mt Stachyamoeba lipophora YP 009118142.1
Akon lcl|NC 026286.1 YP 009117140.1 29
mt Rozella allomycis YP 008144608.1
Dpap lcl|APQ44784.1
mt Trypanosoma brucei lcl|AAA32122.1
mt Trypanosoma cruzi ABL09361.1
mt Angomonas deanei AIL54359.1
mt Leishmania infantum DAC80389.1
mt Perkinsela sp. GillNOR1/I ALR87303.1
Bmic lcl|NC 031328.1 YP 009305218.1 3
Bmic YP 009363188.1
Omar lcl|ABX58020.1
Drot EP00002 Diphylleia rotans P000154
Hema lcl|CCE53572.1
Egra lcl|ALQ28776.1
Perkma lcl|YP 010570505.1
Tann lcl|NT 167255.1 XP 039113698.1 2
mt Theileria parva YP 001994286.1
Pfal lcl|LR605957.1 VWP78966.1 1
mt Plasmodium falciparum YP 009480319.1
Lcau lcl|NC 015304.1 YP 004327599.1 1
Ccay lcl|NC 038230.1 YP 009505145.1 3
Ccay XP 026190290.1
Eten lcl|AB564272.1 BAJ25754.1 3
Tgon XP 002369064.1
mt Toxoplasma gondii lcl|ME49 XP 018634735.1
Tgon XP 018634750.1
Crei XP 001692182.1
bacterual cytochrome o ubiquinol oxidase subunit III (80)
Protein: cox3; alignment timming: trimAl; IQ-TREE2 best-fit model: Q.pfam+R8
0.50

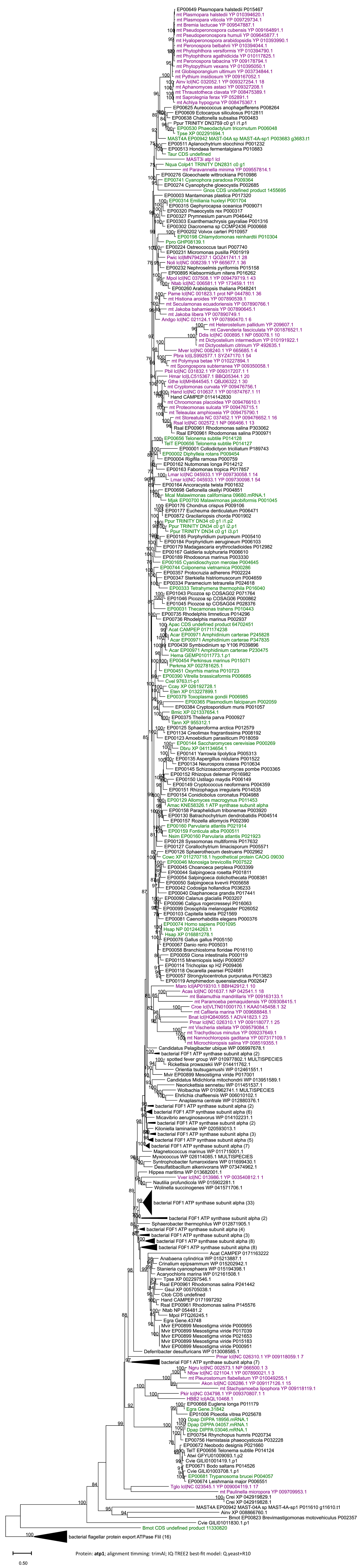

Protein: **atp1**; alignment timming: trimAl; IQ-TREE2 best-fit model: Q.yeast+R10

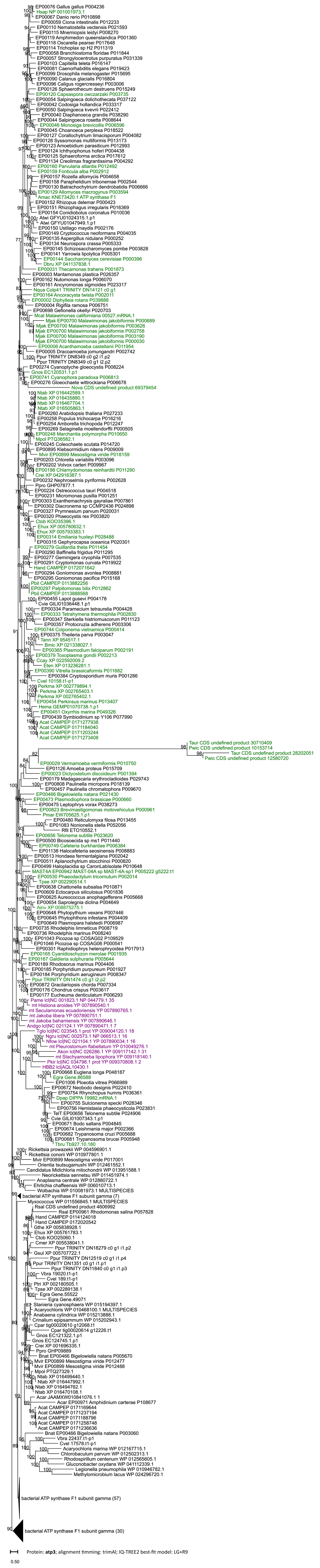

Protein: **atp3**; alignment timming: trimAl; IQ-TREE2 best-fit model: LG+R9

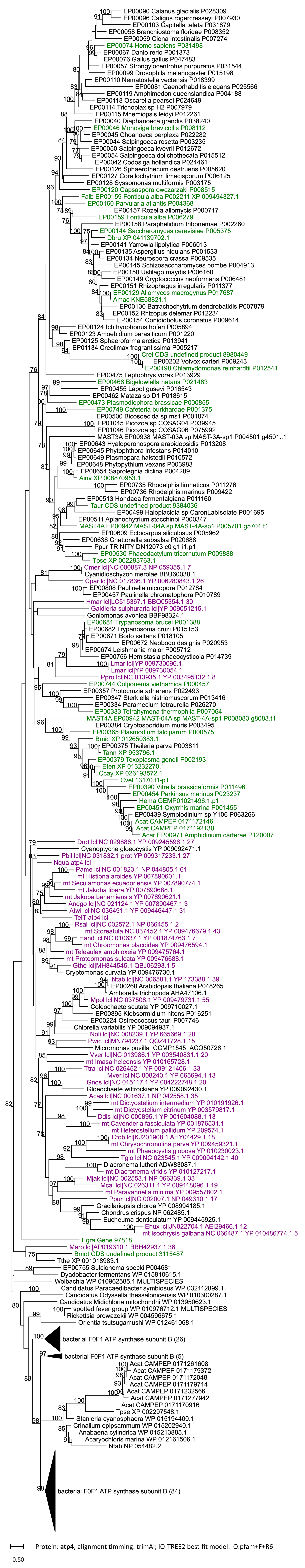

Protein: **atp4**; alignment timming: trimAl; IQ-TREE2 best-fit model: Q.pfam+F+R6

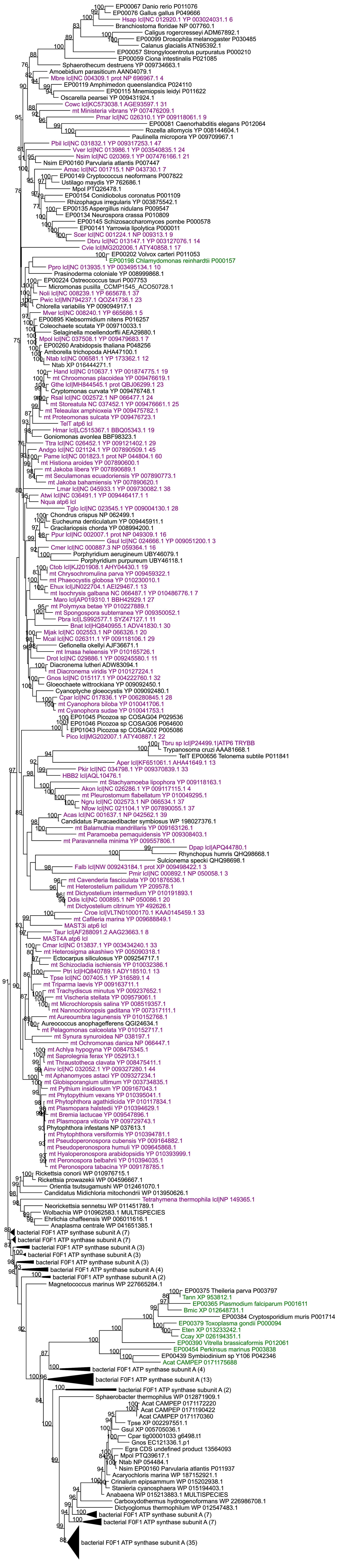

Protein: **atp6**; alignment timming: trimAl; IQ-TREE2 best-fit model: LG+F+R9

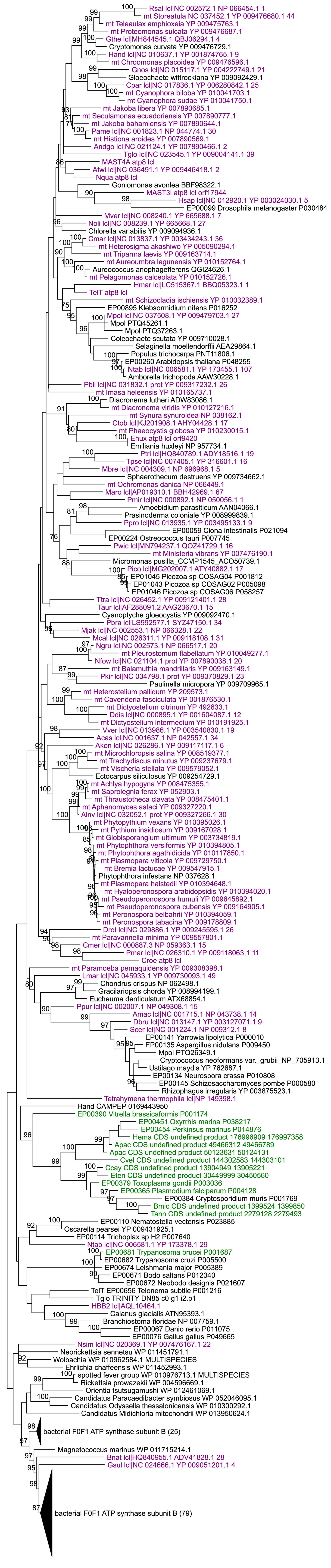

Protein: **atp8**; alignment timming: ClipKIT; IQ-TREE2 best-fit model: Q.pfam+F+R6

0.50

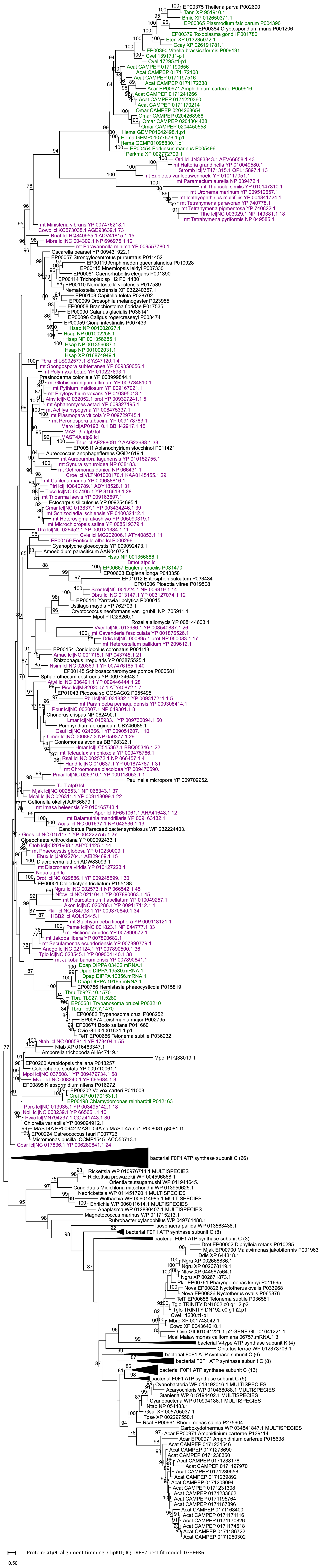

Protein: **atp9**; alignment timming: ClipKIT; IQ-TREE2 best-fit model: LG+F+R6

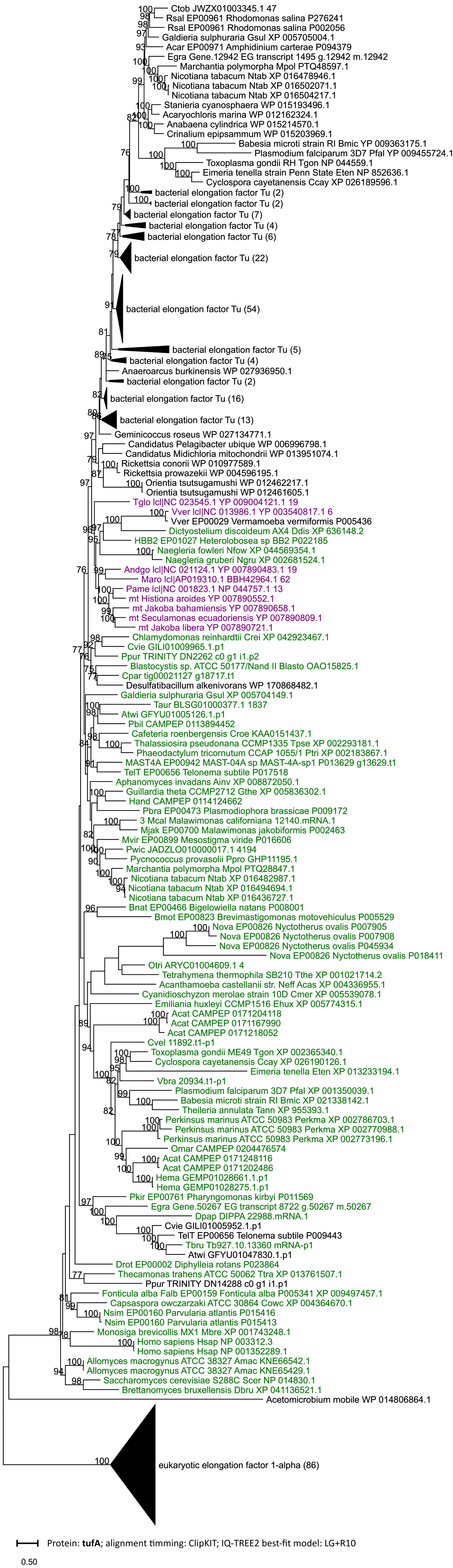

Protein: **tufA**; alignment timming: ClipKIT; IQ-TREE2 best-fit model: LG+R10

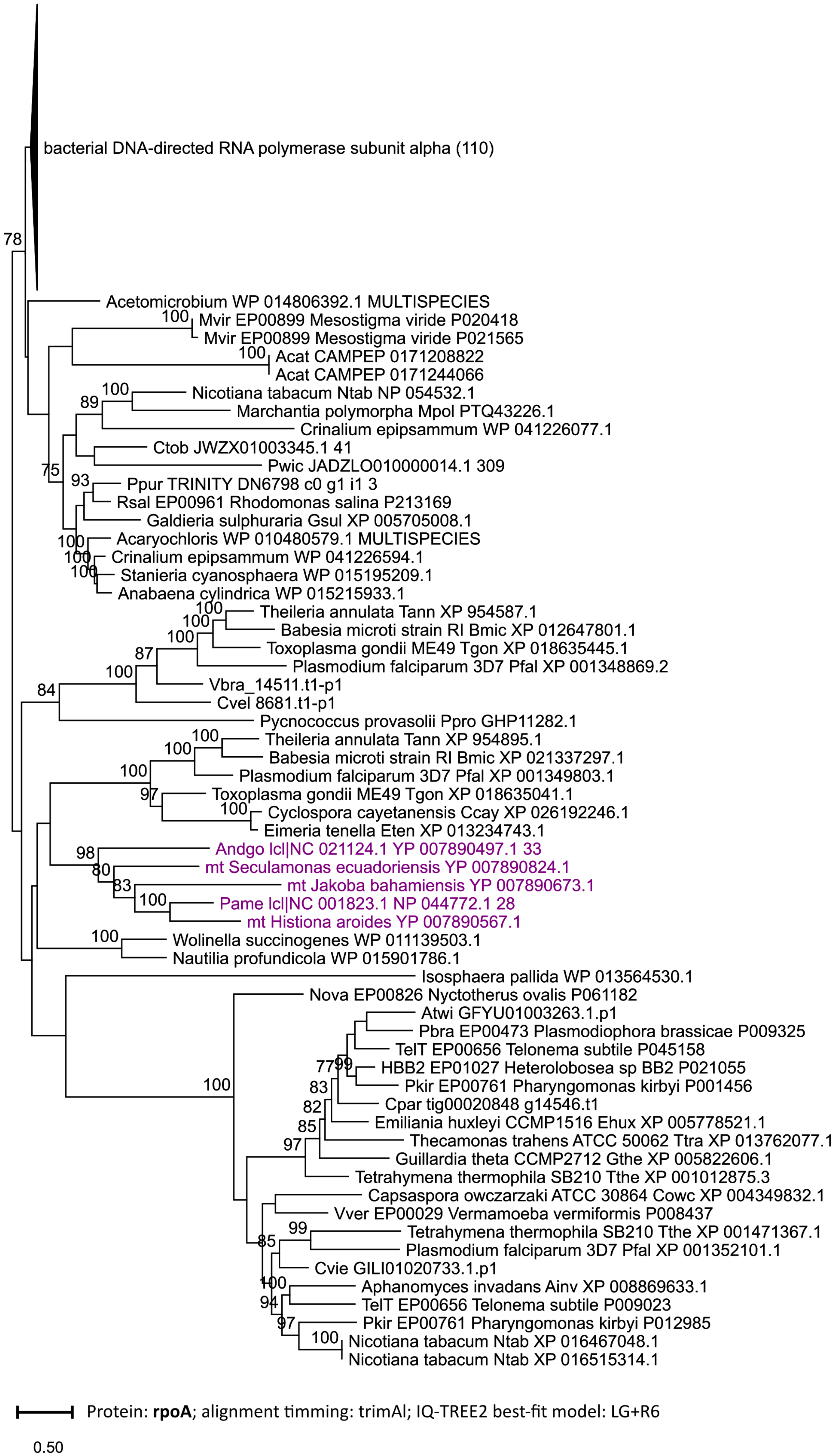

Protein: **rpoA**; alignment timming: trimAl; IQ-TREE2 best-fit model: LG+R6

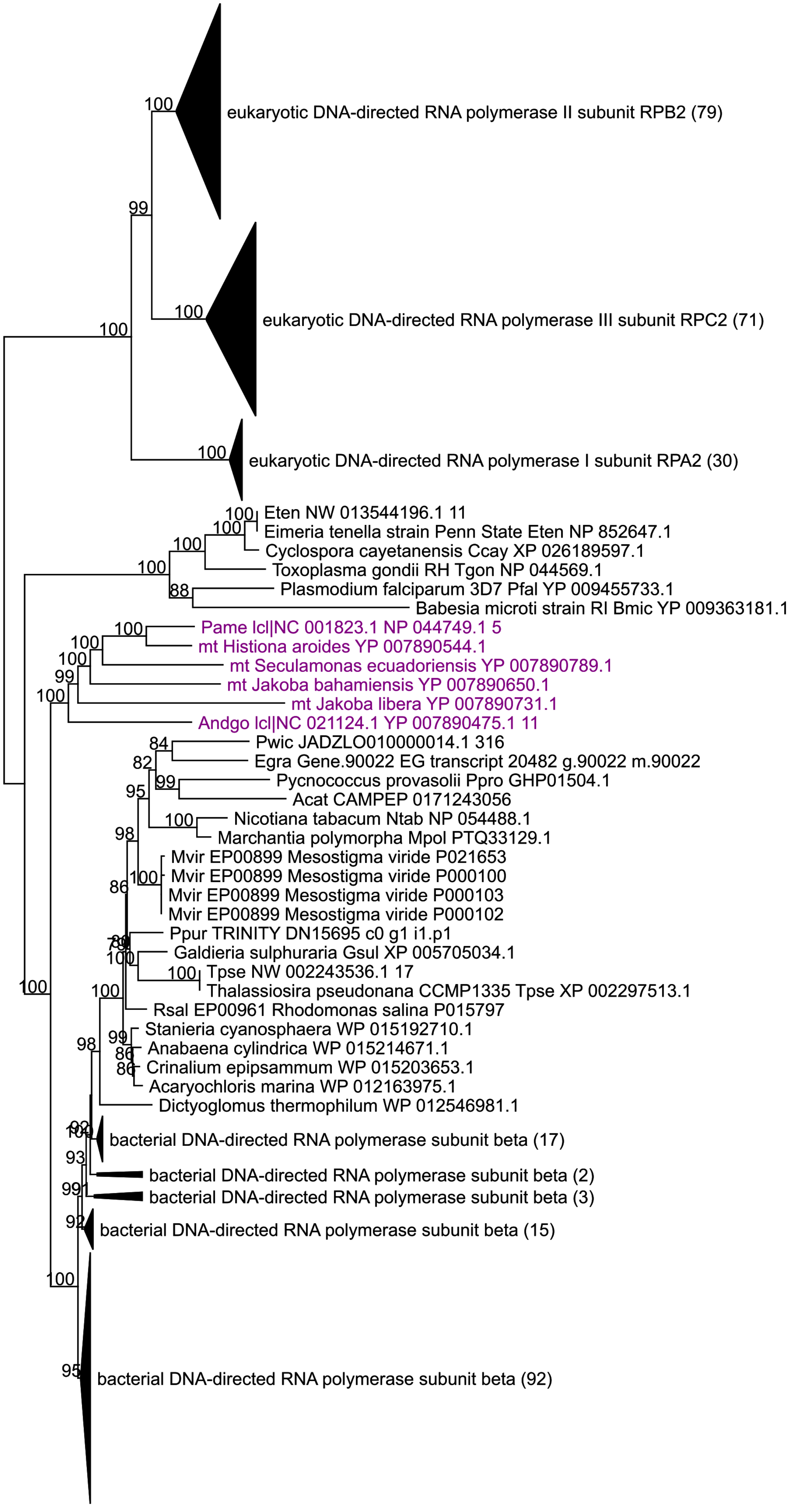

Protein: **rpoB**; alignment timming: ClipKIT; IQ-TREE2 best-fit model: LG+R10

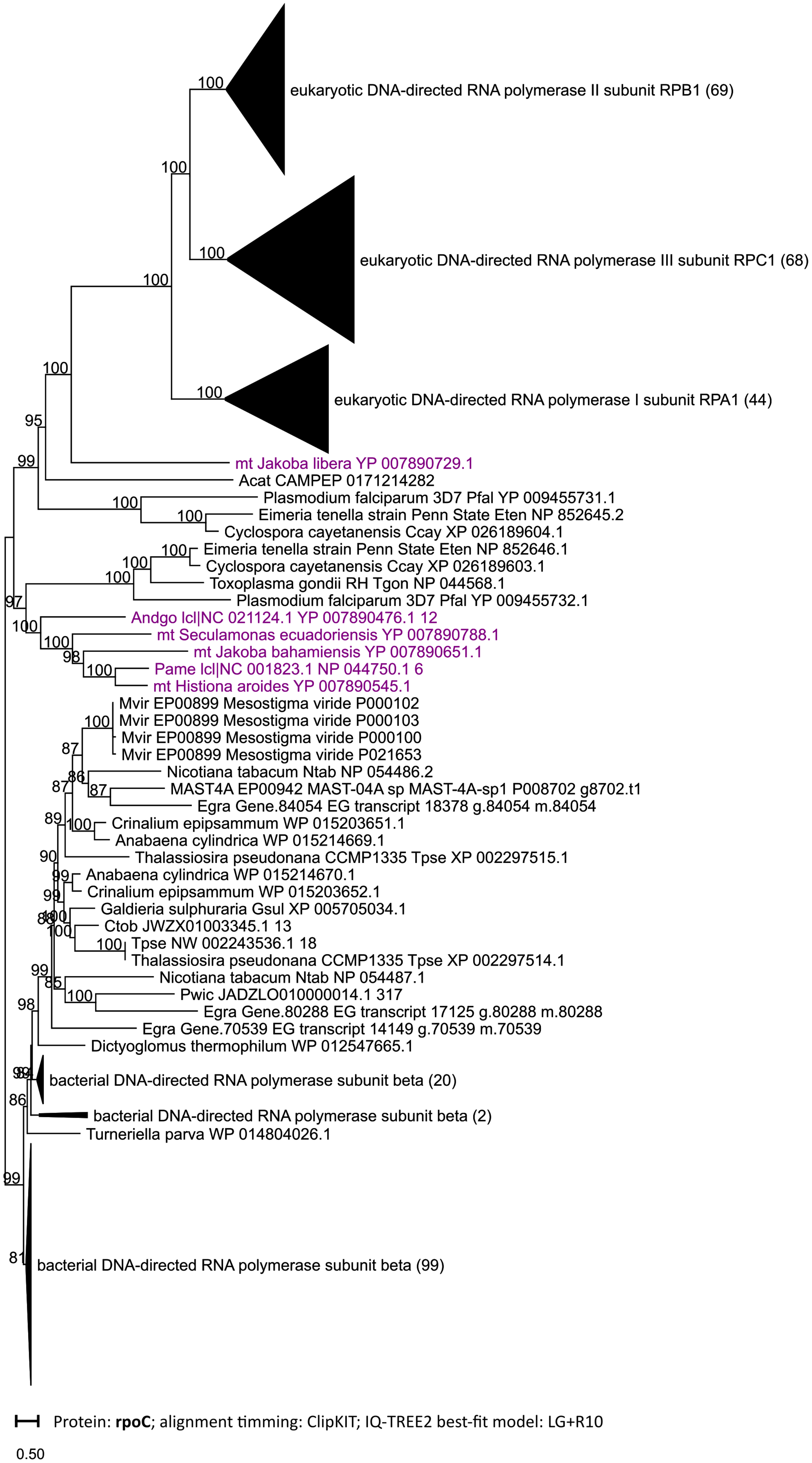

Protein: **rpoC**; alignment timming: ClipKIT; IQ-TREE2 best-fit model: LG+R10

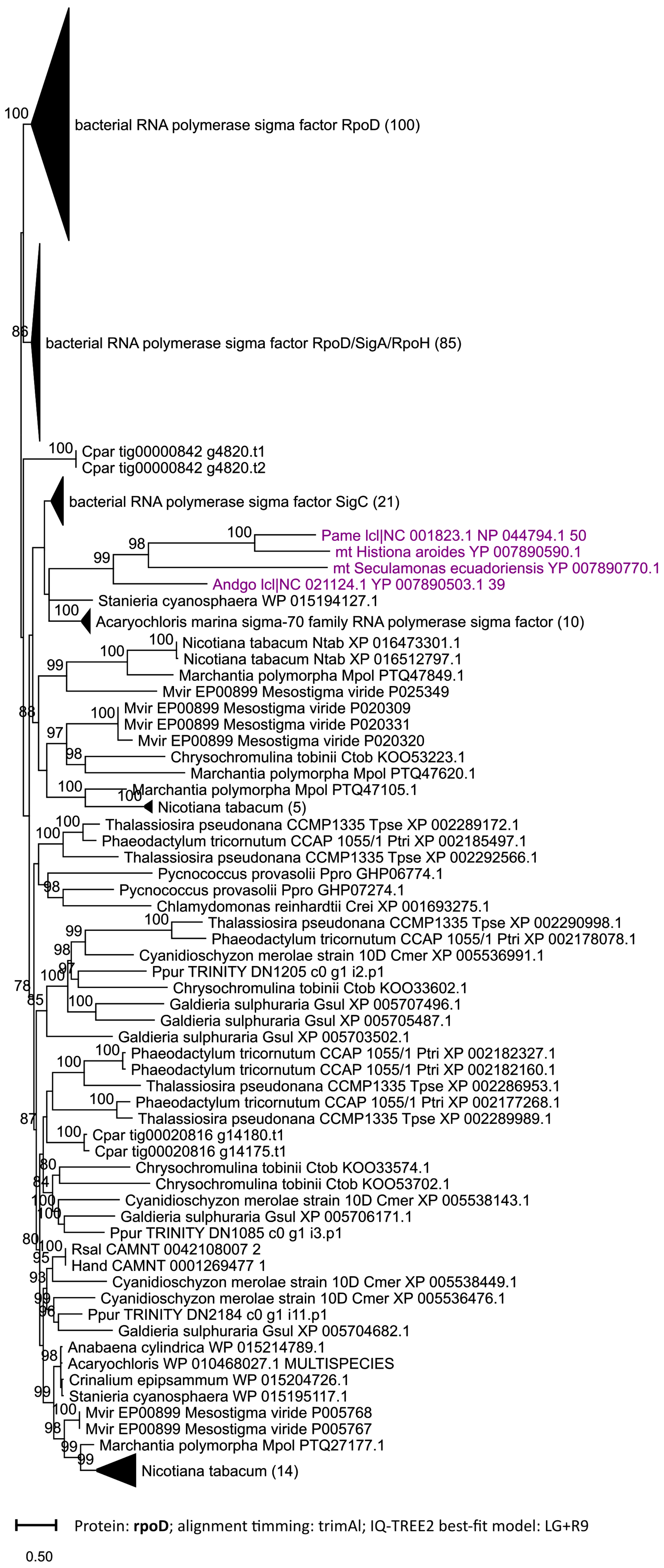

Protein: **rpoD**; alignment timming: trimAl; IQ-TREE2 best-fit model: LG+R9

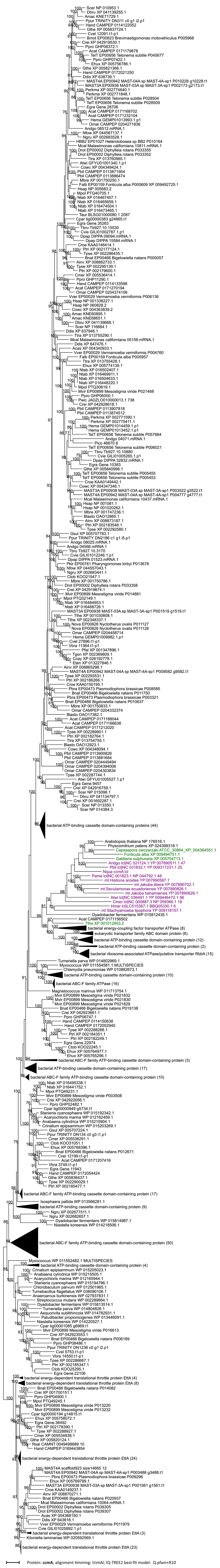

Protein: **ccmA**; alignment timming: trimAl; IQ-TREE2 best-fit model: Q.pfam+R10

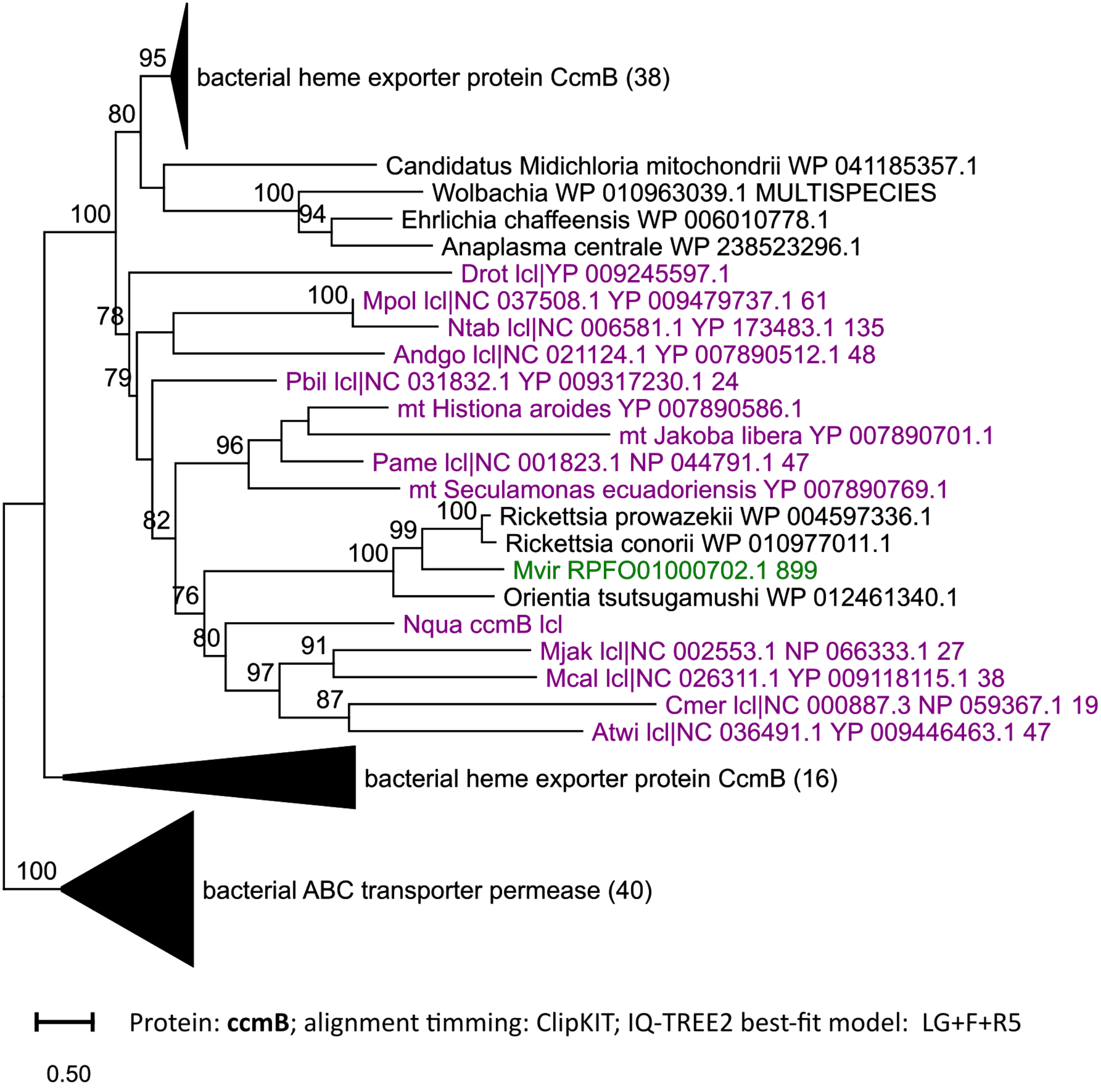

Protein: **ccmB**; alignment timming: ClipKIT; IQ-TREE2 best-fit model: LG+F+R5

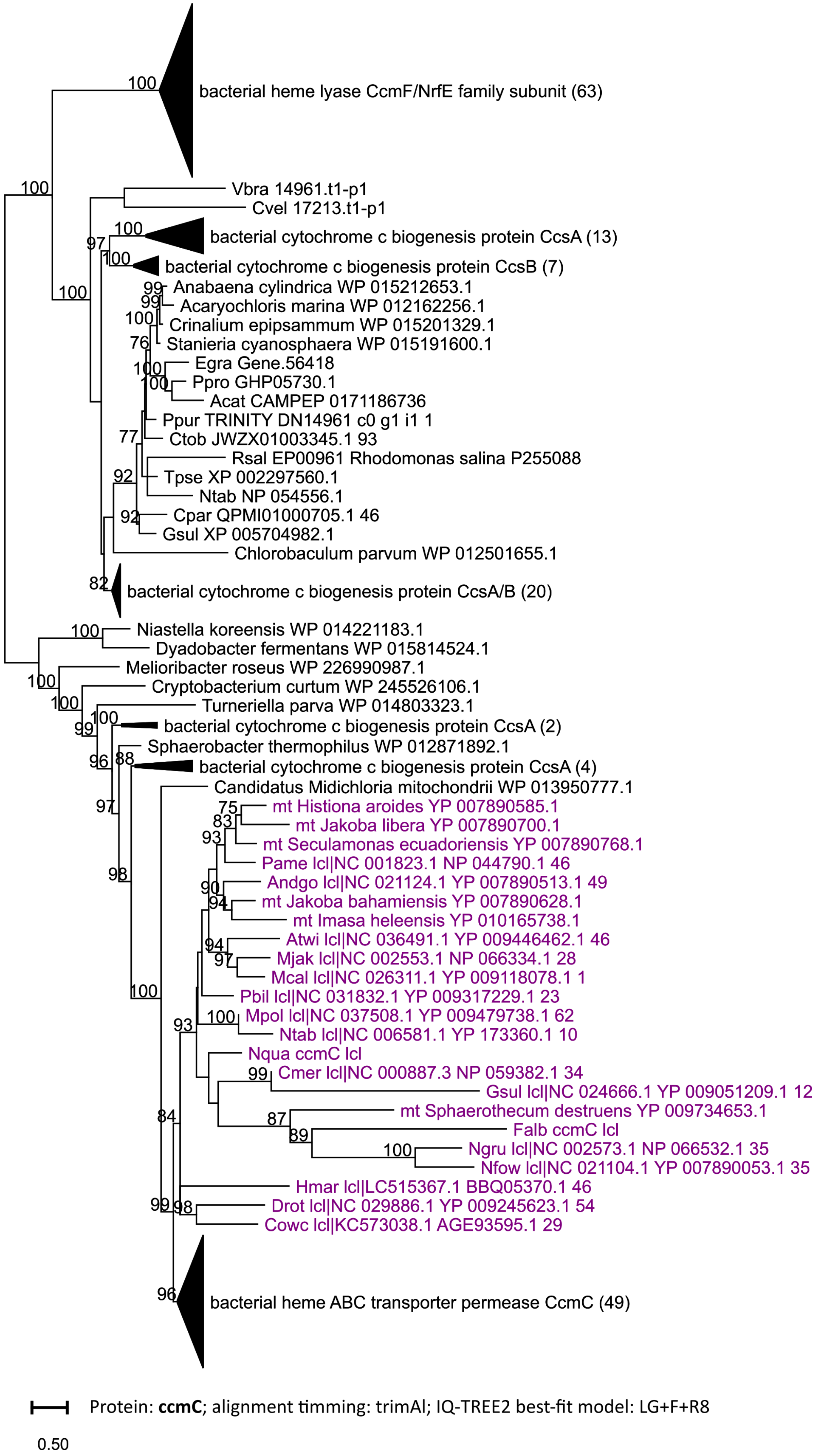

Protein: **ccmC**; alignment timming: trimAl; IQ-TREE2 best-fit model: LG+F+R8

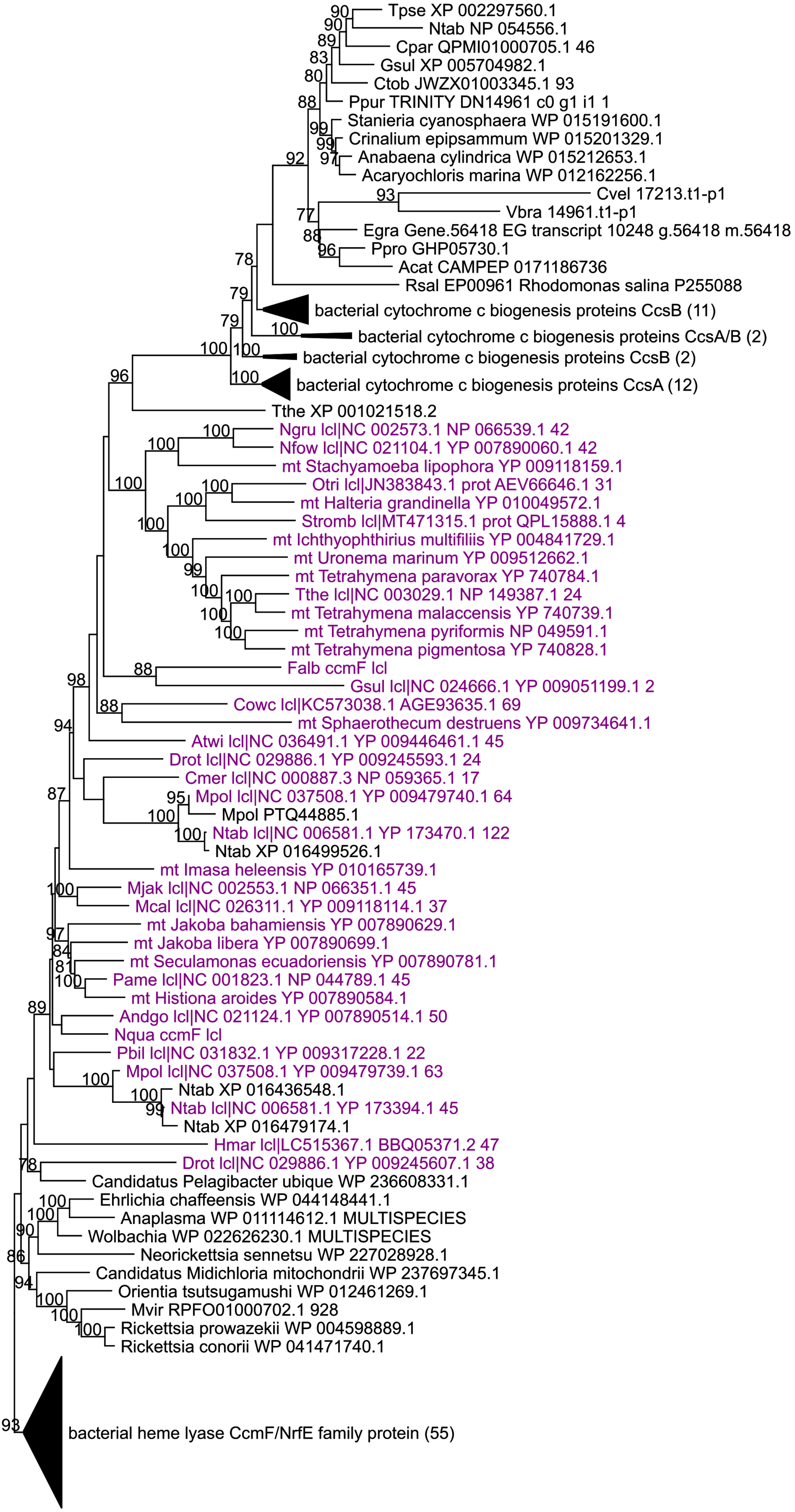

Protein: **ccmF**; alignment timming: ClipKIT; IQ-TREE2 best-fit model: Q.pfam+F+R6

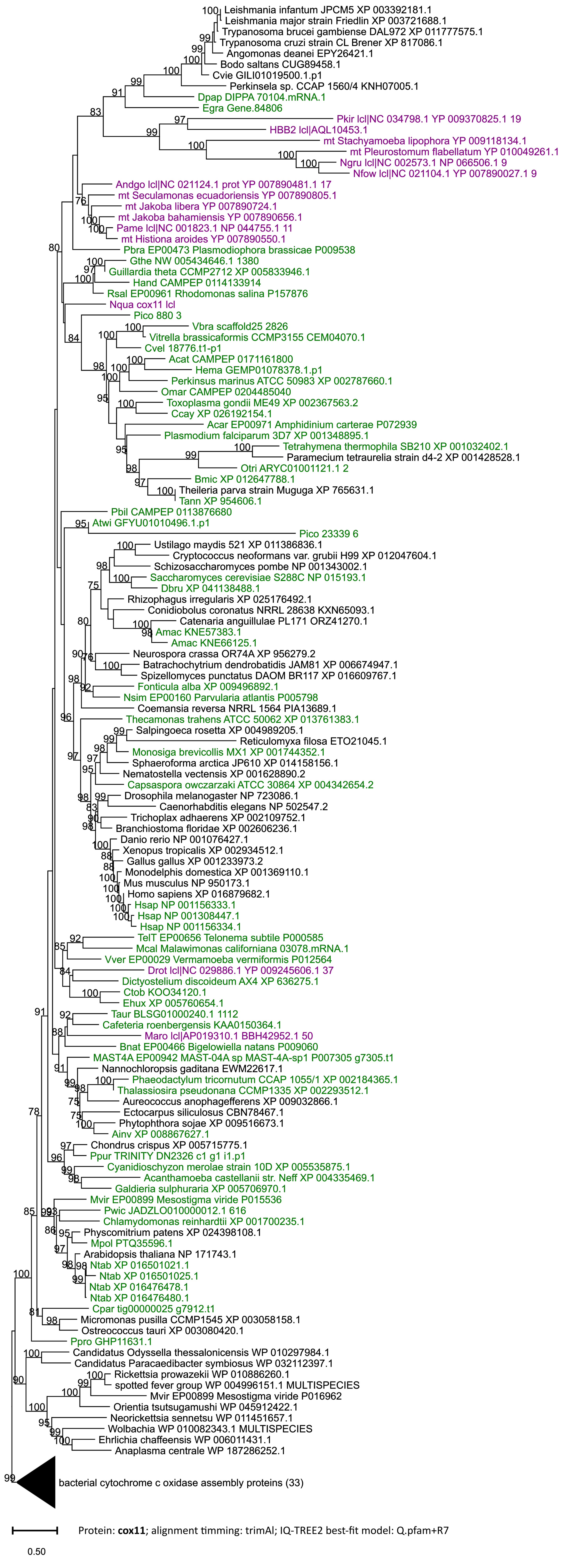

Protein: **cox11**; alignment timming: trimAl; IQ-TREE2 best-fit model: Q.pfam+R7

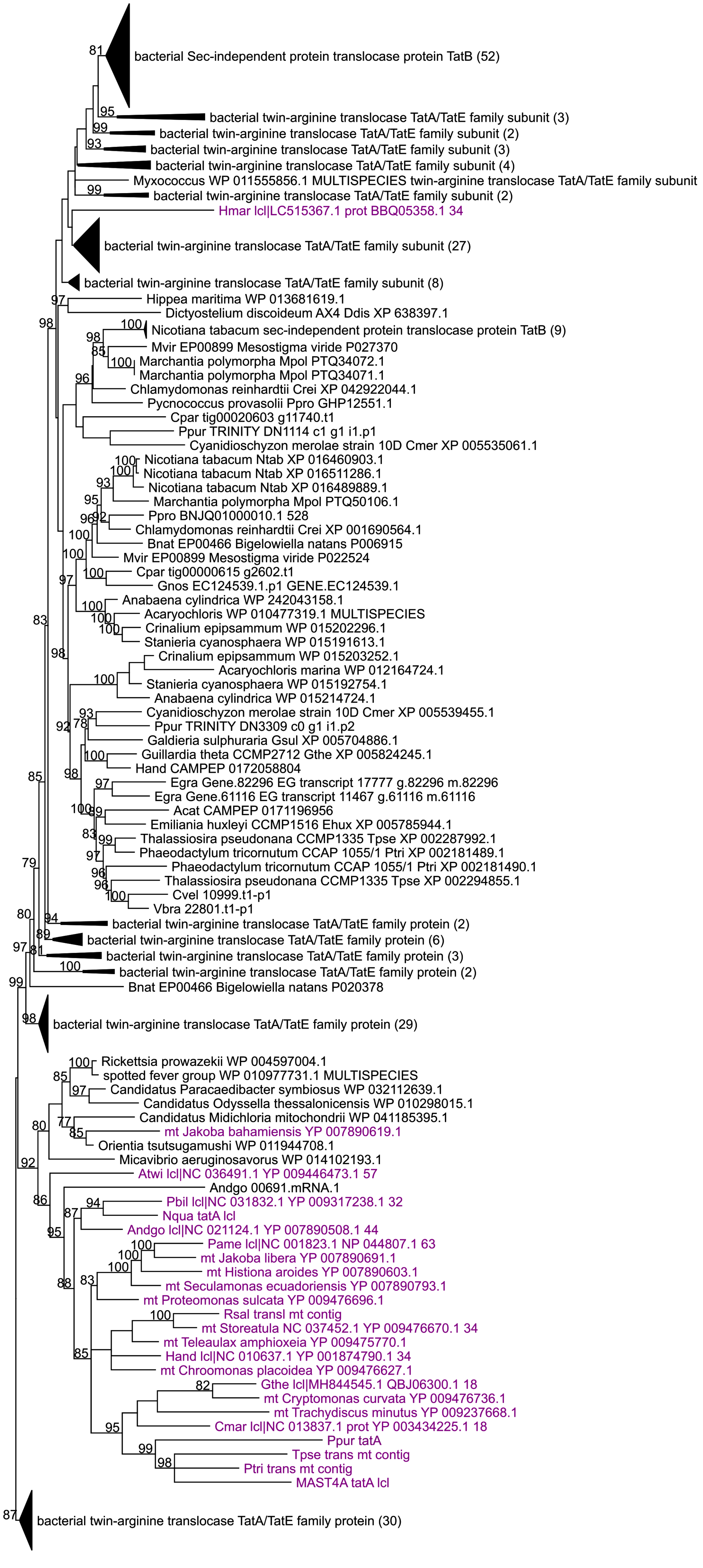

Protein: **tatA**; alignment timming: ClipKIT; IQ-TREE2 best-fit model: Q.pfam+R5

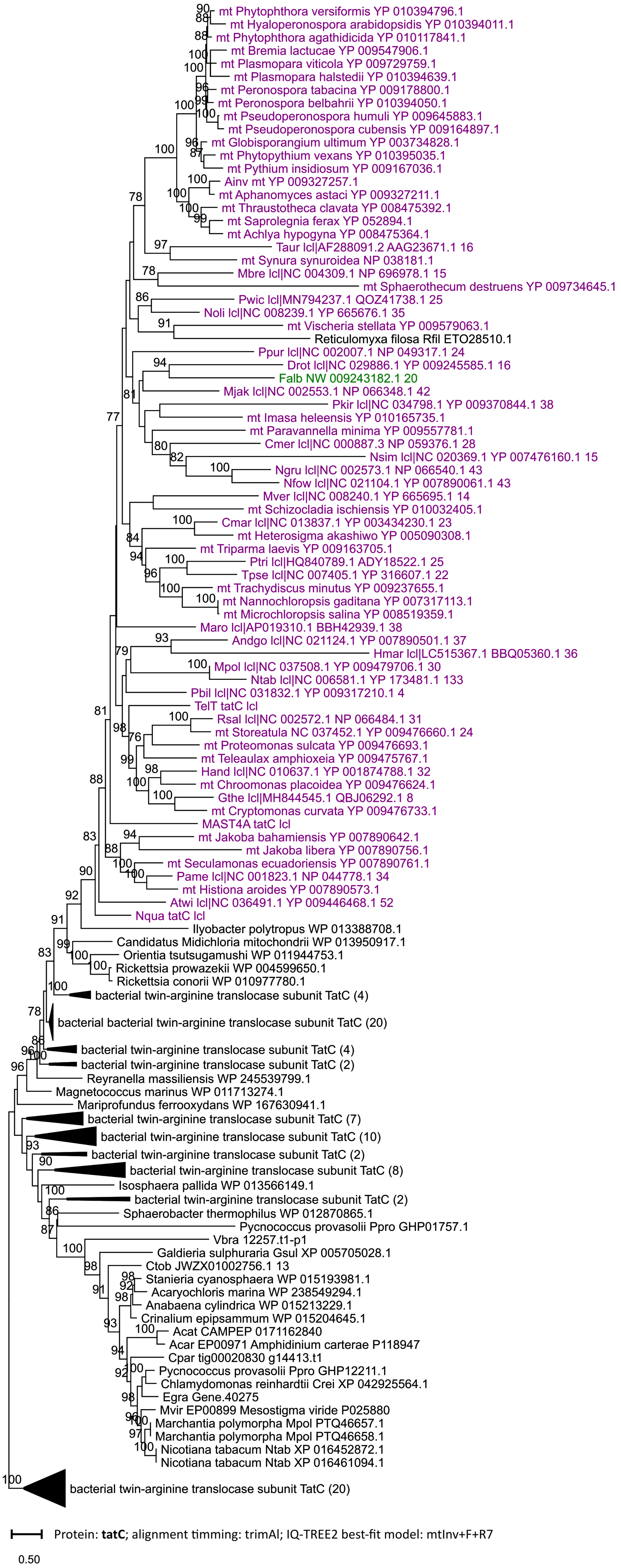

Protein: **tatC**; alignment timming: trimAl; IQ-TREE2 best-fit model: mtInv+F+R7

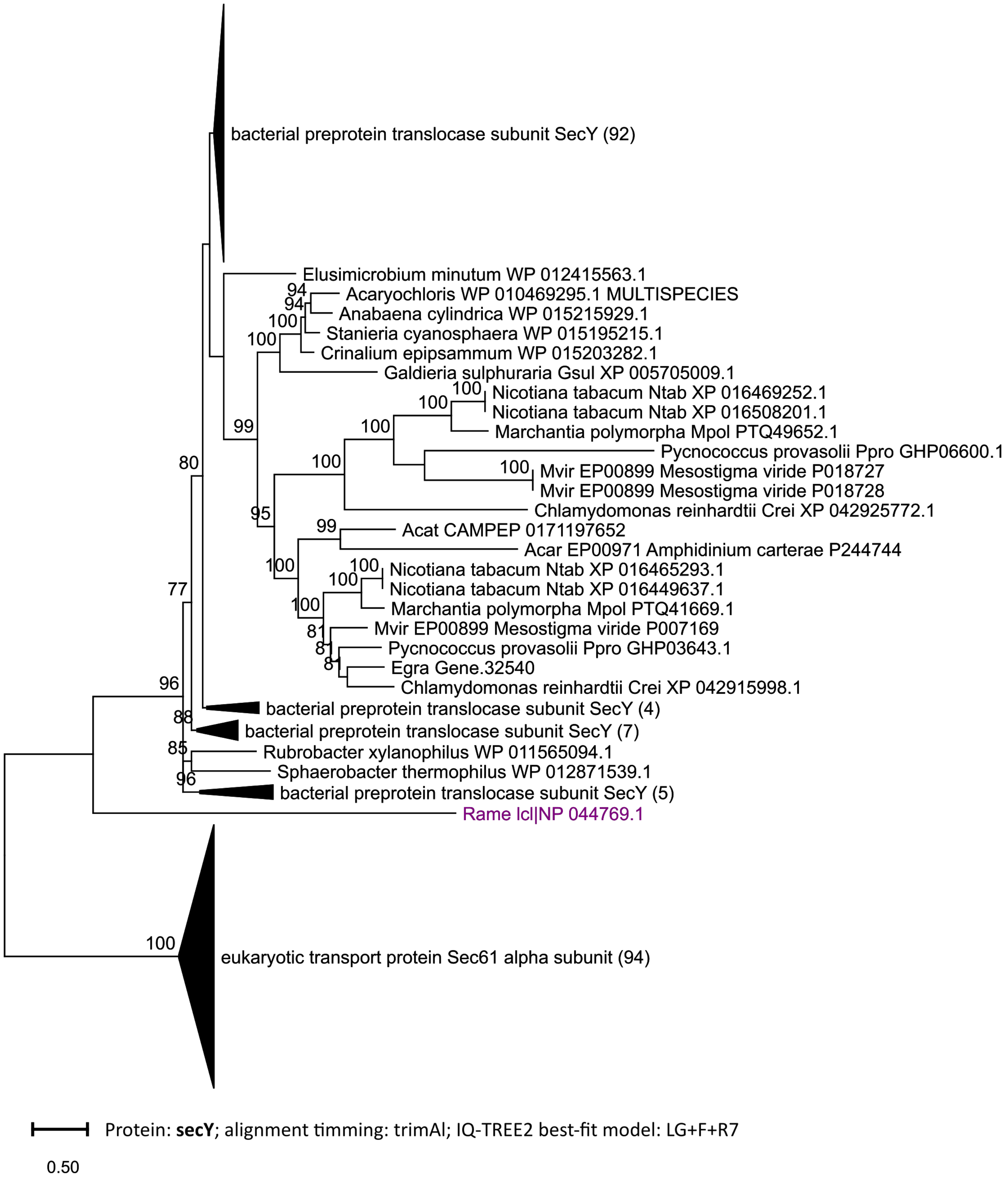

Protein: **secY**; alignment timming: trimAl; IQ-TREE2 best-fit model: LG+F+R7
